# Serum metabolomics identifies gut-derived uremic toxins and bile acid dysregulation associated with chronic kidney disease severity

**DOI:** 10.1038/s41598-026-44271-4

**Published:** 2026-04-14

**Authors:** Nehal Y. Mansour, Manal F. Ismail, Noha H. Sayed, Amira R. El-Ansary, Marwa Mohanad

**Affiliations:** 1 Department of Biochemistry, Faculty of Pharmacy, Cairo University, Cairo, Egypt; 2 Department of Biochemistry, College of Pharmaceutical Sciences and Drug Manufacturing, Misr University for Science and Technology (MUST), 6th of October City, Giza Egypt; 3 Department of Internal Medicine, Faculty of Medicine, Misr University for Science and Technology (MUST), 6th of October City, Giza Egypt

**Keywords:** Bile acid metabolism, Gut-kidney dysbiosis, Metabolic reprogramming, Tryptophan-kynurenine pathway, Uremic toxins, Biochemistry, Biomarkers, Diseases, Nephrology

## Abstract

Chronic kidney disease (CKD) affects more than 700 million people worldwide, however conventional biomarkers like creatinine cannot identify early-stage disease or accurately predict progression. In this study, untargeted and targeted metabolomic approaches were combined to identify novel biomarkers relevant for CKD staging and early detection in an underrepresented Egyptian population. Untargeted ultra-high-performance liquid chromatography–mass spectrometry analyses in both ionization modes were performed on serum samples of 50 CKD patients [25 early-stage CKD (eCKD), 25 end-stage kidney disease (ESKD)] and 20 controls. Differential metabolites were determined by univariate and multivariate analyses, coupled with pathway analysis and correlations with estimated glomerular filtration rate (eGFR). Five discriminating metabolites (p-hydroxyphenyllactic acid, indoxyl sulfate, xanthurenic acid, trimethylamine-N-oxide, and glycochenodeoxycholate) were subjected to targeted LC-MS/MS validation in an independent cohort (35 eCKD, 35 ESKD, 15 controls). Gut-derived uremic toxins, bile acid and tryptophan–kynurenine metabolic dysregulation were associated with CKD severity. p-hydroxyphenyllactic acid, xanthurenic acid, glutamyl-valine and indoxyl sulfate showed strong inverse correlations with eGFR (*r* = -0.75 to -0.85). A five-metabolite panel (indoxyl sulfate, p-hydroxyphenyllactic acid, trimethylamine-N-oxide, glycochenodeoxycholate, xanthurenic acid), demonstrated superior discriminatory performance compared with creatinine alone for distinguishing ESKD, especially indoxyl sulfate and p-hydroxyphenyllactic acid (AUC 0.847 and 0.828, respectively vs. 0.688). This first comprehensive metabolomics study in Egyptian CKD patients identifies alterations in gut microbiome–derived metabolites and bile acid metabolism associated with CKD severity. The multi-metabolite panel demonstrates potential for non-invasive discrimination between CKD stages and supports future longitudinal metabolomic studies aimed at improving CKD risk stratification and patient management.

**Supplementary Information:**

The online version contains supplementary material available at 10.1038/s41598-026-44271-4.

## Introduction

Chronic kidney disease is a condition where the kidneys gradually lose their ability to function properly or suffer damage that lasts for more than three months^1^. This condition has become worldwide affecting around 700 million people in 2019, with a prevalence rate of 9.1% and 3.16 million deaths^2^. The prevalence increases to 47% in individuals over 70 years of age^3^. In some parts of Asia kidney disease isn’t as big of a problem as it used to be. However, countries, like South Africa, Egypt and Mexico have experienced more individual get sick or die from kidney problems^2^. By 2040 kidney disease is expected to be one of the reasons, for lost years of life ranking fifth in terms of overall impact^4^.

The Kidney Disease Improving Global Outcomes (KDIGO) organization divides CKD into five distinct stages. This classification is based on two factors: the estimated glomerular filtration rate (eGFR) and the presence of albumin in urine^5^. CKD may progress through different stages to end-stage kidney disease (ESKD), at which point patients often require dialysis or kidney transplantation, both of which carry substantial clinical and economic burdens^6^. Early detection remains challenging because CKD is frequently asymptomatic in its early stages^7^.

In current clinical practice, CKD assessment relies mainly on serum creatinine, eGFR, and albuminuria. Although indispensable, these markers have important limitations: they are influenced by non-renal factors such as age, muscle mass, and ethnicity; they often rise only after substantial kidney damage has occurred; and they offer limited insight into the underlying pathophysiological processes, which may lead to misclassification of disease severity^8,9^. The development and advancement of CKD are also influenced by a complex interaction of environmental factors, dietary habits, the presence of infections, and genetic predisposition. Gene-environmental interactions and early-life environmental exposures contribute to disease risk in the populations. These considerations are particularly relevant in Egypt, where environmental exposures and genetic backgrounds may differ from those in other regions^10,11^. Consequently, there is a critical need for biomarkers that reflect CKD-related molecular alterations and may support improved disease stage discrimination and monitoring. Metabolomics provides such an approach by characterizing disease-related metabolic alterations and identifying perturbed biochemical pathways^12,13^.

The study of particular groups of people becomes crucial because CKD displays distinct symptoms and disease progression patterns among different ethnic populations^14^. The application of metabolomics has led to the discovery of biomarkers which help detect early stages of CKD and distinguish between different stages of the disease^12,15^. The use of untargeted metabolomics for studying CKD in the Egyptian population has not received sufficient investigation.

This study aims to address the gap in CKD research in Egypt by utilizing untargeted metabolomics to identify metabolic alterations across CKD stages. This approach supports the identification of candidate biomarkers for disease stage discrimination and expands the understanding of CKD-related metabolic changes in diverse populations.

## Subjects and methods

### Participant selection criteria

This study included 70 participants: 50 CKD patients were enrolled at different stages, and 20 healthy controls (NC) for the untargeted metabolomics. CKD patients were consecutively recruited from the department of internal medicine at Kasr Al Ainy Hospital, Cairo University. Eligibility was assessed through medical record review and clinical evaluation prior to enrolment, followed by application of predefined inclusion and exclusion criteria. The study included participants aged 18 and above who had confirmed CKD diagnosis of at least three months duration from different causes. The research study excluded participants who were pregnant or had urinary tract infections or kidney stones or thyroid disorders or active liver cirrhosis or ascites or were taking steroids or immunosuppressants or nephrotoxic drugs or angiotensin-converting enzyme inhibitors or angiotensin receptor blockers. Information on diabetes mellitus, hypertension, and dialysis status was collected where available and is summarized by study group, including the missing data, in Supplementary Table S1. Healthy controls were recruited from hospital staff and the surrounding community volunteers. Individuals with a history of CKD, hypertension, diabetes, obesity (BMI ≥ 30 kg/m^2^), cardiovascular disease, or other major chronic illnesses were excluded. Routine evaluation also confirmed normal serum creatinine and eGFR values. CKD patients were subdivided into two groups based on eGFR values, using the CKD-EPI equation^16^: 25 were in early-stage CKD (eCKD), defined as CKD stage 2-3with an eGFR 30–89 ml/min/1.73 m^2^ and not receiving renal replacement therapy and 25 were in end-stage kidney disease (ESKD), defined as CKD stage 4–5 with an eGFR < 15 ml/min/1.73 m^2^ and eligible or receiving renal replacement therapy. The term ESKD corresponds to kidney failure according to KDIGO terminology. An independent cohort of 85 individuals including 70 CKD patients (35 eCKD and 35 ESKD as stratified by CKD-EPI equation) and 15 matched NC was subjected to targeted metabolomic validation. To reduced potential confounding effects, age, gender, and BMI were matched between CKD patients and NC. The study was approved by the Research Ethics Committee, Faculty of Pharmacy, Cairo University (Approval No.BC [2984]). Written informed consent was obtained from all participants, and the study complied with the Declaration of Helsinki and Good Clinical Practice standards.

### Methods

#### Chemicals and reagents

Analytical and MS-grade acetonitrile, methanol, and formic acid, along with additional solvents for sample preparation (all analytical grade), were purchased from Merck Life Science (Sigma-Aldrich, KGaA, Darmstadt, Germany). Creatinine assay kit was obtained from Abcam (ab204537, Cambridge, UK). The analytical standards for p-hydroxyphenyllactic acid (p-HPhLA) (CAS# 306-23-0), trimethylamine-N-oxide (TMAO) (CAS#593-81-7), indoxyl sulfate (IS) (CAS#2642-37-7), glycochenodeoxycholate (GCDCA) (CAS#640-79-9), and xanthurenic acid (XA) (CAS#59-00-7) were acquired from Merck (Merck KGaA, Darmstadt, Germany). Stable-isotope labeled internal standards of p-HPhLA-d3 (TRC-H953667), TMAO-d9 (TRC-T795792), IS-d5 (TRC-I655101), GCDCA-d4 (CDN-D-5673) and XA-d4 (TRC-X743502) were purchased from LGC standards (Teddington, UK). Ultrapure water for HPLC was prepared using Milli-Q system from Millipore (Milford, USA). Analyte stock solutions (1 mg/mL) were generated by dissolving appropriate quantities of purified powders in MeOH/H_2_O (50:50) and stored at − 20 °C when not in use. All solutions were stored in amber glass containers certified for mass spectrometry from Waters (Milford, MA, USA) and protected from light. The internal standard working solution was a 10× concentrate in 50% MeOH.

#### Samples collection and storage

Subjects were required to fast for a period of 8–12 h before specimen collection to achieve consistency in metabolomic profiles. Venous blood samples (6 mL) were collected using sterile venipuncture techniques into serum separator tubes (SST). After collection, samples were left to clot at room temperature for 30 min., followed by centrifugation at 1500–2000 ×g (3000–4000 rpm) for 12 min. to isolate the serum. The resultant serum was then aliquoted and cryopreserved at -80 °C till further analysis.

#### Sample preparation and metabolites extraction

The experiments were done at the Central Laboratory at Faculty of Pharmacy, Cairo University. The frozen serum samples were thawed on ice to prevent the degradation of metabolites. Metabolites were extracted by adding 600µL of ice-cold methanol to 200µL of serum, vortexing for 2 min and incubating the mixture at -20 °C for 20 min to precipitate the proteins. The samples were then centrifuged at 25,759 ×g for 10 min at 4 °C and the supernatant was transferred to a new microcentrifuge tube. The remaining pellet was re-extracted with 600 µL of ice-cold methanol, followed by vortexing and centrifugation under the same conditions. The second supernatant was combined with the first extract, gently mixed, and centrifuged again at 25,759 × g for 5 min at 4 °C to remove residual debris. The combined extract was evaporated to dryness, then reconstituted in 100 µL of 50:50 acetonitrile: water and stored at − 80 °C until analysis. The analytical reproducibility was evaluated through the preparation of quality control (QC) samples which combined equal portions of all serum samples and underwent parallel processing.

Validation of differential metabolites between eCKD and ESKD was performed using internal standards. The calibration curve standards were prepared by diluting a pooled analyte standard stock solution within the range of expected physiological concentration levels. Extraction of metabolites was achieved by adding 10 µL of internal standard working solution, followed by precipitation with 3–4 volumes of acetonitrile in which samples were cooled at -20 °C for 30 min, then centrifuged at 15,000 × g for 10 min at 4 °C, dried as needed and reconstituted in 100 µL of 2:98 ACN: H2O. This allows final analytical concentrations chosen to quantify mid-endogenous levels of analytes as would be found in serum. LQC, MQC and HQC were established at 1/100, 1/20, and neat dilutions of working stock, respectively. MQC concentration was established to be 5x mean endogenous level. To each of the calibrators and QCs, internal standard solution was added, and they were all prepared the same way as the study samples.

#### Untargeted liquid chromatography and mass spectrometry analysis

Metabolite separation was performed on a Thermo Scientific Vanquish ultra-high performance liquid chromatography (UHPLC) system using a C18 reverse-phase column (Waters ACQUITY UPLC HSS T3, 100 Å, 1.8 μm, 2.1 mm × 100 mm). Water with 0.1% formic acid (solvent A) and acetonitrile with 0.1% formic acid (solvent B) were the mobile phase. Gradient elution condition was: 95:5 V/V (A: B) at 0 min, which was linearly changed to 5:95 V/V (A: B) between 1 and 15 min, maintained until 17 min, and then re-conditioned to 95:5 between 17.1 and 30 min. The flow rate was at 0.3 mL/min, and the injection volume was 5 µL, with column temperature kept constant at 40 °C. The eluted fractions were collected and available for mass spectrometry analysis.

Mass spectrometry was performed using a high-resolution XEFO G3 QTOF mass spectrometer instrument (Waters Corp., Milford, USA). The samples were recorded in reflector mode across a mass range of m/z 70–1000 in positive and negative ion modes. The collision induced dissociation in transfer cell (MS/MS) was used to fragment selected precursor ion by optimizing parameters to improve the efficiency of fragmentation while enhancing the spectrum resolution. Spectral data were processed using Waters MassLynx 4.2 software and exported as exported in mzXML format for downstream metabolite identification and annotation.

#### Targeted liquid chromatography and mass spectrometry analysis of the validation cohort

The following requirements facilitated the analysis of sample supernatants of the validation cohort as previously described^17^: UPLC column: Waters ACQUITY UPLC HSS T3 C18 (1.8 μm, 2:1 mm∗100 mm, 40 °C, 0.4mL/min flow rate, 2µL sample injection volume; solvent system: water: acetonitrile (0.04% acetic acid); gradient program: 0 min: 95:5 V/V, 11.0 min: 5:95 V/V, 12.0 min: 5:95 V/V, 12.1 min: 95:5 V/V, 14.0 min: 95:5 V/V.

The XEVO TQD triple quadruple mass spectrometer (Waters Corp., Milford, USA) equipped with an electrospray ionization (ESI) utilized for multiple reaction monitoring in positive and negative ion modes. This system ran Water MassLynx 4.2 software. The system operated with these ESI source parameters: source temperature: 500 °C, capillary voltage: 3.5 kV (+) and 3.0 kV (-), desolvation gas flow: 800 L/h, cone gas flow: 50 L/h. Argon was used as collision gas at 3.5 bar. Tuning and calibration of the apparatus were done using sodium iodide in accordance with manufacturer’s instructions. Multiple reaction monitoring transitions were selected for each time period depending on metabolites eluted.

Calibration curves were constructed employing six concentration levels for each metabolite. Linearity was assessed by plotting analyte-to-internal-standard peak area ratios against their respective nominal concentrations, utilizing weighted linear regression. The validated calibration range was determined based on both linearity and the absence of fit issues. The limits of detection (LOD) and quantification (LOQ) were determined using the formulas 3.3 × SD/slope and 10 × SD/slope, respectively (Supplementary Table S2).

#### Data processing

The WORKFLOW4METABOLOMICS subset of the Galaxy France platform (https://usegalaxy.fr/) processed for mzXML data file processing^18^. The XCMS tool was used for feature detection, alignment and integration. Peaks were first detected using the centWave algorithm, with parameters optimized for high-resolution data (peak width range, signal-to-noise threshold, m/z tolerance, and intensity filters). Retention-time (RT) deviation across samples was corrected using the obiwarp algorithm, followed by regrouping of aligned features to generate a unified peak table. A data matrix of peak intensities was then constructed across all samples. Missing intensities were handled using the fillChromPeaks function, after inspection of NA distributions. Metabolite annotation was performed using CAMERA, which grouped related adducts, isotopes, and fragments and assigned putative molecular features. Putative metabolite identities were subsequently matched by comparing accurate mass, and RT against reference databases, including HMDB, METLIN, and LIPID MAPS. Detailed processing parameters are provided in Supplementary Table S3**.**

#### Statistical analysis

MetaboAnalyst version 6.0 and R Studio version 4.3.1 were used for univariate and multivariate metabolic analysis of study groups. MetaboAnalyst power analysis showed test power was 0.75 and 0.95 for untargeted metabolomics and targeted validation, respectively at the given sample size^19^. Samples were normalized and data were log transformed, and autoscaled to ensure data normalization. ANOVA, followed by post-hoc Tukey’s HSD and Wilcoxon rank-sum tests, were used for intergroup comparisons. Volcano plots displayed significantly differentiated metabolites.

Multivariate models, principal component analysis (PCA), partial least squares discriminant analysis (PLS-DA), and orthogonal PLS-DA (OPLS-DA) were performed to distinguish groups. Model performance was evaluated with R^2^ (fit) > 1 and Q^2^ (cross-validation) > 0.5, indicating strong performance. A 100-permutation testing confirmed PLS-DA and OPLS-DA models. Models were considered valid when the observed Q^2^ value exceeded all permuted Q^2^ values and the intercept approached zero. Metabolites with variable importance in projection (VIP) > 1.2 were used to assess differentiation between groups. Enrichment, pathway, and network analysis tools were used to identify altered metabolic pathways, biological processes, and metabolite-metabolite interactions. Correlations between differential metabolites and eGFR were detected using spearman’s rank test. For both the untargeted discovery and targeted validation cohorts, p-values from univariate comparisons were adjusted for multiple testing using the false discovery rate (FDR) correction according to the Benjamini–Hochberg method. Statistical significance was defined as *p* < 0.05 and FDR < 0.05. The discriminatory performance of the validated metabolites was evaluated using receiver operating characteristic (ROC) curve analysis based on quantitative targeted metabolomics data.

## Results

### Biochemical characteristics

Table 1 displays the biochemical characteristics of the discovery cohort. All three groups (ESKD, eCKD, and NC) were comparable with respect to age, sex, BMI, and lipid profiles, including total cholesterol, HDL, LDL, and triglycerides. In contrast, ESKD patients had significantly lower hemoglobin, albumin, ionized calcium, and sodium and higher phosphorus, creatinine, and urea levels, and reduced eGFR, when compared to both eCKD and NC. The eCKD group showed mild reductions in albumin, sodium, and eGFR versus NC. The albumin-to-globulin ratio gradually decreased (NC > eCKD > ESKD), indicating the severity of the disease.

**Table 1:** Biochemical features of participants.

Parameters	Control (*n* = 20)	eCKD (*n* = 25)	ESKD (*n* = 25)
Age	52.95 ± 2.74	54 ± 7.75	57.36 ± 8.025
Male	9 (45.0%)	12 (48.0%)	14 (56.0%)
Female	11 (55.0%)	13 (52.0%)	11 (44.0%)
BMI	23.72 ± 4.74	24.94 ± 5.49	25.21 ± 5.78
HGB (g/dL)	12.93 ± 1.41	12.72 ± 1.36	10.10 ± 1.43^ab^
Ionized Calcium (mg/dL)	4.51 ± 0.21	4.33 ± 0.28	3.42 ± 0.28^ab^
Sodium (mEq/L)	139.5 ± 1.93	131.56 ± 2.08^a^	128.88 ± 2.51^ab^
Phosphorous (mg/dL)	3.49 ± 0.34	3.76 ± 0.33	4.77 ± 0.55^ab^
Albumin (g/dL)	4.1 ± 1.9	3.68 ± 0.26^a^	3.07 ± 0.29^ab^
Globulin (g/dL)	3.19 ± 0.14	3.57 ± 0.36^a^	3.28 ± 0.26^ab^
A/G ratio	1.27 ± 0.09	1.03 ± 0.05^a^	0.93 ± 0.03^ab^
Creatinine (mg/dL)	0.69 ± 0.15	1.80 ± 0.58	6.04 ± 2.42 ^ab^
Serum Urea (mg/dL)	28.60 ± 4.72	60.32 ± 11.52^a^	87.12 ± 6.09^ab^
eGFR (ml/min/1.73 m^2^)	106.3 ± 9.93	44.08 ± 15.43^a^	11.48 ± 6.28^ab^
ALT(U/L)	10.00 ± 5.25	12.20 ± 7.70	15.00 ± 7.40
AST (U/L)	17.65 ± 6.30	21.45 ± 11.65	24.22 ± 9.62
Total Cholesterol (mg/dL)	188.80 ± 60.08	217.36 ± 42.77	226.92 ± 64.27
HDL (mg/dL)	63.35 ± 16.36	59.40 ± 11.87	54.44 ± 11.06
LDL (mg/dL)	109.92 ± 12.12	116.46 ± 10.04	120.26 ± 19.25
Triglycerides (mg/dL)	88.70 ± 19.79	95.4 ± 10.14	100.4 ± 20.94

Data are presented as mean ± SD, or number (percentage). Data were analyzed using the one way ANOVA with Tukey’s post-hoc test or X^2^ test. ^a^ significant difference from control group, ^b^ significant difference from eCKD group. A/G: albumin/globulin, ALT: alanine transaminase, AST: aspartate transaminase, BMI: body mass index, eCKD: early stage chronic kidney disease, eGFR: estimated glomerular filtration rate, ESKD: end stage kidney disease, HDL: high density lipoprotein, HGB: hemoglobin, LDL: low density lipoprotein.

Targeted LC-MS/MS was performed on 85 individuals (35 ESKD, 35 eCKD, and 15 NC) to confirm untargeted metabolomic results. The validation group had similar biochemical features to the discovery cohort (Supplementary Table S4).

### Serum untargeted metabolomics

**Fig. 1:**
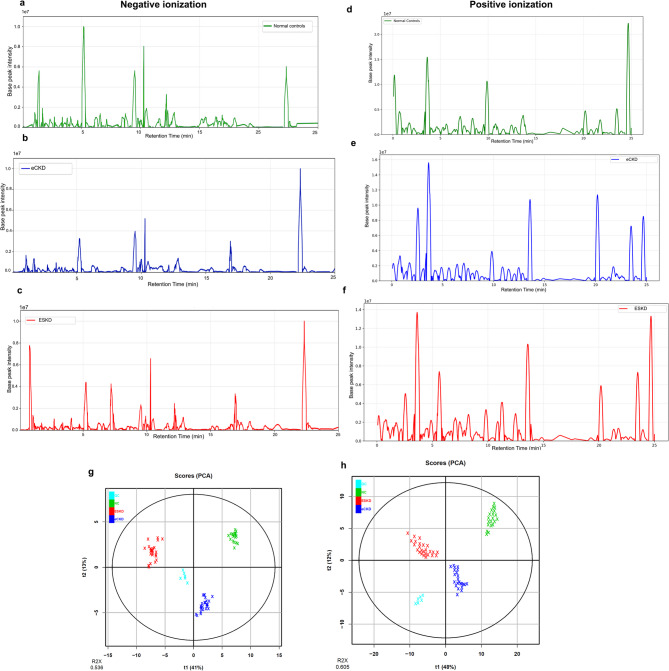
Metabolomic profile of NC, eCKD, and ESKD groups. (**a–f**) Representative total ion chromatograms (TICs) for negative (left panel) and positive (right panel) ionization modes for NC, eCKD, and ESKD groups, respectively. (**g**,**h**) Principal component analysis (PCA) of the metabolomic profiles for negative (**g**) and positive (**h**) ionization modes. PCA score plots indicate the discrimination of ESKD, eCKD, and NC groups and QC samples. The proximity clustering of QC samples exhibits analytical stability and reproducibility. The model explains 60.5% (R^2^X = 0.605) and 53.6% (R^2^X = 0.536) of the cumulative variance in the positive and negative ionization modes, respectively, showing a clear difference between groups. eCKD: early-stage chronic kidney disease, ESKD: end-stage kidney disease, NC: healthy controls, QC: quality control.

Figure 1 shows representative total ion chromatograms (TICs) for the NC, eCKD, and ESKD groups in both ionization modes. These indicate typical metabolomic profiles for each group. Panels a–c show -ve mode, whereas panels d–f show +ve mode. Untargeted metabolomics analysis identified 480 features in -ve mode and 684 features in +ve mode. The PCA score plots illustrate the differences between NCs, eCKD, and ESKD groups and QC samples in both ionization modes (Fig. 1g and h). A tight cluster pattern of QC samples indicates stable and reproducible analysis. The model accounts for 53.6% (R^2^X = 0.536) of the total variance in negative ionization mode and 60.5% (R^2^X = 0.605) in positive ionization mode. This shows that the groups are clearly different from each other.

### Comparative analysis of untargeted metabolic profiling across CKD stages and healthy controls

The serum metabolites of ESKD, eCKD, and NCs were compared by ANOVA with Tukey’s (HSD) post-test (Supplementary Table S5). Through −ve ionization mode, 79 endogenous metabolites differed statistically between groups (FDR < 0.05).

Post-hoc analysis revealed 11 metabolites p-HPhLA, creatinine, 5-dodecenoate (12:1n7), ornithine, succinic acid, asparagine (ASN), arginine (Arg), arachidonic acid, 4-vinylphenol sulfate, 2-hydoxystearate, and methionine (Met) were significantly different in all three pairwise comparisons (eCKD vs. NC, ESKD vs. NC and ESKD vs. eCKD).

Feruloylputrescine, oxalic acid, phosphocholine, citric acid, cinnamic acid, hypotaurine, myristoleate, malic acid, cholesteryl sulphate, hippurate, citrulline, adrenate, p-cresyl sulfate (PCS), glycolithocholate sulfate, ursodeoxycholate, aspartate, 2-keto tridecanoic Acid, xanthine, 17-methyloctadecanoate, glutamate, ceramide, N-acetylalanine, 10-heptadecenoate (17:1n7), nonadecanoate, eicosanoate, oleic acid-2,6-diisopropylanilide, and nonadecanal significantly differed between NC and eCKD and between NC and ESKD.

GCDCA, taurocholate, inosine, deoxycholic acid, acetyl carnitine, calcitriol, adipic acid, thymol sulfate, 3-hydroxydecanoate, lactate, indoxyl sulphate, N-formyl methionine, phenylalanine, kynurenic acid, phenol sulfate, 16-hydroxypalmitate, lithocholic acid, octadecanedioate (C18), thymidine, oleic acid, stearic acid, homocysteine, pyruvate, homovanillate, sarcosine, linoleic acid, heptanoate (7:0), Myo-inositol, hydroxyisocaproate, and Laurate (12:0) differed between eCKD and ESKD and between NC and ESKD.

Phenylacetate was significantly increased in eCKD but lower than in both NC and ESKD patients. Decanoic acid, catechol sulphate, and palmitoleate (16:1n17) significantly differed between NC and eCKD. Undecanoic acid, 3-hydroxyisovalerate, hydrochlorothiazide, and myristate significantly differed between NC and ESKD. Metabolic profiling also identified individual metabolic features differentiating ESKD from eCKD: 2-hydroxyisovalerate, 4-methyl-2-oxopentanoate, and 3-methyl-2-oxovalerate.

Using the +ve ionization mode, 112 endogenous metabolites were statistically different between the groups (FDR < 0.05). Post-hoc Tukey’s HSD identified 45 significantly different serum metabolites across all pairwise comparisons. These included phenylacetylglutamine, trimethylamine N-oxide, methylguanidine, threonine, lysophosphatidylcholine (18:1), creatinine, anthranilate, urea, pseudouridine, methylmalonate, taurine, N-acetylneuraminic acid, 3-phenylpropionate, adenine, dimethylarginine (DMA), betaine, methionine sulfoxide, allantoin, theobromine, glycerophosphoryl choline, dimethylglycine (DMG), valine, paraxanthine, porphobilinogen, suberate, cystine, cortisol, butyrylcarnitine, cortisone, theophylline, N6-methyllysine, tyrosine, N-acetylornithine, tryptophan, dihydrocapsaicin, diethanolamine, uric acid, histidine, N-acetylglutamine, β-hydroxybutyrate, gamma-glutamyl-phenylalanine, acetone, PCS, 4-hydroxyphenyl acetic acid, and cysteinyl-glycine.

Ascorbate, docosahexaenoic acid, lactose, 4-pyridoxate, palmitic acid, palmitoylcarnitine, xanthosine, argininosuccinic acid, glycyl-tyrosine, lysyl-glutamate, sphingomyelin, 3-methylhistidine, dihydrobiopterin, choline, 5-oxoproline, 3-methoxytyrosine, hydroxyproline, glucose, orotate, niacinamide, spermine, gluconic acid, glucosamine, α-ketoglutarate, glycerol 3-phosphate, 1-methylxanthine, leucine, and isoleucine significantly differed between NC and eCKD and between NC and ESKD.

Homoarginine (hArg), indolepropionate (IPA), and proline significantly differed between NC and eCKD and between eCKD and ESKD. 3-hydroxyanthranilate, glycyl-proline, Lysyl-proline, homogentisate, glycyl-valine, XA, pipecolate, 5-hydroxyindoleacetic acid, N-hydroxy-valine, biliverdin, aspartyl-phenylalanine, 2-phenylglycine, L-glutamine, glycocholate, glutamyl-valine, prolyl-leucine, norleucine, hydroquinone, prolyl-lysine, serine, 1-aminobutyrate, prolyl-tyrosine, kynurenic acid, cytosine, gamma-glutamyl-methionine, and serotonin distinguished ESKD from eCKD and NCs.

**Fig. 2:**
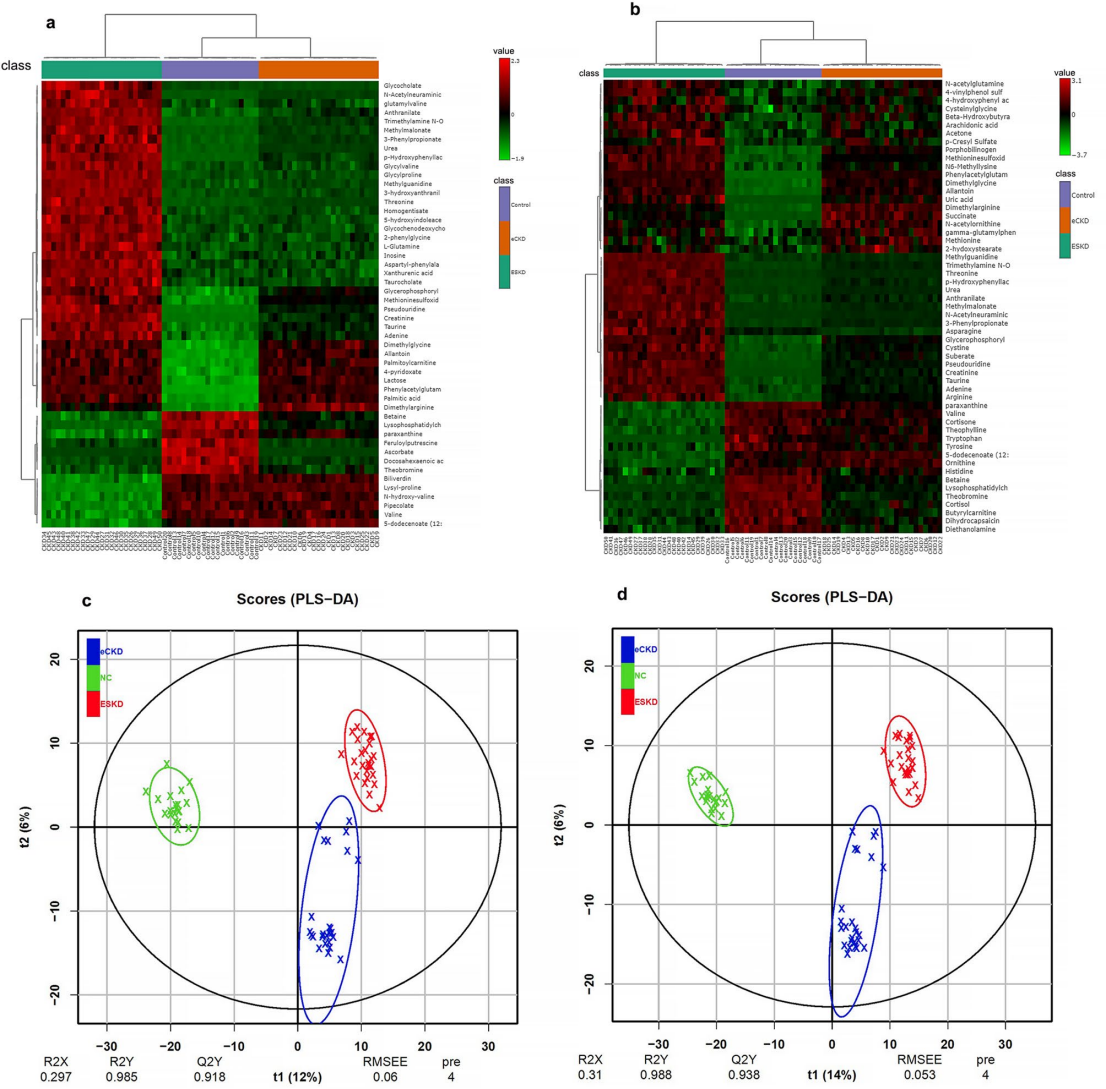
Alteration of serum metabolites in healthy controls, early-stage CKD, and end-stage kidney disease detected in −ve and +ve mode. (**a**) Heatmap of the top fifty disrupted metabolites across the three groups. These metabolites were identified by a one-way ANOVA (*p* < 0.05, FDR-corrected) to identify between-group variance. (**b**) Heatmap showing significant post-hoc differences in all pair-wise comparisons across the groups. Differential metabolites were detected by one way ANOVA followed by post-hoc Tukey’s HSD test for pairwise differences between NC, eCKD, and ESKD. (**c**) A PLS-DA score plot showing the separation between the three groups based on the metabolites detected in negative ionization mode (R^2^X: 0.297, R^2^Y: 0.985, Q^2^Y: 0.918 with 100 permutations). (**d**) PLS-DA score plot for the separation of the three groups based on metabolites observed in positive ionization mode (R^2^X: 0.31, R^2^Y: 0.988, Q^2^Y: 0.938; 100 permutations).

Gamma-glutamyl-tyrosine, pantothenic acid, glutathione, hexanoylcarnitine, gamma-glutamyl-leucine, bilirubin, indoleacetate, malonate, carnosine, and vitamin B2 distinguished ESKD from NCs. These results indicate that CKD stage and severity are linked to certain metabolic changes. The most 50 differentially perturbed metabolites that differentiate among the three groups are graphically represented in a heatmap (Fig. 2a). The metabolites with significantly different pairwise group comparisons are also shown in another heatmap (Fig. 2b).

PLS-DA score plots showed clustering patterns, and significant separation between the NC, eCKD and ESKD groups. The ESKD group had different metabolic profiles than eCKD. In −ve ionization mode, R^2^X, R^2^Y, and Q^2^Y of PLS-DA model were 0.297, 0.985, and 0.918 (Fig. 2c), respectively and in +ve ionization mode, they were 0.31, 0.988, and 0.938, respectively, indicating significant discrimination between the three groups and optimum model perfomance (Fig. 2d). The permutation testing (100 permutations) confirmed that the model was not overfitted, as the observed Q^2^ value exceeded all permuted values (*p* < 0.01, Supplementary Fig. 1a-b).

### Distinct metabolomic alteration in CKD patients compared to apparently healthy controls in untargeted cohort

**Fig. 3:**
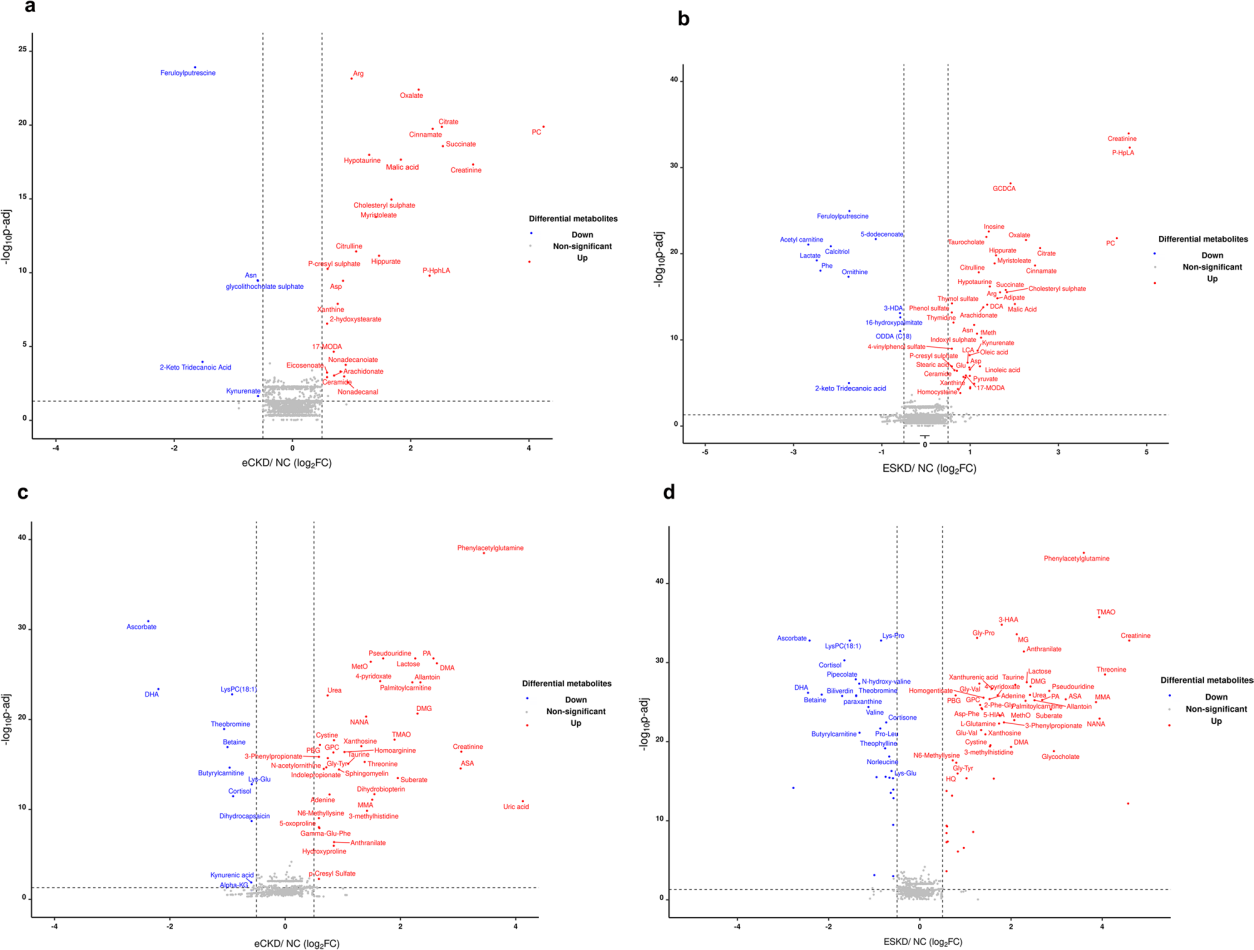
Volcano plots of metabolite distinctions between healthy controls (NC) and chronic kidney disease (CKD) groups under negative and positive ionization modes. (**a**) In negative ionization mode, 31 metabolites significantly differed between NC and eCKD. (**b**) In negative ionization mode, 56 metabolites significantly differed between NC and ESKD. (**c**) In positive ionization mode, 50 metabolites significantly differed between NC and eCKD. (**d**) In positive ionization mode, 85 metabolites significantly differed between NC and ESKD. The x-axis represents the log_2_ fold change between patient and control groups, and the y-axis represents the –log10 of the adjusted *p*-value. Red dots indicate significantly upregulated metabolites (log_2_FC ≥ 0.58), and blue dots indicate significantly downregulated metabolites (log₂ FC≤ -0.58) (adjusted *p* < 0.05). Statistical testing was performed with the Wilcoxon rank-sum test, and Benjamini–Hochberg correction. 2-Phe-Gly: 2-phenylglycine, 3HAA: 3-hydroxyanthranilate, 3-HDA: 3-hydroxydecanoate, 17-MODA: 17-methyloctadecanoic acid, Alpha-KG: alpha-ketoglutarate, Arg: arginine, ASA: argininosuccinic acid, Asn: asparagine, Asp: aspartate, Asp-Phe: Aspartyl-phenylalanine, DCA: deoxycholic acid, DHA: docosahexaenoic acid, DMA: dimethylarginine, DMG: dimethylglycine, fMeth: N-formyl methionine, Gamma-Glu-Phe: gamma-glutamyl-phenylalanine, GCDCA: glycochenodeoxycholic acid, Glu: glutamate, Glu-Val: glutamyl-valine, Gly-Tyr: glycyl-tyrosine, GPC: Glycerophosphoryl choline, HQ: hydroquinone, LCA: lithocholate, Lys-Glu: lysyl-glutamate, LysPC(18:1): lysophosphatidylcholine (18:1), Lys-Pro: lysyl-proline, MetO: methionine sulfoxide, MG: methylguanidine, MMA: methyl malonic acid, NANA: N-acetyl neuraminic acid, ODDA (C18): octadecanedioate (C18), PA: palmitic acid, PBG: porphobilinogen, PC: phosphocholine, P-HPhLA: p-hydroxyphenyllactic acid, Phe: phenylalanine, Pro-Leu: prolyl-leucine, TMAO: Trimethylamine N-oxide.

Pairwise comparisons of the untargeted discovery cohort were employed to investigate metabolite changes between eCKD vs. NC and ESKD vs. NC groups. Metabolites with FC ≥ 1.5 and FDR < 0.05 were significant (Supplementary Table S6). Using the −ve ionization mode, univariate analysis identified 31 metabolites that discriminated significantly between NC and eCKD, with 26 upregulated and 5 downregulated metabolites in eCKD. Arg, oxalate, phosphocholine, citrate, cinnamate, succinate, hypotaurine, malic acid, creatinine, and cholesteryl sulphate were the top upregulated metabolites in eCKD, whereas feruloylputrescine, Asn, and glycolithocholate sulphate were the top downregulated (Fig. 3a). There were 56 significantly differentiated metabolites between NC and ESKD, 46 upregulated, and 10 downregulated metabolites in ESKD. Creatinine, p-HPhLA, GCDCA, inosine, taurocholate, phosphocholine, oxalate, citrate, hippurate, and myristoleate were the top upregulated metabolites in ESKD. Feruloylputrescine, calcitriol, acetyl carnitine, calcitriol, lactate, and phenylalanine were the top downregulated (Fig. 3b).

Univariate analysis in the +ve ionization mode identified 50 metabolites that significantly differentiated eCKD from NC, with 39 up-regulated, and 11 down-regulated metabolites in eCKD. Phenylacetylglutamine, lactose, pseudouridine, palmitic acid, methionine sulfoxide, DMA, 4-pyridoxate, palmitoylcarnitine, allantoin, and urea were the top upregulated metabolites in eCKD. Ascorbate, docosahexaenoic acid, lysophoshatidylcholine (18:1), theobromine, and betaine were the most downregulated (Fig. 3c). Eighty-five metabolites were significantly altered in ESKD compared to NC. Fifty-six of which were upregulated and 29 downregulated in ESK. Phenylacetylglutamine, trimethyl-N-oxide, 3-hydroxyanthranilate, methylguanidine, Gly-Pro, creatinine, anthranilate, threonine, lactose, Glycyl-valine, taurine, DMG, XA, pseudouridine, and urea were the most significantly upregulated metabolites in ESKD, while Lysyl-proline, lysophophatidylcholine (18:1), ascorbate, cortisol, and pipecolate, were the most downregulated metabolites (Fig. 3d).

Table 2 provides a summary of major metabolites that were found to be statistically different between NC and eCKD, and NC and ESKD, with VIP scores > 1.2. The table displays chemical formulae of metabolites, regulation of metabolites in eCKD and ESKD patients versus NC, and corresponding metabolic pathways.

**Table 2:** Top 20 metabolites showing statistical significance and highest VIP scores differentiating eCKD and ESKD from healthy controls in negative and positive ionization mode.

Metabolite	mode	m/z	RT (min)	Formula	VIP	Groups	Up/Down	FDR	FC	Biochemical pathway
Arginine	−ve	175.10	2.60	C_6_H_14_N_4_O_2_	1.713	eCKD vs. NC	Up	7.034E−24	2.0	Urea cycle, Amino acid metabolism
Feruloyl putrescine	−ve	317.20	7.40	C_14_H_20_N_2_O_3_	1.712	eCKD vs. NC	Down	1.213E−24	0.32	Polyamine metabolism, Ferulate metabolism
					1.380	ESKD vs. NC	Down	2.152E−24	0.30	
Oxalic acid	−ve	89.00	1.20	C_2_H_2_O_4_	1.694	eCKD vs. NC	Up	3.957E−23	4.39	Amino acid metabolism
					1.362	ESKD vs. NC	Up	2.223E−21	4.81	
Phosphocholine	−ve	184.10	4.40	C_5_H_15_NO_4_P	1.676	eCKD vs. NC	Up	1.278E−20	18.98	Phospholipid metabolism
					1.364	ESKD vs. NC	Up	1.746E−21	20.016	
Citric acid	−ve	191.00	2.70	C_6_H_8_O_7_	1.674	eCKD vs. NC	Up	1.31E−20	5.76	TCA Cycle
					1.353	ESKD vs. NC	Up	1.432E−20	6.00	
Cinnamic acid	−ve	147.00	3.30	C_9_H_8_O_2_	1.670	eCKD vs. NC	Up	1.782E−20	5.17	Phenylpropanoid metabolism
					1.332	ESKD vs. NC	Up	1.141E−18	5.54	
Succinic acid	−ve	117.0	2.00	C_4_H_6_O_4_	1.658	eCKD vs. NC	Up	2.672E−19	5.83	TCA Cycle
Hypotaurine	−ve	108.01	1.80	C_2_H_7_NO_2_S	1.648	eCKD vs. NC	Up	1.026E−18	2.46	Taurine and hypotaurine metabolism
Creatinine	−ve	112.10	6.90	C_4_H_7_N_3_O	1.643	eCKD vs. NC	Up	4.661E−18	8.32	Creatine metabolism Amino acid metabolism
					1.416	ESKD vs. NC	Up	8.488E−33	24.11	
Malic Acid	−ve	133.01	2.30	C_4_H_6_O_5_	1.635	eCKD vs. NC	Up	2.18E−18	3.56	TCA Cycle
Cholesteryl Sulphate	−ve	465.35	7.10	C_27_H_46_O_4_S	1.589	eCKD vs. NC	Up	1.10E−15	3.19	Steroid biosynthesis
					1.29	ESKD vs. NC	Up	1.02E−02	3.56	
Myristoleate	−ve	225.18	5.70	C_14_H_26_O_2_	1.573	eCKD vs. NC	Up	1.66E−14	2.66	Fatty acid metabolism
					1.332	ESKD vs. NC	Up	6.938E−19	2.94	
Citrulline	−ve	174.09	2.00	C_6_H_13_N_3_O_3_	1.493	eCKD vs. NC	Up	3.61E−12	2.11	Urea cycle
					1.318	ESKD vs. NC	Up	6.193E−18	2.29	
Hippuric acid	−ve	178.05	3.70	C_9_H_9_NO_3_	1.483	eCKD vs. NC	Up	7.09E−18	2.76	Amino acid metabolism
					1.347	ESKD vs. NC	Up	9.267E−20	3.00	
p-cresyl sulphate	−ve	187.01	8.74	C_7_H_8_O_4_S	1.454	eCKD vs. NC	Up	5.47E−11	1.51	Amino acid metabolism
p-hydroxyphenyl lactic Acid	−ve	181.10	3.50	C_9_H_10_O_4_	1.425	eCKD vs. NC	Up	1.58E−10	4.99	Amino acid metabolism
					1.413	ESKD vs. NC	Up	1.863E−31	24.512	
Asparagine	−ve	131.05	5.90	C_4_H_8_N_2_O_3_	1.418	eCKD vs. NC	Up	3.27E−10	0.665	Amino acid metabolism
Aspartate	−ve	132.00	2.10	C_4_H_7_NO_4_	1.412	eCKD vs. NC	Up	3.53E−10	1.808	Urea cycle, Amino acid metabolism, TCA Cycle
Glycolithocholate sulphate	−ve	255.63	6.03	C_26_H_43_NO_7_S	1.405	eCKD vs. NC	Up	3.53E−10	0.667	Bile acid metabolism
Xanthine	−ve	151.03	1.70	C_5_H_4_N_4_O_2_	1.345	eCKD vs. NC	Up	1.28E−08	1.699	Purine metabolism
Glycochenodeoxycholate	−ve	448.31	9.95	C_26_H_43_NO_5_	1.397	ESKD vs. NC	Up	1.765E−27	3.78	Bile acid metabolism
Inosine	−ve	267.07	1.23	C_10_H_12_N_4_O_5_	1.367	ESKD vs. NC	Up	4.095E−22	2.68	Purine metabolism
Taurocholate	−ve	514.28	6.67	C_26_H_45_NO_7_S	1.363	ESKD vs. NC	Up	1.461E−21	2.581	Bile acid metabolism
5-dodecenoate (12:1n7)	−ve	197.16	8.70	C_12_H_22_O_2_	1.359	ESKD vs. NC	Down	1.928E−21	0.45	Fatty acid metabolism
Calcitriol	−ve	416.63	25.40	C_27_H_44_O_3_	1.359	ESKD vs. NC	Down	9.579E−21	0.22	Vitamin D metabolism
Acetyl carnitine	−ve	202.11	2.40	C_9_H_17_NO_4_	1.353	ESKD vs. NC	Down	6.669E−21	0.158	Fatty acid metabolism
Lactate	−ve	89.03	1.80	C_3_H_6_O_3_	1.337	ESKD vs. NC	Down	3.33E−19	0.18	Glucose metabolism
Phenylalanine	−ve	164.07	5.20	C_9_H_11_NO_2_	1.318	ESKD vs. NC	Down	4.226E−18	0.19	Amino acid metabolism
Ornithine	−ve	132.09	6.30	C_5_H_12_N_2_O_2_	1.316	ESKD vs. NC	Down	1.941E−17	0.296	Urea cycle
Phenylacetylglutamine	+ve	265.12	8.07	C_13_H_16_N_2_O_3_	1.478	eCKD vs. NC	Up	2.238E−42	10.91	Amino acid metabolism
					1.297	ESKD vs. NC	Up	1.334E−44	12.09	
Ascorbate	+ve	177.10	1.20	C_6_H_8_O_6_	1.463	eCKD vs. NC	Down	1.669E−34	0.193	Vitamin C metabolism
					1.282	ESKD vs. NC	Down	1.643E−33	0.187	
Lactose	+ve	343.122	0.73	C_12_H_22_O_11_	1.452	eCKD vs. NC	Up	3.456E−30	4.78	Carbohydrate metabolism
					1.270	ESKD vs. NC	Up	3.047E−28	5.08	
Dimethylarginine	+ve	203.138	9.20	C_8_H_18_N_4_O_2_	1.449	eCKD vs. NC	Up	2.817E−29	6.20	Amino acid metabolism
Pseudouridine	+ve	245.08	5.80	C_9_H_12_N_2_O_6_	1.449	eCKD vs. NC	Up	5.169E−30	3.25	Pyrimidine metabolism
					1.267	ESKD vs. NC	Up	3.806E−27	7.15	
Methionine sulfoxide	+ve	166.05	0.76	C_5_H_11_NO_3_S	1.449	eCKD vs. NC	Up	1.625E−29	2.80	Amino acid metabolism
Palmitic acid	+ve	400.34	14.9	C_16_H_32_O_2_	1.449	eCKD vs. NC	Up	5.731E−30	5.95	Fatty acid biosynthesis
Palmitoylcarnitine	+ve	104.07	1.87	C_23_H_45_NO_4_	1.439	eCKD vs. NC	Up	5.007E−27	4.61	Fatty acid metabolism
Allantoin	+ve	159.06	1.56	C_4_H_6_N_4_O_3_	1.435	eCKD vs. NC	Up	5.299E−27	5.09	Purine metabolism
4-pyridoxate	+ve	184.059	0.71	C_8_H_9_NO_4_	1.435	eCKD vs. NC	Up	3.11E−27	3.13	Vitamin B6 metabolism
Lysophosphatidylcholine (18:1)	+ve	522.36	5.80	C_26_H_52_NO_7_P	1.431	eCKD vs. NC	Down	1.360E−25	0.529	Phospholipid metabolism
					1.285	ESKD vs. NC	Down	1.643E−33	0.344	
Docosahexaenoic acid	+ve	329.25	17.76	C_22_H_32_O_2_	1.430	eCKD vs. NC	Down	3.216E−26	0.218	Fatty acid metabolism
Urea	+ve	120.065	0.78	CH_4_N_2_O	1.428	eCKD vs. NC	Up	1.968E−25	1.67	Urea cycle
Dimethylglycine	+ve	61.023	2.70	C_4_H_9_NO_2_	1.415	eCKD vs. NC	Up	2.817E−29	6.20	One-carbon metabolism
					1.269	ESKD vs. NC	Up	1.041E−27	5.38	
N-Acetylneuraminic Acid	+ve	310.12	1.20	C_11_H_19_NO_9_	1.411	eCKD vs. NC	Up	5.059E−23	2.65	Sialic acid metabolism
Theobromine	+ve	181.07	1.72	C_7_H_8_N_4_O_2_	1.396	eCKD vs. NC	Down	1.316E−21	0.48	Purine metabolism
Trimethylamine N-Oxide	+ve	76.08	0.85	C_3_H_9_NO	1.387	eCKD vs. NC	Up	2.105E−20	3.73	Choline metabolism
					1.289	ESKD vs. NC	Up	1.796E−36	15.28	
Cystine	+ve	241.03	2.46	C_6_H_12_N_2_O_4_S_2_	1.383	eCKD vs. NC	Up	2.588E−20	1.80	Amino acid metabolism
Betaine	+ve	118.09	1.80	C_5_H_11_NO_2_	1.38	eCKD vs. NC	Down	1.747E−19	0.50	One-carbon metabolism and amino acid metabolism
Porphobilinogen	+ve	227.102	6.88	C_10_H_14_N_2_O_4_	1.376	eCKD vs. NC	Up	9.019E−20	1.52	Porphyrin metabolism
Xanthosine	+ve	285.08	1.13	C_10_H_12_N_4_O_6_	1.374	eCKD vs. NC	Up	1.312E−19	2.50	Purine metabolism
3-hydroxyanthranilate	+ve	154.049	5.42	C_7_H_7_NO_3_	1.289	ESKD vs. NC	Up	1.626E−35	3.47	Tryptophan metabolism
Methylguanidine	+ve	74.071	6.88	C_2_H_7_N_3_	1.288	ESKD vs. NC	Up	2.614E−34	4.36	Amino acid metabolism
Glycyl-proline	+ve	173.092	6.54	C_7_H_12_N_2_O_3_	1.287	ESKD vs. NC	Up	7.574E−34	2.39	Peptide metabolism
Lysyl-proline	+ve	355.65	6.58	C_11_H_21_N_3_O_3_	1.286	ESKD vs. NC	Down	1.643E−33	0.55	Peptide metabolism
Creatinine	+ve	114.06	0.62	C_4_H_7_N_3_O	1.285	ESKD vs. NC	Up	1.643E−33	24.11	Amino acid metabolism
Anthranilate	+ve	120.04	8.62	C_7_H_7_NO_2_	1.282	ESKD vs. NC	Up	3.950E−32	4.86	Tryptophan metabolism
Cortisol	+ve	363.22	7.70	C_21_H_30_O_5_	1.278	ESKD vs. NC	Down	5.214E−31	0.32	Steroid hormone biosynthesis
Threonine	+ve	75.06	0.85	C_4_H_9_NO_3_	1.275	ESKD vs. NC	Up	3.178E−29	16.68	Amino acid metabolism
Pipecolate	+ve	130.09	1.19	C_6_H_11_NO_2_	1.272	ESKD vs. NC	Down	1.296E−28	0.38	Amino acid metabolism
Glycyl-valine	+ve	175.11	2.33	C_7_H_14_N_2_O_3_	1.269	ESKD vs. NC	Up	4.593E−28	2.47	Peptide metabolism
N-hydroxy-valine	+ve	134.08	1.59	C_5_H_11_NO_3_	1.269	ESKD vs. NC	Down	4.586E−28	0.398	Amino acid metabolism
Taurine	+ve	126.02	0.72	C_2_H_7_NO_3_S	1.268	ESKD vs. NC	Up	6.390E−28	4.27	Amino acid metabolism
Xanthurenic acid	+ve	206.04	2.39	C_10_H_7_NO_4_	1.266	ESKD vs. NC	Up	1.937E−27	3.00	Tryptophan metabolism

Statistical comparisons were performed using the Wilcoxon rank-sum test, with p-values adjusted for multiple testing using the Benjamini-Hochberg FDR. Metabolites with FDR-adjusted *p* < 0.05, FC ≥ 1.5 or ≤ 0.67, and VIP ≥ 1.2 (from OPLS-DA) were considered significant. eCKD: early-stage chronic kidney disease, ESKD: end-stage kidney disease, FC: fold change, FDR: false discovery rate, m/z: mass-to-charge ratio, NC: healthy controls, RT: retention time, VIP: variance of importance projection.

**Fig. 4:**
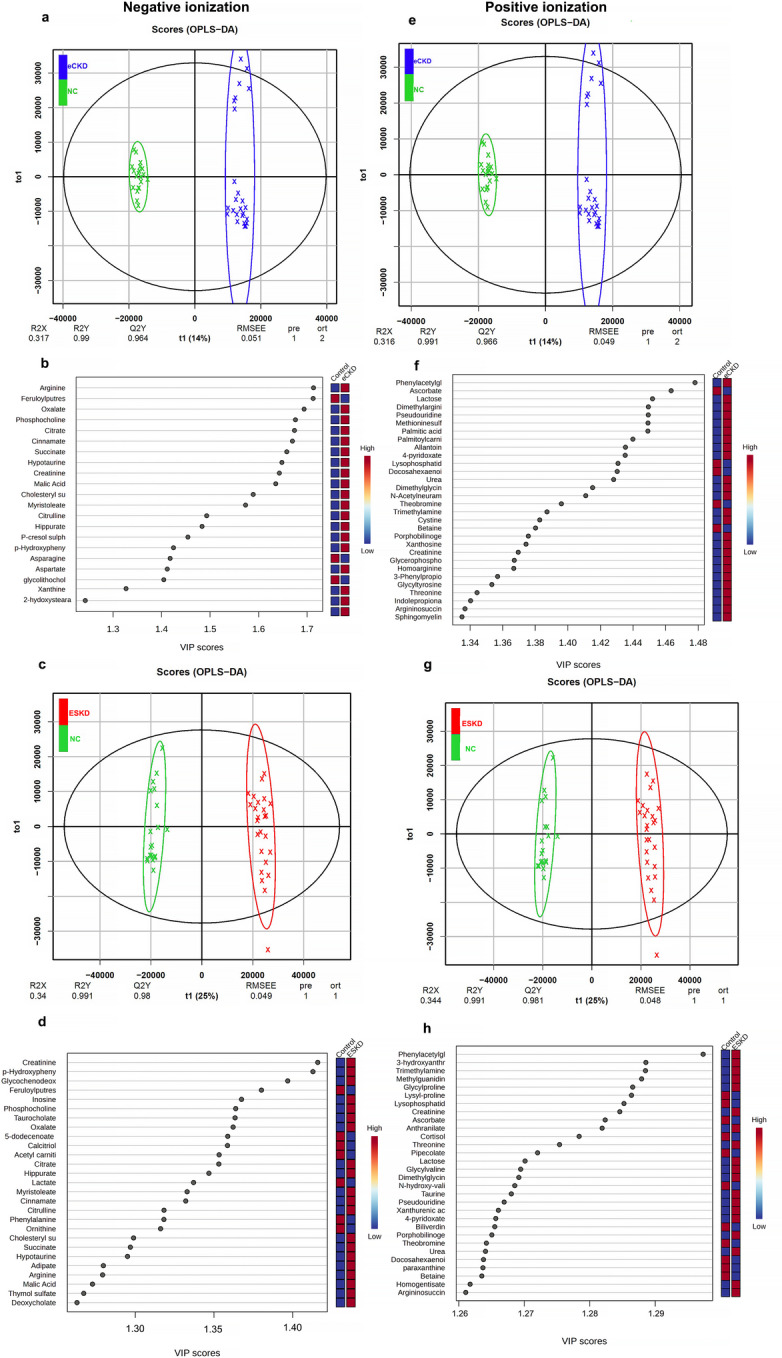
Multivariate analysis of the metabolites characterized in negative (left panel) and positive (right panel) ionization modes, where there are distinct metabolic profiles in healthy controls (NC), early-stage CKD (eCKD), and end-stage kidney disease (ESKD). (**a**) OPLS-DA score plot in -ve mode differentiating between NC and eCKD (R^2^X = 0.317, R^2^Y = 0.990, and Q^2^ = 0.964; 100 permutations). (**b**) Most discriminative metabolites (VIP > 1.2) in -ve mode that distinguish eCKD from NC, with relative abundance reported for each group. (**c**) OPLS-DA score plot in -ve mode differentiating between NC and ESKD (R^2^X = 0.340, R^2^Y = 0.991, Q^2^ = 0.980; 100 permutations). (**d**) Top discriminatory metabolites (VIP > 1.2) between ESKD and NC in -ve mode. (**e**) OPLS-DA score plot in +ve mode differentiating between NC and eCKD (R^2^X = 0.316, R^2^Y = 0.991, Q^2^ = 0.966; 100 permutations). (**f**) Top 30 most important metabolites (VIP > 1.2) in +ve mode distinguishing between NC and eCKD. (**g**) OPLS-DA score scatter plot in +ve mode differentiating between NC and ESKD (R^2^X = 0.344, R^2^Y = 0.991, Q^2^ = 0.981; 100 permutations). (**h**) Top 30 most important metabolites (VIP > 1.2) distinguishing between NC and ESKD using +ve mode. Statistical modeling and multivariate analysis were conducted with MetaboAnalyst 6.0. Metabolite selection was based on variable importance in projection (VIP) scores, where VIP quantifies the contribution of each metabolite to class separation within the OPLS-DA model; metabolites with VIP > 1.2 were considered important feature. Model validation was performed using 100 permutation tests to assess robustness and prevent overfitting. R^2^X, R^2^Y reflects the model interpretation rate, and Q^2^ reflects cross-validated model performance.

OPLS-DA model was used for multivariate analysis to discriminate between NC and eCKD and between NCand ESKD, for metabolites detected in both ionization modes (Fig. 4). In the −ve mode, the model had R^2^X, R^2^Y, and Q^2^ values of 0.317, 0.99 and 0.964, respectively, indicating a significant discrimination between the NC and eCKD (Fig. 4a). The permutation testing (100 permutations) confirmed that the model was not overfitted, as the observed Q^2^ value exceeded all permuted values (*p* < 0.01; Supplementary Fig. 1c). The model identified 21 metabolites with VIP > 1.2 that significantly differed between the NC and eCKD. Arg, feruloylputrescine, oxalic acid, phosphocholine, citric acid, cinnamic acid, hypotaurine, creatinine, malic acid, and myristoleate had the highest VIP values (Table 2; Fig. 4b). For the discrimination between NC and ESKD, the OPLS-DA model produced R^2^X, R^2^Y, and Q^2^ values of 0.34, 0.991 and 0.98, respectively (Fig. 4c). The permutation testing (100 permutations) confirmed that the model was not overfitted, as the observed Q^2^ value exceeded all permuted values (*p* < 0.01; Supplementary Fig. 1d). The model identified 27 metabolites with VIP > 1.2 that significantly differed between ESKD and NC. Creatinine, p-HPhLA, GCDCA, feruloylputrescine, inosine, phosphocholine, taurocholine, oxalic acid, 5-dodecenoate, and calcitriol had the highest VIP values (Table 2; Fig. 4d).

In the +ve ionization mode, the OPLS-DA model showed R^2^X, R^2^Y, and Q^2^ values of 0.316, 0.991 and 0.966, respectively, indicating good separation between NC and eCKD (Fig. 4e). The permutation testing (100 permutations) confirmed that the model was not overfitted, as the observed Q^2^ value exceeded all permuted values (*p* < 0.01; Supplementary Fig. 1e). The model detected 41 metabolites with VIP > 1.2 that significantly differed between the NC and eCKD groups. Phenylacetylglutamine, ascorbate, lactose, pseudouridine, methionine sulfoxide, DMA, palmitic acid, palmitoyl carnitine, and allantoin had the greatest VIP values (Table 2; Fig. 4f). In comparison between NC and ESKD, the OPLS-DA model revealed the R^2^X, R^2^Y, and Q^2^ values of 0.344, 0.991 and 0.981, respectively (Fig. 4g). The permutation testing (100 permutations) confirmed that the model was not overfitted, as the observed Q^2^ value exceeded all permuted values (*p* < 0.01; Supplementary Fig. 1f). The model identified 54 metabolites with VIP > 1.2 that differed significantly between NC and ESKD. Phenylacetylglutamine, 3-hydroxyanthranilate, TMAO, methylguanidine, glycyl-proline, lysyl-proline, lysophosphatidylcholine (18:1), creatinine, ascorbate, and anthranilic acid had the highest VIP values (Table 2; Fig. 4h).

### Distinct metabolomic variations in end-stage compared to early-stage chronic kidney diseases in untargeted cohort

Pairwise comparisons of serum metabolites from the untargeted discovery cohort showed considerable differences between eCKDand ESKD in both ionization modes. Metabolites were considered significantly altered between CKD stages if FC > 1.5 and FDR < 0.05 (Supplementary Table S6).

**Fig. 5:**
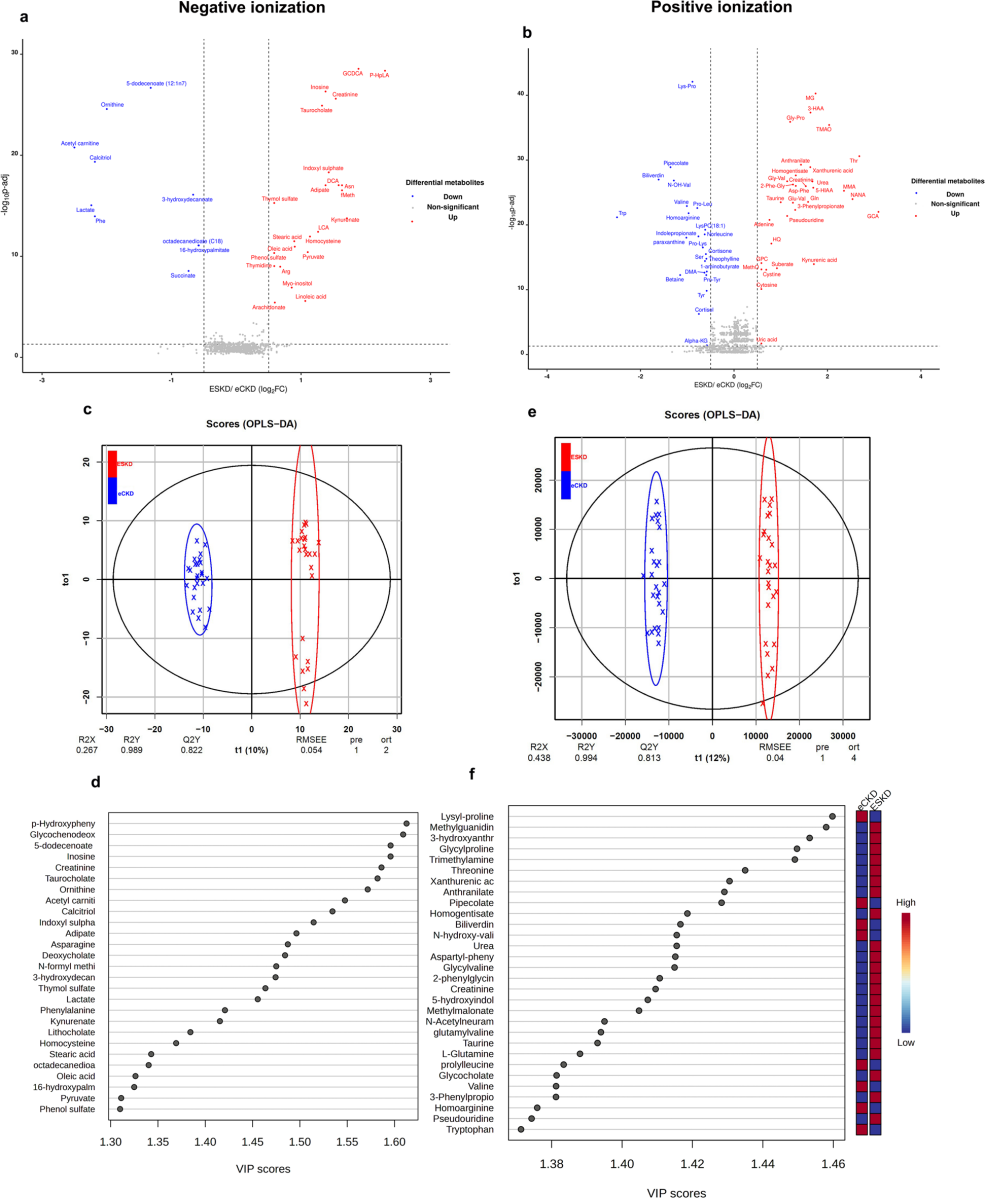
Univariate and multivariate analysis of ESKD versus eCKD patient differences in metabolites for both negative (left panel) and positive (right panel) ionization modes. (**a**,**b**) Volcano plots for the metabolite difference of ESKD versus eCKD. (**a**) In -ve ionization mode, 33 significantly altered metabolites were identified. (**b**) In +ve ionization mode, 54 significantly altered metabolites were identified. For both figures, the y-axis is –log_10_ adjusted p-value and x-axis is log_2_ fold change (ESKD vs. eCKD). Red dots indicate significantly upregulated metabolites, and blue dots indicate significantly downregulated metabolites based on thresholds of log₂ fold change ≥ 0.58 (i.e., fold change ≥ 1.5) and adjusted *p* < 0.05. Statistical testing was done with the Wilcoxon rank-sum test and multiple testing correction using the Benjamini–Hochberg procedure. (**c–f**) OPLS-DA models demonstrating separation between eCKD and ESKD. (**c**) OPLS-DA score plot in -ve mode (R^2^X = 0.267, R^2^Y = 0.989, Q^2^ = 0.822; 100 permutation tests). (**d**) Top-discriminative metabolites in -ve mode with VIP values > 1.2. (**e**) OPLS-DA score plot in +ve mode (R2X = 0.438, R2Y = 0.994, Q^2^ = 0.813, 100 permutations). (**f**) Top 30 discriminative metabolites in +ve mode with VIP scores > 1.2. All statistical analyses were performed using MetaboAnalyst version 6.0. Metabolite selection within multivariate models was executed using variable importance in projection (VIP) scores. Model stability was assessed using 100 permutation tests. R^2^X, R^2^Y reflects the model interpretation rate, and Q^2^ reflects model cross-validated model performance. 2-Phe-Gly: 2-phenyl-glycine, 3-HAA: 3-hydroxy anthranilic acid, Alpha-KG: alpha-ketoglutarate, Arg: arginine, Asn: asparagine, DCA: deoxycholic acid, DMA: dimethylarginine, fMeth: N-formyl methionine, GCA: glycholic acid, GCDCA: glycochenodeoxycholic acid, Gly-Pro: glycyl-proline, GPC: Glycerophosphoryl choline, HQ: hydroquinone, LCA: lithocholic acid, LysPC(18:1): lysophosphatidylcholine (18:1), MethO: methionine sulfoxide, MG: methylguanidine, MMA: methylmalonate, NANA: N-acetyl neuraminic acid, N-OH-Val: N-hydroxy valine, P-HPhLA: p-hydroxyphenyllactic acid, Phe: phenylalanine, Pro-Lys: prolyl-lysine, Pro-Tyr: prolyl-tyrosine, Ser: serine, Thr: threonine, TMAO: Trimethylamine N-oxide, Trp: tryptohan, Tyr: tyrosine.

Univariate analysis in the −ve mode identified 33 metabolites that differed significantly between eCKD and ESKD considerably. Twenty-three of which were upregulated, and 10 were downregulated in ESKD. Inosine, GCDCA, pHPhLA, creatinine, Asn, taurocholate, indoxyl sulphate, deoxycholic acid, adipic acid, and N-formyl methionine were the top upregulated while, 5-dodecenoate (12:1n7), ornithine, acetyl carnitine, calcitriol, and lactate were the top downregulated metabolites in ESKD (Fig. 5a).

In the +ve mode, 54 metabolites significantly differed were between eCKD and ESKD groups. Thirty-one of which were upregulated, and 23 were downregulated in ESKD. Methylguanidine, 3-hydroxyanthranilate, TMAO, threonine, homogentisate, and urea were the top upregulated, whereas lysyl-proline, pipecolate, biliverdin, N-hydroxy-valine, and prolyl-leucine were the most downregulated metabolites in ESKD (Fig. 5b).

Table 3 lists the top 20 significantly different metabolites between eCKD and ESKD (VIP scores > 1.2), showing their chemical formulae, regulation in ESKD, and the associated metabolic pathways.

**Table 3:** Top 20 metabolites showing statistical significance and highest VIP scores differentiating between eCKD and ESKD in negative and positive ionization mode.

Metabolite	mode	m/z	RT (min)	Formula	VIP	Groups	Up/down	FDR	FC	Biochemical pathway
p-hydroxyphenyl lactic Acid	−ve	181.10	3.50	C_9_H_10_O_4_	1.613	ESKD vs. eCKD	Up	4.3E−29	4.91	Amino acid metabolism
Glycochenodeoxycholate	−ve	448.31	9.95	C_26_H_43_NO_5_	1.609	ESKD vs. eCKD	Up	2.78E−29	3.70	Bile acid metabolism
5-dodecenoate (12:1n7)	−ve	197.16	8.70	C_12_H_22_O_2_	1.596	ESKD vs. eCKD	Down	2.13E−27	0.4	Fatty acid metabolism
Inosine	−ve	267.07	1.23	C_10_H_12_N_4_O_5_	1.596	ESKD vs. eCKD	Up	4.89E−27	2.60	Purine metabolism
Creatinine	−ve	112.10	6.90	C_4_H_7_N_3_O	1.586	ESKD vs. eCKD	Up	2.53E−26	2.9	Creatine metabolism
Taurocholate	−ve	514.28	6.67	C_26_H_45_NO_7_S	1.582	ESKD vs. eCKD	Up	1.23E−25	2.50	Bile acid metabolism
Ornithine	−ve	132.09	6.30	C_5_H_12_N_2_O_2_	1.572	ESKD vs. eCKD	Down	2.66E−25	0.25	Urea cycle and amino acid metabolism
Acetyl carnitine	−ve	202.11	2.40	C_9_H_17_NO_4_	1.548	ESKD vs. eCKD	Down	1.71E−21	0.177	Fatty acid oxidation
Calcitriol	−ve	416.63	25.40	C_27_H_44_O_3_	1.534	ESKD vs. eCKD	Down	4.44E−20	0.22	Vitamin D metabolism
Indoxyl sulphate	−ve	212.23	6.20	C_8_H_7_NO_4_S	1.515	ESKD vs. eCKD	Up	4.89E−19	2.69	Tryptophan metabolism, Uremic toxins
Adipic acid	−ve	145.05	6.10	C_6_H_10_O_4_	1.496	ESKD vs. eCKD	Up	9.32E−18	2.6	Tryptophan metabolism
Asparagine	−ve	131.05	3.90	C_4_H_8_N_2_O_3_	1.487	ESKD vs. eCKD	Up	9.32E−18	3.1	Amino acid metabolism
Deoxycholic acid	−ve	391.28	12.10	C_24_H_40_O_3_	1.484	ESKD vs. eCKD	Up	9.17E−18	2.99	Bile acid metabolism
N-formyl methionine	−ve	176.0	6.94	C_6_H_11_NO_3_S	1.475	ESKD vs. eCKD	Up	2.95E−17	3.1	Amino acid metabolism
3-hydroxydecanoate	−ve	187.13	8.40	C_10_H_19_O_3−_	1.474	ESKD vs. eCKD	Down	7.99E−17	0.63	Fatty acid metabolism
Thymol sulfate	−ve	229.05	7.35	C_10_H_14_O_4_S	1.464	ESKD vs. eCKD	Up	5.27E−16	1.50	Phase II metabolism
Lactate	−ve	89.025	1.80	C_3_H_6_O_3_	1.456	ESKD vs. eCKD	Down	8.86E−16	0.21	Glycolysis, Lactate fermentation
Phenylalanine	−ve	164.07	5.20	C_9_H_11_NO_2_	1.420	ESKD vs. eCKD	Down	1.10E−14	0.22	Amino acid metabolism
Kynurenic acid	−ve	188.04	5.90	C_10_H_7_NO_3_	1.415	ESKD vs. eCKD	Up	1.72E−14	3.27	Fatty acid metabolism
Lithocholic acid	−ve	375.27	10.90	C_24_H_40_O_3_	1.384	ESKD vs. eCKD	Up	3.75E−13	2.40	Bile acid metabolism
Lysyl-proline	+ve	355.65	6.58	C_11_H_21_N_3_O_3_	1.460	ESKD vs. eCKD	Down	8.16E−43	0.54	Peptide metabolism
Methylguanidine	+ve	74.071	6.88	C_2_H_7_N_3_	1.458	ESKD vs. eCKD	Up	5.3E−41	3.35	Amino acid metabolism
3-hydroxyanthranilate	+ve	154.049	5.42	C_7_H_7_NO_3_	1.453	ESKD vs. eCKD	Up	4.65E−38	3.11	Tryptophan metabolism
Glycyl-proline	+ve	173.092	6.54	C_7_H_12_N_2_O_3_	1.450	ESKD vs. eCKD	Up	1.26E−36	2.30	Peptide metabolism
Trimethylamine N-oxide	+ve	76.08	0.85	C_3_H_9_NO	1.449	ESKD vs. eCKD	Up	3.78E−36	4.10	Choline metabolism
Threonine	+ve	120.06	0.78	C_4_H_9_NO₃	1.435	ESKD vs. eCKD	Up	2.49E−31	6.41	Amino acid metabolism
Xanthurenic acid	+ve	206.04	2.39	C_10_H_7_NO_4_	1.431	ESKD vs. eCKD	Up	1.22E−29	3.10	Tryptophan metabolism
Anthranilate	+ve	120.04	8.62	C_7_H_7_NO_2_	1.429	ESKD vs. eCKD	Up	5.16E−30	2.7	Tryptophan metabolism
Pipecolate	+ve	130.09	1.19	C_6_H_11_NO_2_	1.428	ESKD vs. eCKD	Down	1.24E−29	0.39	Amino acid metabolism
Homogentisate	+ve	169.05	10.38	C_8_H_8_O_4_	1.419	ESKD vs. eCKD	Up	2.29E−28	2.50	Amino acid metabolism
Biliverdin	+ve	583.25	1.99	C_33_H_34_N_4_O_6_	1.417	ESKD vs. eCKD	Down	1.01E−27	0.33	Porphyrin metabolism
N-hydroxy-valine	+ve	134.08	1.59	C_5_H_11_NO_3_	1.416	ESKD vs. eCKD	Down	1.41E−27	0.41	Amino acid metabolism
Urea	+ve	61.03	2.70	CH₄N₂O	1.416	ESKD vs. eCKD	Up	1.97E−27	3.20	Urea cycle
Aspartyl-phenylalanine	+ve	281.11	5.93	C_13_H_16_N_2_O_5_	1.415	ESKD vs. eCKD	Up	9.54E−27	2.50	Peptide metabolism
Glycyl-valine	+ve	175.11	2.33	C_7_H_14_N_2_O_3_	1.415	ESKD vs. eCKD	Up	1.86E−27	2.2	Peptide metabolism
2-phenylglycine	+ve	152.08	5.50	C_8_H_9_NO_2_	1.411	ESKD vs. eCKD	Up	5.85E−27	2.40	Amino acid metabolism
Creatinine	+ve	114.06	0.62	C_4_H_7_N_3_O	1.410	ESKD vs. eCKD	Up	1.06E−26	2.90	Creatine metabolism
5-hydroxyindoleacetic acid	+ve	192.07	4.11	C_10_H_9_NO_3_	1.407	ESKD vs. eCKD	Up	1.83E−26	3.24	Tryptophan metabolism
Methylmalonate	+ve	117.02	3.10	C_4_H_6_O_4_	1.405	ESKD vs. eCKD	Up	5.44E−26	5.11	Propionate metabolism
N-acetyl neuraminic acid	+ve	310.12	1.20	C_11_H_19_NO_9_	1.395	ESKD vs. eCKD	Up	1.04E−24	5.80	Sialic acid metabolism

Statistical comparisons were performed using the Wilcoxon rank-sum test, with p-values adjusted for multiple testing using the Benjamini-Hochberg FDR. Metabolites with FDR-adjusted *p* < 0.05, FC ≥ 1.5 or ≤ 0.67, and VIP ≥ 1.2 (from OPLS-DA) were considered significant. eCKD: early-stage chronic kidney disease, ESKD: end-stage kidney disease, FC: fold change, FDR: false discovery rate, RT: retention time, VIP: Variance of importance.

In −ve ionization mode, OPLS-DA model demonstrated significant discrimination between eCKD and ESKD with R^2^X, R^2^Y, and Q^2^ values of 0.267, 0.989 and 0.822, respectively (Fig. 5c). The permutation testing (100 permutations) confirmed that the model was not overfitted, as the observed Q^2^ value exceeded all permuted values (*p* < 0.01; Supplementary Fig. 1g). The model identified 27 metabolites with VIP > 1.2. p-HPhLA, GCDCA, 5-dodecenoate (12:1n7), inosine, creatinine, taurocholate, ornithine, acetyl carnitine, calcitriol, IS, adipate, Asn, deoxycholate, N-formyl methionine, 3-hydroxydecanoate, thymol sulfate, lactate, phenylalanine, and kynurenic acid, exhibited the highest VIP values (Table 3; Fig. 5d).

In the +ve ionization mode, the OPLS-DA model had R^2^X, R^2^Y, and Q^2^ values of 0.438, 0.994 and 0.813, respectively, indicating good discrimination between eCKD and ESKD (Fig. 5e). The permutation testing (100 permutations) confirmed that the model was not overfitted, as the observed Q^2^ value exceeded all permuted values (*p* < 0.01; Supplementary Fig. 1h). The model identified 47 metabolites with a VIP > 1.2. Lys-Pro, methylguanidine, 3-hydroxyanthranilate, glycyl-proline, TMAO, threonine, XA, anthranilate, pipecolate, homogentisate, biliverdin, N-hydroxy-valine, urea, aspartyl-phenylalanine, glycyl-valine, 2-phenylglycine, and creatinine had the highest VIP values (Table 3; Fig. 5f).

### Enrichment and pathway analysis

**Fig. 6:**
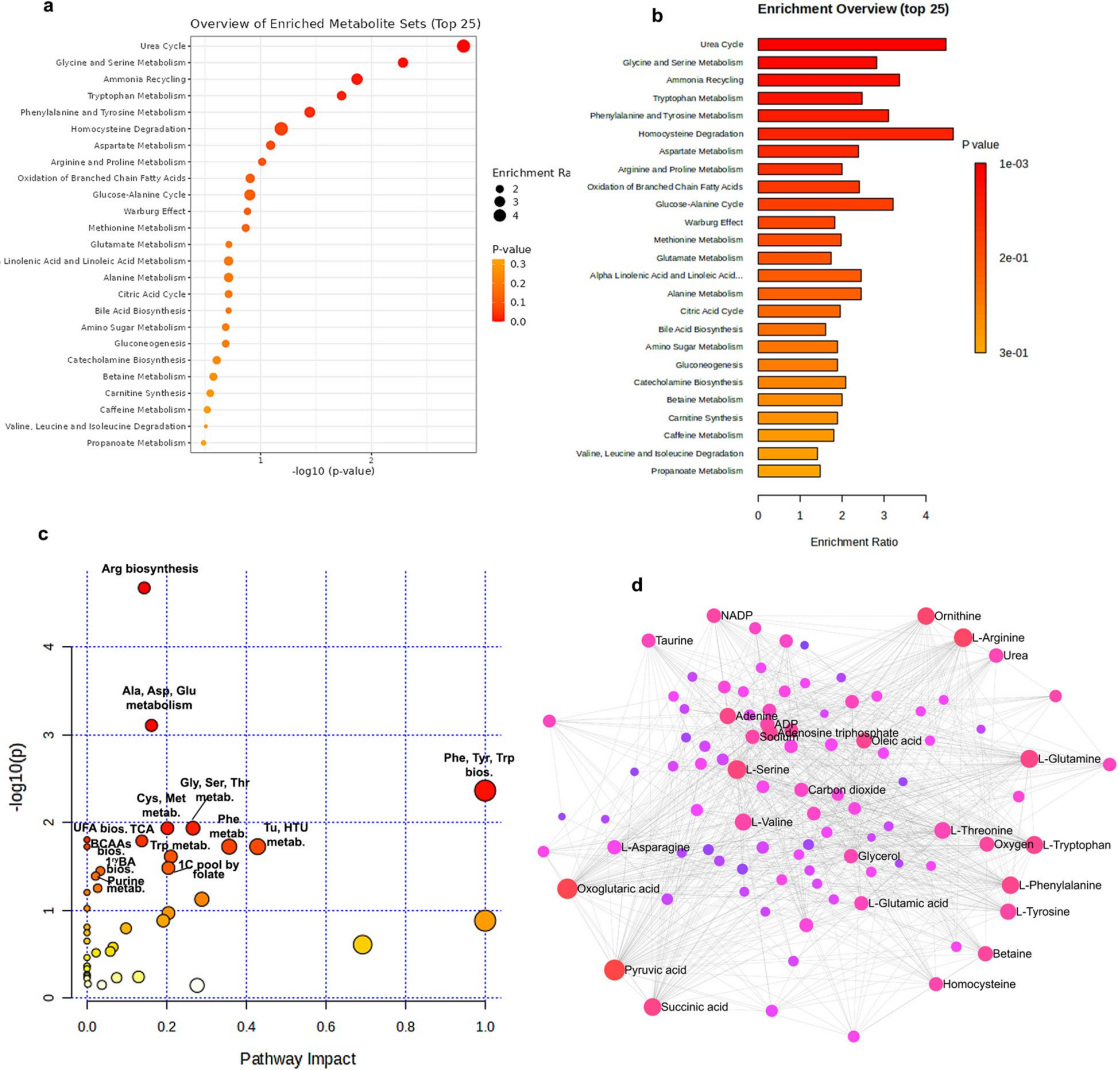
Pathway and network analysis of metabolites differing between eCKD and ESKD. (**a**) Enrichment analysis using the Small Molecule Pathway Database (SMPDB) database. Each spot represents a metabolic pathway identified as enriched in the comparison. Pathways are graphed on the y-axis and ranked by their significance (–log_10_ of the *p*-values on x-axis), and dot size represents the enrichment strength. The color gradient represents diminishing significance, from red to yellow. (**b**) Bar plot of results from the over-representation analysis against the SMPDB based on fold enrichment and statistical significance. (**c**) Pathway impact analysis integrating enrichment and topology analysis. Every point represents some metabolic pathway. Dot size indicates pathway impact, integrating pathway centrality and enrichment scores and color intensity indicates statistical significance, with darker red implying lower adjusted *p*-values. Larger and darker spots indicate pathways of greater biological importance. This analysis identifies the most impactful pathways contributing to metabolic distinction between CKD stages based on both their relative importance and statistical confidence. (**d**) Metabolite–metabolite interaction network of significantly differentiated metabolites. Nodes represent metabolites, and edges represent enzymes^20^. Node color indicates betweenness centrality, compounds exhibiting elevated betweenness centrality are denoted in red, then pink and blue. Node size reflects degree of connectivity, representing the number of interactions it has with other nodes. Topology-based methods of MetaboAnalyst 6.0, along with enrichment and network analysis tools, were used to perform pathway enrichment and metabolite-metabolite interaction analysis to evaluate biological and functional relevance. The statistical significance was evaluated using an adjusted *p*-value of < 0.05.

A total of 74 metabolites significantly differed between eCKD and ESKD patients in univariate and multivariate analyses. Kynurenic acid and creatinine were detected in both ionization modes, hence only 72 distinct metabolites were used in enrichment and pathway analyses. Enrichment analysis (Fig. 6a, b) showed significant disturbances in multiple metabolic pathways, including urea cycle dysfunction, and amino acid metabolism disorder (glycine, serine, tryptophan, and phenylalanine and tyrosine) pathways. Urea cycle was the most enriched, indicating acute renal disease-related nitrogen regulation defects. Ammonia recycling enrichment, homocysteine degradation, aspartate metabolism, and Arg and proline metabolism demonstrate metabolic reprogramming linked with deteriorating renal function. The higher enrichment ratios and significant *p*-values in the dot and bar plots show that urea cycle and amino acid metabolism abnormalities are critical to ESKD pathogenesis.

Pathway analysis showed changes in several metabolic pathways, including Arg biosynthesis; alanine, aspartate, and glutamate metabolism; of phenylalanine, tyrosine, and tryptophan biosynthesis; cysteine and methionine metabolism; glycine, serine, and threonine metabolism; the biosynthesis of unsaturated fatty acids; the citric acid cycle; valine, leucine, and isoleucine biosynthesis; phenylalanine metabolism; taurine and hypotaurine metabolism; tryptophan metabolism; the one carbon pool by folate; the primary bile acid biosynthesis; and purine metabolism (Fig. 6c). These findings underscore that dysregulation in several amino acid, nucleotide, and lipid metabolic pathways as potential drivers or disease biomarkers of disease severity.

Pyruvic acid, oxoglutaric acid, serine, Arg, glutamate, tryptophan, succinic acid, phenylalanine, ornithine, valine, threonine, adenine, tyrosine, adenosine triphosphate, oleic acid, and betaine showed maximum interactions with other metabolites, suggesting the optimal functional relationship with other metabolites in the network (Fig. 6d and Supplementary Table S7). Kyoto Encyclopedia of Genes and Genomes (KEGG) functional pathways that are significantly enriched among network metabolites^20^ are shown in Supplementary Table S8.

### Metabolomic correlations with eGFR in CKD patients

**Fig. 7:**
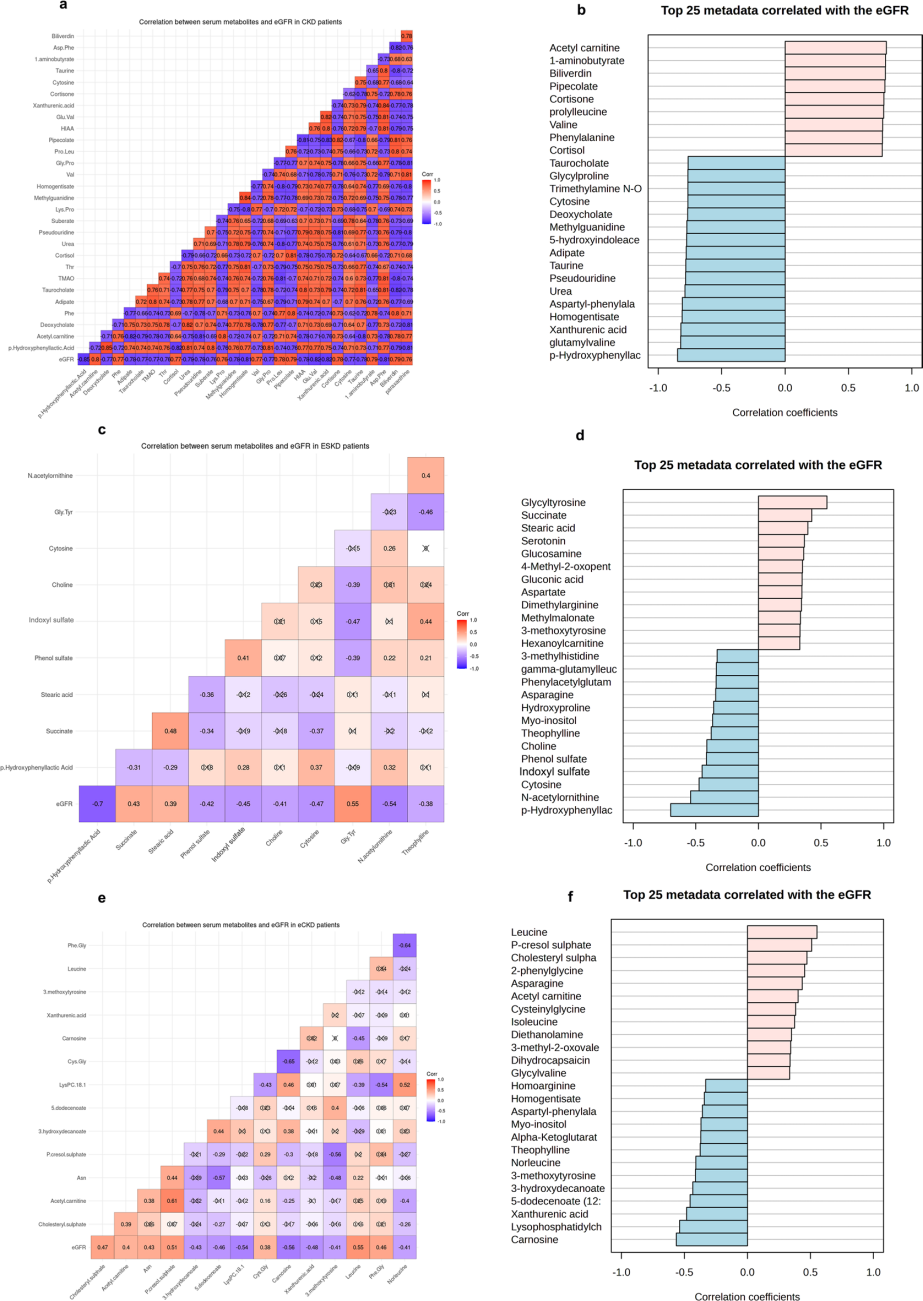
Correlation of serum metabolites with estimated glomerular filtration rate (eGFR) (**a**) Correlation matrix of the top 30 significant correlations of serum metabolites with eGFR in eCKD and ESKD patients (*n* = 50). (**b**) Bar plot of the correlation coefficients of the top 25 metabolites that were significantly correlated with eGFR in eCKD and ESKD. The x-axis indicates the correlation coefficients (ρ) (range: -1.0 to 1.0), where positive values (red) indicate direct and negative values (blue) indicate inverse correlations. (**c**) Correlation matrix of significant correlations between serum metabolites and eGFR in ESKD patients (*n* = 25). (**d**) Bar graph of the top 25 metabolites that were significantly correlated with eGFR in ESKD patients. (**e**) Correlation matrix of significant correlations between serum metabolites and eGFR in eCKD patients (*n* = 25). (**f**) Bar plot of top 25 metabolites significantly correlated with eGFR in eCKD patients. Spearman’s rank correlation was used to assess correlations between differential metabolites and eGFR, and statistical significance was considered at *p* < 0.05.

Spearman rank correlations between serum metabolites intensities and eGFR in CKD patients are shown in Supplementary Table S9. The strongest inverse correlations with eGFR included p-HPhLA (*r*= -0.847, *p* < 0.001), glutamyl-valine (*r*= -0.824, *p* < 0.0001), and XA (*r*= -0.822, *p* < 0.001), indicating accumulation of these metabolites with worsening kidney function. On the other hand, acetyl carnitine (*r* = 0.798, *p* < 0.001), 1-aminobutyrate (*r* = 0.792, *p* < 0.001), and biliverdin (*r* = 0.790, *p* < 0.001) had the strongest positive correlations with eGFR. Biomarkers or renal impairment, urea (*r*= -0.790, *p* < 0.001) and creatinine (*r*= -0.733, *p* < 0.001) showed the expected negative correlations. Notably, gut-derived uremic toxins such as IS (*r*= -0.749, *p* < 0.001) and TMAO (*r*= -0.767, *p* < 0.001) were inversely correlated with eGFR, while markers of energy metabolism such as lactate (*r* = 0.74, *p* < 0.001) and succinate (*r* = 0.725, *p* < 0.001) were positively correlated with eGFR levels (Fig. 7a, b).

Correlation analysis with eGFR in patients with ESKD and eCKD clarified stage-specific metabolic associations. For ESKD patients, the strongest negative correlation was that between p-HPhLA and eGFR (*r* = -0.700, *p* < 0.001), a marker of its accumulation with advanced kidney dysfunction. N-acetylornithine (*r*=-0.542, *p* < 0.01), cytosine (*r*=-0.474, *p* < 0.05), and vitamin B2 (*r*=-0.449, *p* < 0.05) were the other strong negative correlations. Both glycyl-tyrosine (*r* = 0.547, *p* < 0.01) and succinate (*r* = 0.426, *p* < 0.05) were positively correlated (Fig. 7c, d). In eCKD patients, carnosine showed the strongest negative correlation with eGFR (*r*= -0.565, *p* < 0.01), followed by lysophosphatidylcholine (18:1) (*r*= -0.538, *p* < 0.04) and XA (*r*= -0.482, *p* < 0.05). Leucine (*r* = 0.552, *p* < 0.01), PCS (*r* = 0.510, *p* < 0.01), and cholesteryl sulfate (*r* = 0.472, *p* < 0.05) were positively correlated. These findings suggest amino acid metabolism and lipid-related metabolites are altered in early stages of CKD (Fig. 7e, f).

### Association between serum metabolites and CKD stage in the validation cohort

Five significantly different metabolites (p-HPhLA, IS, TMAO, GCDCA, and XA) between eCKD and ESKD identified using untargeted metabolomics were quantitatively confirmed using analytical standards and compared to creatinine in an independent validation cohort. All metabolites exhibited excellent linearity (R^2^ ≥ 0.99) within their validated ranges (Supplementary Table S2).

**Fig. 8:**
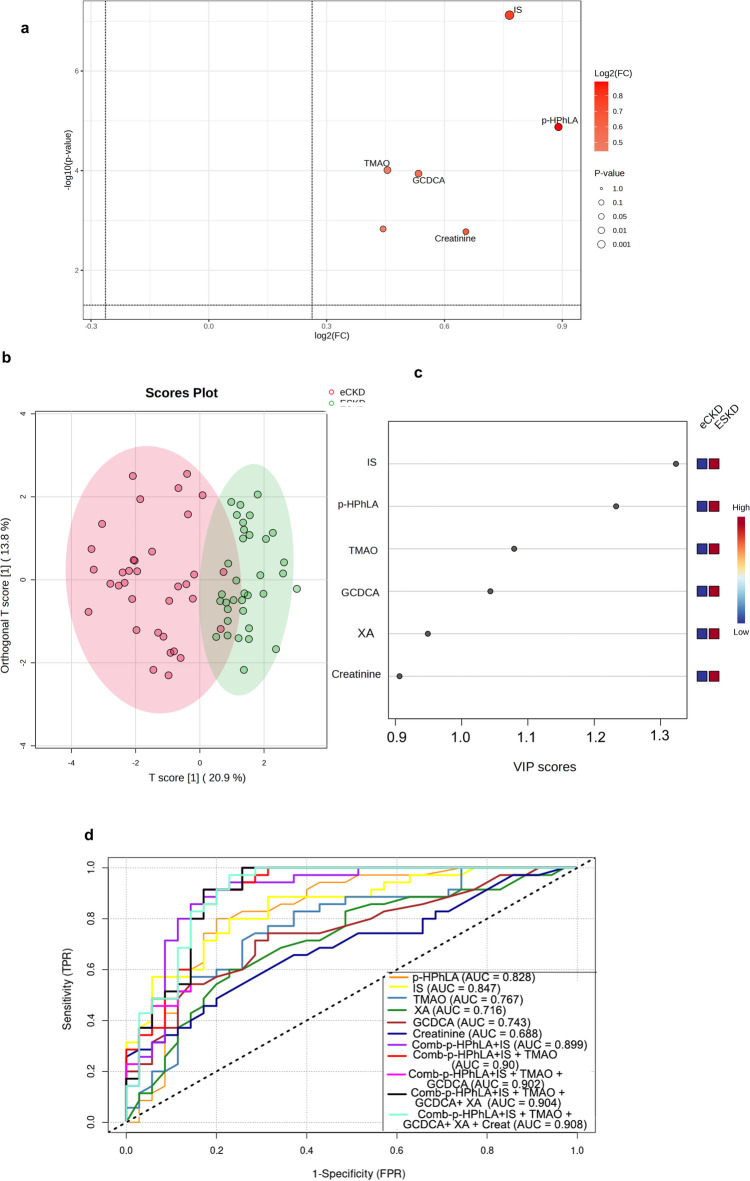
Differential serum metabolites between eCKD and ESKD in the validation cohort. (**a**) Volcano plots for differentiation between eCKD and ESKD. (**b**) OPLS-DA score plot with clear segregation between eCKD and ESKD (R^2^X = 0.209, R^2^Y = 0.728, Q^2^ = 0.648, internally validated through 100 permutation tests) using targeted metabolites (p-HPhLA, IS, TMAO, GCDCA, XA and creatinine). (**c**) VIP scores of p-HPhLA, IS, TMAO, GCDCA, XA and creatinine for discrimination ESKD from eCKD. (**d**) Receiver operating characteristic (ROC) analysis evaluating the discriminatory performance of six metabolites for distinguishing ESKD from eCKD. The analysis demonstrates that IS (AUC = 0.847) and p-HPhLA (AUC = 0.828) exhibited the highest discriminative performance, followed by TMAO (AUC = 0.767), GCDCA (AUC = 0.743), and XA (AUC = 0.716). Creatinine (AUC = 0.688) showed the lowest discriminatory performance. The diagonal dashed line represents the line of no discrimination (AUC = 0.5). AUC: area under the curve, eCKD: early-stage chronic kidney disease, ESKD: end-stage kidney disease, IS: indoxyl sulphate, GCDCA: glycochenodeoxycholate, p-HPhLA: p-hydroxyphenyllactic acid, TMAO: trimethylamine-N-oxide, XA: xanthurenic acid.

Univariate analysis showed significant differences in all metabolites between eCKD and ESKD (FC ≥ 1.5 and FDR < 0.05) (Fig. 8a). OPLS-DA model distinguished ESKD from eCKD for targeted differential metabolites. R^2^X, R^2^Y, and Q^2^ values of 0.209, 0.728, and 0.648 indicated strong differentiation between eCKD and ESKD (Fig. 8b). IS and p-HPhLA had the highest VIP, followed by TMAO, GCDCA, XA, and creatinine (Fig. 8c). The ROC curve was used to evaluate whether these metabolites discriminate between eCKD and ESKD. IS, p-HPhLA, TMAO, GCDCA, and XA discriminated ESKD from eCKD better than creatinine (AUC > 0.7). Table 4 lists metabolites and AUCs. IS and p-HPhLA well discriminated between CKD stages (AUC > 0.80). Additionally, a model that integrated the five metabolites with creatinine had the highest discriminative performance (AUC = 0.908) (Table 4; Fig. 8d).

**Table 4:** Discriminatory performance of selected metabolites in distinguishing eCKD and ESKD based on ROC analysis.

Metabolites	AUC	95% CI	SE
IS	0.847	0.757–0.936	0.0456
p-HPhLA	0.828	0.726–0.929	0.0523
TMAO	0.767	0.654–0.880	0.0577
GCDCA	0.743	0.628–0.859	0.059
XA	0.716	0.594–0.837	0.0621
Creatinine	0.688	0.564–0.812	0.0634
Combined-IS + p-HPLA	0.899	0.823–0.975	0.0390
Combined-IS + p-HPLA + TMAO	0.900	0.825–0.976	0.0390
Combined-IS + p-HPLA + TMAO + GCDCA	0.902	0.827–0.977	0.0380
Combined-IS + p-HPLA + TMAO + GCDCA + XA	0.904	0.830–0.978	0.0378
Combined-IS + p-HPLA + TMAO + GCDCA + Creatinine	0.908	0.835–0.980	0.0370

AUC: area under the curve, CI: confidence interval, IS: indoxyl sulphate, GCDCA: glycochenodeoxycholate, p-HPhLA: p-hydroxyphenyllactic acid, SE: standard error, XA: xanthurenic acid.

## Discussion

CKD is becoming an increasing health problem in Egypt, with an increasing number of patients presenting without clearly identifiable etiology^21,22^. In 2020, 13–27% of CKD in Egypt had unclear origins, a similar trend observed in other regions of the world like India and Central America^23,24^. At the same time, standard clinical tests, like serum creatinine and eGFR, reflect the loss of kidney function, and often only change after significant kidney damage has occurred. Therefore, these tests don’t provide much information about the biological processes causing the disease or the early metabolic problems that happen before noticeable kidney dysfunction^25,26^.

In this context, metabolomics offers a complementary approach. It can identify systemic biochemical changes that might appear earlier in the disease and could help generate ideas about the underlying pathways and potential ways to assess risk, rather than replacing standard functional markers^27^.

Current metabolomics studies demonstrated critical metabolic alterations in CKD pathogenesis^28,29^. To best of our knowledge, this is the first untargeted metabolomic study in an Egyptian cohort to characterize CKD-associated metabolic alterations and to discriminate between eCKD and ESKD. The identified metabolic signatures reflect dysregulation in amino acid, energy, and lipid metabolism systemically. While our findings are consistent with global CKD metabolomics literature, they also highlight the potential influence of Egyptian genetic, nutritional, and environmental factors.

Significant metabolic alterations were also noted between eCKD and ESKD. Pathway analysis found disturbances in Arg biosynthesis, the citric acid cycle, amino acid metabolism, and bile acid synthesis, which reflected energy, inflammation, membrane function, and gut microbiota imbalances. Certain metabolites, e.g., hArg, lipid derivatives [e.g., lysophosphatidylcholine (18:1)], gut-derived metabolites (e.g., IPA, and TMAO), were not mapped to SMPDB pathways. Such findings may indicate potential novel mechanisms for exploration.

IPA, hArg, γ-glutamyl-phenylalanine, and 2-hydroxystearate were upregulated in eCKD compared to NC, indicating responses to oxidative stress, vascular dysfunction, and inflammation^30,31^. These protective mechanisms failed as CKD proceeded to ESKD. IPA had biphasic trend increased in eCKD and reduced in ESKD^32,33^. In eCKD, IPA improves mitochondrial activity, intestinal barrier function, inflammation, and renoprotection^34^. Its reduction in ESKD may suggest gut dysbiosis, uremic toxin accumulation, and metabolic abnormalities, indicating its dual significance as an early biomarker and protective mechanism^32^.

hArg also increased in eCKD but decreased in ESKD, indicating initial vascular-protective mechanisms that later fail. hArg deficiency increases cardiovascular risk and CKD progression because nitric oxide metabolism mediates its role in endothelial function^35,36^. These findings confirm its cardiorenal biomarker role. Upregulation of Arg, citrulline, urea, arginosuccinate, DMA, and allantoin in eCKD and ESKD indicates early urea cycle and nitrogen metabolism impairment. This may be of particular interest in Egypt, where high protein intake and dehydration promote nitrogen burden and CKD development. Tubulointerstitial damage may also be more common than diabetes and hypertension in tropical CKD^37^.

We observed significant alterations in amino acid metabolism across CKD stages. CKD is characterized by chronic alteration in amino acid metabolism, especially tryptophan pathways^38^. Changes in plasma amino acid levels have been reported early in CKD and are more pronounced in advanced CKD stages. Renal dysfunction alters serine, valine, leucine, and citrulline. Plasma citrulline levels rose when eGFR dropped below 45 mL/min/1.73m^2^^39^. These findings highlight the intricate link between renal function and amino acid metabolic profiles in ESKD.

Arg increased progressively from eCKD to ESKD compared to NC. DMA increased in eCKD but decreased in ESKD. This fluctuating pattern aligns with the role of methylarginines in endothelial dysfunction via nitric oxide inhibition^40–42^. Kidney damage was linked to DMA accumulation^43^. DMA levels may drop from eCKD to ESKD due to metabolic adjustments including increased DMA dimethylaminohydrolase activity or slower protein turnover due to muscle atrophy and malnutrition. Although methylarginines cannot distinguish chronic from acute kidney injury, they can indicate disease severity^44^. In Egypt, where cardiovascular disease and hypertension dominate CKD patients, arginine-nitric oxide pathway change may be more clinically significant^24,40^. Research into targeted therapy, dimethylaminohydrolase regulation, or Arg supplementation to reduce vascular problems in ESKD is needed due to progressive Arg and DMA alterations.

There were major changes in tryptophan-kynurenine pathway. ESKD had higher key intermediates (kynurenic acid, XA, 3-hydroxyanthranilate, anthranilic acid) and lower tryptophan than eCKD. These observed modifications align with increased pathway flux, a characteristic of advanced CKD, which is associated with oxidative stress and chronic inflammation^38,45^. Furthermore, prior research has indicated a progressive activation of this pathway across the various stages of CKD^28,38,46^.

Conversely, elevated kynurenine-pathway metabolites might also indicate diminished renal clearance and the subsequent accumulation of metabolites due to impaired kidney function, in conjunction with increased pathway activation^47^. Although toxic kynurenine metabolites have been linked to endothelial dysfunction, oxidative stress, and disease severity^48^, Indoleamine 2,3-dioxygenase (IDO) activation does not consistently exhibit pathogenic effects. In some situations, IDO-mediated tryptophan catabolism could serve as a counter-regulatory mechanism, thereby inhibiting immune activation and promoting tolerance^49^.Consequently, the kynurenine alterations observed in our investigation likely arise from a combination of impaired excretion, pathway activation, and host regulatory responses.

Moreover, ESKD significantly upregulated the uremic toxins IS, 5-hydroxy indole acetate, phenol sulfate, and methylguanidine. This supports prior evidence that uremic toxin accumulation in ESKD induces oxidative stress, inflammation, and CKD development via the tryptophan-kynurenine pathway^28,50,51^. IS is critical for endothelial dysfunction, cardiovascular disease, apoptosis, mitochondrial damage, and renal fibrosis^50,52–54^. This interaction shows that kynurenine pathway activation and uremic toxin accumulation may form a feedback loop that promotes eCKD to ESKD and reveals potential therapeutic targets.

Beyond tryptophan metabolism, our results indicate unprecedented alterations in gut microbiome-derived metabolites, including TMAO, phenylacetylglutamine, PCS, IS, hippurate, and kynurenic acid, emphasizing the importance of gut dysbiosis in CKD pathogenesis Interestingly, TMAO was upregulated in eCKD and ESKD compared to NC and further increased in ESKD compared to eCKD. These findings support earlier research reporting associations between elevated TMAO levels and CKD development and progression^55–57^. Elevated TMAO levels were associated with endothelial dysfunction, vascular inflammation, and cardiovascular events, all of which are CKD consequences^58^. The higher levels of TMAO in advanced stages may reflect gut dysbiosis and impaired renal clearance and have been proposed as a biomarker associated with CKD severity and a suitable therapeutic target.

In addition, gut microbiota-derived uremic toxins originating from tyrosine and tryptophan metabolism, PCS and IS, rose considerably in CKD^59,60^. We demonstrated that IS was gradually upregulated, significantly higher in ESKD than eCKD and NC, while PCS increased in eCKD and ESKD compared to NC. Both of these toxins have been implicated in oxidative damage, endothelial dysfunction, and kidney fibrosis, thereby accelerating renal function loss and cardiovascular complications in CKD patients^60–62^. Hippuric acid, a metabolite of phenylalanine and polyphenol, was also significantly upregulated in CKD patients. High hippuric acid levels indicate decreased tubular secretion and renal function^63,64^. These findings are in agreement with previous research that demonstrated how gut microbiota-derived metabolites affect inflammation and oxidative stress which proves the relevance of gut-kidney metabolic interactions with CKD development^65^. Thus, targeting gut microbiota might be a potential therapeutic strategy to prevent the buildup of uremic toxins in the body and may warrant further investigation in future mechanistic studies^66^.

In the present study, ESKD was associated with remodeling of lipid and fatty acid metabolism, including downregulation lysophosphatidylcholine, acetylcarnitine, 3-hydroxydecanoate, and 5-dodecenoate, and upregulation of stearic acid. The reduction in lysophosphatidylcholine, key regulators of membrane integrity and anti-inflammatory signaling, in ESKD^67^, suggests impaired lipid metabolism contributing to endothelial dysfunction, oxidative stress, and inflammation, hallmarks of advanced CKD stage. The research supports earlier studies showing that lecithin cholesterol acyl transferase activity decreases in CKD patient^68^ and these changes link to cardiovascular conditions and increased inflammation in patients with ESKD^67,69,70^.

Downregulation of acetylcarnitine, which is a primary source of mitochondrial energy^71^, indicates possible mitochondrial damage in advanced kidney disease. Previous investigations reported inconsistent results based on CKD stage and patient heterogeneity^72,73^. Medium-chain fatty acid downregulation, along with acetylcarnitine decline, and stearic acid increase indicate fatty acid oxidation impairment and metabolism disruption. The findings support individual metabolic assessments of ESKD patients and suggest treatments for mitochondrial dysfunction and carnitine metabolism abnormalities in ESKD.

Energy metabolism variations in ESKD suggest substantial metabolic dysregulation in advanced kidney disease, while lipid metabolism modifications reflect broader systematic disturbances. We observed a downregulation of lactate along with an upregulation of pyruvate and methylmalonate in ESKD compared with eCKD. These metabolites’ changes may reflect disturbances in intermediary and mitochondrial-related energy pathways. Lactate accumulation has been linked to reduced clearance and impaired gluconeogenesis in CKD. However, clinical treatments can maintain acid-base balance by affecting lactate levels in the body, which might explain the differences seen in various studies^74–76^. Also, controversies in study results could be due to variations in the patient groups, the treatments used, and their overall metabolic state. Increased methylmalonate has been associated with changes in propionate metabolism and a lack of vitamin B12, possibly indicating metabolic stress^77^.

Lactate downregulation and purine metabolism alterations in ESKD indicate energy and nucleotide pathway disparities in late kidney disease. We found that ESKD upregulated inosine, adenine, and pseudouridine compared to eCKD. This confirms increased oxidative stress and inflammation as CKD advances^78,79^. These purines increase ROS generation and oxidative damage as renal clearance declines. High adenine levels were linked to renal hypoxia, a factor in CKD advancement^80^. Pseudouridine, a nucleoside modification found in RNA turnover, is a reliable CKD severity biomarker which correlates with eGFR and reveals inadequate renal reabsorption. It estimates eGFR better than cystatin C or creatinine^81^. We observed that pseudouridine, creatinine, and methylguanidine correlated negatively with eGFR in CKD patients, indicating lower renal excretory capacity^82^.

The disruptions in energy, purine and bile acids metabolism indicate systematic failure in ESKD patients. Serum GCDCA, taurocholate, and deoxycholate were upregulated in ESKD patients, confirming prior findings of higher plasma conjugated bile acids in severe renal failure^83,84^. Systemic inflammation, gut dysbiosis, and hormonal abnormalities can cause these disturbances. The kidneys function to control liver-intestine bile acid circulation through their ability to process uremic toxins. The abnormal bile acid patterns stem from changes in gut microbiota which create ongoing inflammation that damages metabolic processes^83,85^. Gai et al.^86^ proposed that CKD progression may be indicated by higher plasma bile acid. This shows how renal and hepatic impairment interact in ESKD.

Metabolite-metabolite network interactions showed that urea cycle and amino acid metabolism disruptions in ESKD affect downstream production of uremic toxins and secondary metabolite intermediates^20^, confirming impaired nitrogen metabolism and gut dysbiosis in advanced CKD^32^. The uremic phenotype and renal patients’ epigenetic landscape are heavily regulated by micronutrient consumption, gut flora, inflammatory state, oxidative stress, and lifestyle choices. All these variables help CKD preserve methylome and epigenetic control. The rise in homocysteine and fall in betaine supports the significance of methyl donors and vitamin B in CKD development^87^.

Our study identified metabolites that had significant correlation with eGFR in CKD, providing insights into metabolic alterations associated with disease severity. Strong inverse correlations with p-HPhLA and XA indicate abnormal tyrosine and tryptophan metabolism^88^. The negative correlation of TMAO and IS with eGFR confirms previous reports implicating the role gut-microbiota derived metabolites in CKD severity^55^. p-HPhLA was the most inversely correlated with eGFR in ESKD, suggesting its accumulation in advanced kidney dysfunction. In eCKD, negative correlations were observed for carnosine and lysophosphatidylcholine (18:1) with eGFR while positive correlations were observed for leucine and PCS with eGFR, suggesting early metabolic alterations in amino acid, lipid and gut-derived metabolite pathways. Importantly, these correlations are descriptive only, as many metabolites are grouped together by common biochemical pathways; thus, they may be intercorrelated and should not be interpreted as independent drivers of disease. Further studies incorporating multivariate and longitudinal analyses will be required to assess the independent contributions of these metabolites.

Five metabolites that significantly differentiated ESKD from eCKD in the untargeted discovery cohort were quantified in an independent validation cohort using internal standards. OPLS-DA showed that IS, p-HPhLA, TMAO, GCDCA, XA, and creatinine contributed to the discrimination between eCKD and ESKD. We found that IS (AUC = 0.847, 95% CI = 0.757–0.936) had superior discriminatory performance between CKD stages compared with creatinine (AUC = 0.688, *p* = 95% CI 0.564–0.812), consistent with recent metabolomics studies. Dahabiyeh et al.^28^ demonstrated that IS was upregulated across CKD stages. Another serum metabolomics investigation by Gu et al.^89^ demonstrated that numerous uremic toxins, including IS, could distinguish CKD patients from healthy controls with good accuracy. Vanholder et al.^61^ have also demonstrated the association of IS with nephrotoxic and cardiovascular effects. Concentrations of IS in the uremic range also induced deleterious effects in many experimental systems, pointing to the importance of this metabolite as a pathogenic factor and a potential marker for advanced CKD. Moreover, Lin et al.^62^ reported the significant association between IS and cardiovascular events, and all-cause mortality in patients with chronic renal failure. Its accumulation in advanced disease stage is thought to reflect decreased renal clearance and increased gut microbial production. p-HPhLA also exhibited strong discriminatory performance between CKD stages, suggesting its involvement in metabolic alterations characteristic of advanced CKD^28^.

The modest accuracy of creatinine (AUC = 0.688, 95% CI 0.564–0.812) observed in our study highlights the inadequacy of classical biomarkers in CKD staging. staging. All the identified metabolites showed better ability to distinguish between eCKD and ESKD than creatinine. This suggests that creatinine alone might not fully capture the metabolic changes seen in different stages of CKD. A recent study found the urgent need for novel CKD biomarkers that are more specific and sensitive^89^. We found that IS, p-HPhLA, TMAO, GCDCA, and XA with creatinine had the best discriminative performance (AUC = 0.908, 95% CI 0.835–0.98) and may indicate disease severity. This suggests that this multi-metabolite panel forms a robust tool for CKD stage discrimination, supporting the utility of multi-biomarker techniques, which have outperformed single markers in metabolomics investigations.

The relatively small sample size, single-center cohort, and incomplete information on certain clinical variables such as diabetes, hypertension, nutritional status, and hemodialysis exposure limit this study. However, the degree of missing data for these clinical variables was moderate (< 20% by group) and is therefore unlikely to represent substantial missingness. Moreover, the available data did not show significant differences in the prevalence of diabetes and hypertension between CKD stages that would account for the observed metabolomic differences. Potential confounding effects was further minimized through strict inclusion and exclusion criteria, including excluding participants receiving medications known to significantly alter metabolic profiles, as well as they were matched for age, sex, and BMI across groups. Nevertheless, the findings should be interpreted as stage-associated metabolic differences and warrant confirmation in larger, longitudinal studies with more complete clinical characterization. Furthermore, while the use of 0.1% formic acid in the mobile phase enhances chromatographic stability, it could have diminished the negative-mode ionization efficiency for specific bile and organic acids, possibly leading to an underrepresentation of their relative abundance.

In conclusion, the current study provides the first untargeted metabolomics investigation to identify CKD-specific alterations in amino acid, lipid, energy, purine, and bile acid metabolism, together with the gut microbiome-kidney interaction in Egyptian patients. Stage-specific signatures identify the elevation of the potential metabolic biomarkers, IPA and hArg, in eCKD while reduction in ESKD together with uremic toxin and conjugated bile acids increase with CKD severity. These metabolites indicate alterations in tryptophan-kynurenine metabolism, the arginine-nitric oxide pathway, and the gut-kidney interaction, implicated in the pathogenesis of vascular dysfunction, oxidative stress, and inflammation in CKD. Certain metabolites, glutamyl-valine, p-HPhLA, IS and XA showed robust correlations with eGFR, in stage-specific manner. Targeted metabolomics analyses of the validation cohort showed that selected metabolites, particularly IS and p-HPhLA, demonstrate superior performance to discriminate between eCKD and ESKD patients than traditional creatinine. These findings emphasize the role of metabolomics signatures to indicate CKD stage-specific alterations and the potential value of metabolomics to discover novel biomarkers. Future studies should include comprehensive assessment of larger and diverse cohorts encompassing consideration of lifestyle and/or diet and employ multi-omics approach to define the clinical implications and CKD management value of these and other CKD-specific alterations.

## Supplementary Information

Below is the link to the electronic supplementary material.

Supplementary Material 1

Supplementary Material 2

Supplementary Material 3

Supplementary Material 4

Supplementary Material 5

Supplementary Material 6

Supplementary Material 7

Supplementary Material 8

Supplementary Material 9

Supplementary Material 10

## Data Availability

The data generated and/or analyzed during the current study are available from the corresponding author upon reasonable request.

## References

[1] Outcomes, K. D. I. G. Chap. 1: Definition and classification of CKD. *Kidney Int. Suppl.* **3**(1),19–62. 10.1038/kisup.2012.64 (2011). 10.1038/kisup.2012.64 PMC4089693 25018975

[2] Zhang, S. et al. Global, regional, and national burden of kidney dysfunction from 1990 to 2019: a systematic analysis from the global burden of disease study 2019. *BMC Public. Health*. **23** (1), 1218. 10.1186/s12889-023-16130-8 (2023). 37353821 10.1186/s12889-023-16130-8 PMC10288715

[3] Levey, A. S. & Coresh, J. Chronic kidney disease. *lancet* **379** (9811), 165–180 (2012). 21840587 10.1016/S0140-6736(11)60178-5

[4] Foreman, K. J. et al. Forecasting life expectancy, years of life lost, and all-cause and cause-specific mortality for 250 causes of death: reference and alternative scenarios for 2016–40 for 195 countries and territories. *Lancet* **392** (10159), 2052–2090. 10.1016/S0140-6736(18)31694-5 (2018). 30340847 10.1016/S0140-6736(18)31694-5 PMC6227505

[5] Stevens, P. E. et al. KDIGO 2024 Clinical Practice Guideline for the Evaluation and Management of Chronic Kidney Disease. *Kidney Int.* **105** (4s), S117–s314. 10.1016/j.kint.2023.10.018 (2024). 38490803 10.1016/j.kint.2023.10.018

[6] Hocher, B. & Adamski, J. Metabolomics for clinical use and research in chronic kidney disease. *Nat. Rev. Nephrol.* **13** (5), 269–284. 10.1038/nrneph.2017.30 (2017). 28262773 10.1038/nrneph.2017.30

[7] Vaidya, S. R. & Aeddula, N. R. *Chronic kidney disease, in StatPearls [Internet]* (StatPearls Publishing, 2022).

[8] Koh, L. F. A., Khatri, P., Diseases, K. & Quah, S. R. in International Encyclopedia of Public Health (Third Edition), Academic: Oxford. 446–451. (2025).

[9] Wen, H. et al. Association of oxidative balance score with chronic kidney disease: NHANES 1999–2018. *Front. Endocrinol.* **15** (2024). 10.3389/fendo.2024.1396465 (2024). 10.3389/fendo.2024.1396465 PMC11198875 38919480

[10] Aderinto, N. et al. Genomic insights into renal diseases: advancements and implications. *Egypt. J. Intern. Med.* **36** (1), 73. 10.1186/s43162-024-00341-5 (2024).

[11] El-Rashidy, M. A. An efficient methodology for discovering both of gene-environment interactions and gene-gene interactions causing genetic diseases. *Egypt. Inf. J.* **21** (1), 13–22. 10.1016/j.eij.2019.10.001 (2020).

[12] Chen, D. Q. et al. Identification of serum metabolites associating with chronic kidney disease progression and anti-fibrotic effect of 5-methoxytryptophan. *Nat. Commun.* **10** (1), 1476. 10.1038/s41467-019-09329-0 (2019). 30931940 10.1038/s41467-019-09329-0 PMC6443780

[13] Davies, R. The metabolomic quest for a biomarker in chronic kidney disease. *Clin. Kidney J.* **11** (5), 694–703 (2018). 30288265 10.1093/ckj/sfy037 PMC6165760

[14] Mathur, R., Dreyer, G., Yaqoob, M. M. & Hull, S. A. Ethnic differences in the progression of chronic kidney disease and risk of death in a UK diabetic population: an observational cohort study. *BMJ Open.* **8** (3), e020145. 10.1136/bmjopen-2017-020145 (2018). 29593020 10.1136/bmjopen-2017-020145 PMC5875688

[15] Schultheiss, U. T. & Sekula, P. The promise of metabolomics in decelerating CKD progression in children. *Clin. J. Am. Soc. Nephrol.* **16** (8), 1152–1154 (2021). 34362783 10.2215/CJN.07400521 PMC8455046

[16] Levey, A. S. et al. A new equation to estimate glomerular filtration rate. *Ann. Intern. Med.* **150** (9), 604–612 (2009). 19414839 10.7326/0003-4819-150-9-200905050-00006 PMC2763564

[17] Zhang, H. et al. Identification of Potential Serum Metabolic Biomarkers of Diabetic Kidney Disease: A Widely Targeted Metabolomics Study. *J. Diabetes Res.* **2020** (3049098), 11pages. 10.1155/2020/3049098 (2020). 10.1155/2020/3049098 PMC7072115 32190695

[18] Delporte, C. et al. Workflow4Metabolomics (W4M): A User-Friendly Metabolomics Platform for Analysis of Mass Spectrometry and Nuclear Magnetic Resonance Data. *Curr. Protocols*. **5** (2), e70095. 10.1002/cpz1.70095 (2025). 10.1002/cpz1.70095 39951023

[19] van Iterson, M. et al. Relative power and sample size analysis on gene expression profiling data. *BMC Genom.* **10** (439). 10.1186/1471-2164-10-439 (2009). 10.1186/1471-2164-10-439 PMC2759969 19758461

[20] Kanehisa, M., Furumichi, M., Sato, Y., Matsuura, Y. & Ishiguro-Watanabe, M. KEGG: biological systems database as a model of the real world. *Nucleic Acids Res.* **53** (D1), D672–d677. 10.1093/nar/gkae909 (2025). 39417505 10.1093/nar/gkae909 PMC11701520

[21] Farag, Y. M. K. & El-Sayed, E. *Global Dialysis Perspective: Egypt. Kidney360*, **3**(7),1263–1268. 10.34067/kid.0007482021. (2022). 35919518 10.34067/KID.0007482021 PMC9337895

[22] Shi, C. et al. Multifactorial Diseases of the Heart, Kidneys, Lungs, and Liver and Incident Cancer: Epidemiology and Shared Mechanisms. *Cancers (Basel)*. **15** (3), 729. 10.3390/cancers15030729 (2023). 36765688 10.3390/cancers15030729 PMC9913123

[23] Bello, A. K. et al. Status of care for end stage kidney disease in countries and regions worldwide: international cross sectional survey. *bmj* 367. 10.1136/bmj.l5873 (2019). 10.1136/bmj.l5873 31672760

[24] Hassaballa, M. et al. Egyptian renal data system (ERDS) 2020: an annual report of end-stage kidney disease patients on regular hemodialysis. *J. Egypt. Soc. Nephrol. Transplantation*. **22** (1), 1–28. 10.4103/jesnt.jesnt_37_21 (2022).

[25] Posada-Ayala, M. et al. Identification of a urine metabolomic signature in patients with advanced-stage chronic kidney disease. *Kidney Int.* **85** (1), 103–111. 10.1038/ki.2013.328 (2014). 24048377 10.1038/ki.2013.328

[26] Kobayashi, T. et al. A metabolomics-based approach for predicting stages of chronic kidney disease. *Biochem. Biophys. Res. Commun.* **445** (2), 412–416. 10.1016/j.bbrc.2014.02.021 (2014). 24530913 10.1016/j.bbrc.2014.02.021

[27] Baharum, S. N. & Azizan, K. A. Metabolomics in systems biology. Omics Appl. Syst. Biol. 51–68. 10.1007/978-3-319-98758-3_4 (2018). 10.1007/978-3-319-98758-3_4 30382568

[28] Dahabiyeh, L. A. et al. Metabolomics profiling distinctively identified end-stage renal disease patients from chronic kidney disease patients. *Sci. Rep.* **13** (1), 6161. 10.1038/s41598-023-33377-8 (2023). 37061630 10.1038/s41598-023-33377-8 PMC10105740

[29] Su, X. et al. Serum Biomarkers for Chronic Renal Failure Screening and Mechanistic Understanding: A Global LC-MS‐Based Metabolomics Research. *Evid. Based Complement. Altern. Med.* **2022** (1), 7450977. 10.1155/2022/7450977 (2022). 10.1155/2022/7450977 PMC9356786 35942381

[30] Ling, X. C. & Kuo, K. L. Oxidative stress in chronic kidney disease. *Ren. Replace. Therapy*. **4** (1), 53. 10.1186/s41100-018-0195-2 (2018).

[31] Rapa, S. F., Di Iorio, B. R., Campiglia, P., Heidland, A. & Marzocco, S. Inflammation and oxidative stress in chronic kidney disease-potential therapeutic role of minerals, vitamins and plant-derived metabolites. *Int. J. Mol. Sci.* **21** (1). 10.3390/ijms21010263 (2019). 10.3390/ijms21010263 PMC6981831 31906008

[32] Sun, C. Y. et al. Clinical association between the metabolite of healthy gut microbiota, 3-indolepropionic acid and chronic kidney disease. *Clin. Nutr.* **38** (6), 2945–2948. 10.1016/j.clnu.2018.11.029 (2019). 30612852 10.1016/j.clnu.2018.11.029

[33] Yisireyili, M., Takeshita, K., Saito, S., Murohara, T. & Niwa, T. Indole-3-propionic acid suppresses indoxyl sulfate-induced expression of fibrotic and inflammatory genes in proximal tubular cells. *Nagoya J. Med. Sci.* **79** (4), 477. 10.18999/nagjms.79.4.477 (2017). 29238104 10.18999/nagjms.79.4.477 PMC5719207

[34] Ballanti, M. et al. Decreased circulating IPA levels identify subjects with metabolic comorbidities: A multi-omics study. *Pharmacol. Res.* **204** (107207). 10.1016/j.phrs.2024.107207 (2024). 10.1016/j.phrs.2024.107207 38734193

[35] Ekua, N. et al. Roles of homoarginine in the cardiovascular system. *Appl. Ecol. Environ. Res.* **17** (5). 10.15666/aeer/1705_1056510574 (2019).

[36] Drechsler, C. et al. Homoarginine and Progression of Chronic Kidney Disease: Results from the Mild to Moderate Kidney Disease Study. *PLOS ONE*. **8** (5), e63560. 10.1371/journal.pone.0063560 (2013). 23691067 10.1371/journal.pone.0063560 PMC3655120

[37] Johnson, R. J. et al. Metabolic and Kidney Diseases in the Setting of Climate Change, Water Shortage, and Survival Factors. *J. Am. Soc. Nephrol.* **27** (8), 2247–2256. 10.1681/asn.2015121314 (2016). 27283495 10.1681/ASN.2015121314 PMC4978060

[38] Mor, A., Kalaska, B. & Pawlak, D. Kynurenine pathway in chronic kidney disease: what’s old, what’s new, and what’s next? *Int. J. Tryptophan Res.* **13** (1178646920954882). 10.1177/1178646920954882 (2020). 10.1177/1178646920954882 PMC8862190 35210786

[39] Duranton, F. et al. Plasma and urinary amino acid metabolomic profiling in patients with different levels of kidney function. *Clin. J. Am. Soc. Nephrol.* **9** (1), 37–45. 10.2215/cjn.06000613 (2014). 24235289 10.2215/CJN.06000613 PMC3878706

[40] Liu, X., Xu, X., Shang, R. & Chen, Y. Asymmetric dimethylarginine (ADMA) as an important risk factor for the increased cardiovascular diseases and heart failure in chronic kidney disease. *Nitric oxide*. **78**, 113–120. 10.1016/j.niox.2018.06.004 (2018). 29928990 10.1016/j.niox.2018.06.004 PMC6301111

[41] Wilcox, C. S. Asymmetric dimethylarginine and reactive oxygen species: unwelcome twin visitors to the cardiovascular and kidney disease tables. *Hypertension* **59** (2), 375–381. 10.1161/HYPERTENSIONAHA.111.187310 (2012). 22215715 10.1161/HYPERTENSIONAHA.111.187310 PMC3266466

[42] Rawal, N. et al. Structural specificity of substrate for S-adenosylmethionine protein arginine N-methyltransferases. *Biochim. et Biophys. Acta (BBA)-Protein Struct. Mol. Enzymol.* **1248** (1), 11–18. 10.1016/0167-4838(94)00213-z (1995). 10.1016/0167-4838(94)00213-z 7536038

[43] LEIPER, J. M. et al. Identification of two human dimethylarginine dimethylaminohydrolases with distinct tissue distributions and homology with microbial arginine deiminases. *Biochem. J.* **343** (1), 209–214 (1999). 10493931 PMC1220543

[44] Akyurek, F., Celik, G. & Ozturk, B. Predictive role of methylarginines in renal failure. *Annals Med. Res.* **27** (8), 2129–2133 (2020).

[45] Esquivel, D. G. et al. Kynurenine pathway metabolites and enzymes involved in redox reactions. *Neuropharmacology* **112**, 331–345. 10.1016/j.neuropharm.2016.03.013 (2017). 26970015 10.1016/j.neuropharm.2016.03.013

[46] Pawlak, D., Tankiewicz, A., Matys, T. & Buczko, W. Peripheral distribution of kynurenine metabolites. *J. Physiol. Pharmacol.* **54** (2), 175–189 (2003). 12832720

[47] Silva, R. E. et al. Predictive metabolomic signatures of end-stage renal disease: A multivariate analysis of population-based data. *Biochimie* **152**, 14–30. 10.1016/j.biochi.2018.06.009 (2018). 29913183 10.1016/j.biochi.2018.06.009

[48] Zakrocka, I. & Załuska, W. Kynurenine pathway in kidney diseases. *Pharmacol. Rep.* **74** (1), 27–39. 10.1007/s43440-021-00329-w (2022). 34617264 10.1007/s43440-021-00329-w PMC8786771

[49] Wu, Y., Wang, M. & Yu, B. *Mechanisms of indoleamine 2,3-dioxygenase (IDO)-mediated immunosuppression, in Reference Module in Biomedical Sciences* (Elsevier, 2025).

[50] Hung, S. C. et al. Indoxyl sulfate suppresses endothelial progenitor cell–mediated neovascularization. *Kidney Int.* **89** (3), 574–585. 10.1016/j.kint.2015.11.020 (2016). 26880454 10.1016/j.kint.2015.11.020

[51] Addi, T., Dou, L. & Burtey, S. Tryptophan-derived uremic toxins and thrombosis in chronic kidney disease. *Toxins* **10** (10), 412. 10.3390/toxins10100412 (2018). 30322010 10.3390/toxins10100412 PMC6215213

[52] Hung, S. C., Kuo, K. L., Wu, C. C. & Tarng, D. C. Indoxyl sulfate: a novel cardiovascular risk factor in chronic kidney disease. *J. Am. Heart Association*. **6** (2), e005022. 10.1161/JAHA.116.005022 (2017). 10.1161/JAHA.116.005022 PMC5523780 28174171

[53] Pieniazek, A., Bernasinska-Slomczewska, J. & Hikisz, P. Indoxyl sulfate induces apoptosis in mononuclear blood cells via mitochondrial pathway. *Sci. Rep.* **13** (1), 14044. 10.1038/s41598-023-40824-z (2023). 37640757 10.1038/s41598-023-40824-z PMC10462746

[54] Ellis, R. J. et al. Indoxyl sulfate induces apoptosis and hypertrophy in human kidney proximal tubular cells. *Toxicol. Pathol.* **46** (4), 449–459. 10.1177/0192623318768171 (2018). 29683083 10.1177/0192623318768171

[55] Ribeiro, M. et al. Trimethylamine N-Oxide (TMAO) Plasma Levels in Patients with Different Stages of Chronic Kidney Disease. *Toxins* **17** (1), 15. 10.3390/toxins17010015 (2024). 39852968 10.3390/toxins17010015 PMC11769074

[56] Rhee, E. P. et al. A combined epidemiologic and metabolomic approach improves CKD prediction. *J. Am. Soc. Nephrol.* **24** (8), 1330–1338. 10.1681/ASN.2012101006 (2013). 23687356 10.1681/ASN.2012101006 PMC3736702

[57] Kimura, T. et al. Identification of biomarkers for development of end-stage kidney disease in chronic kidney disease by metabolomic profiling. *Sci. Rep.* **6** (1), 26138. 10.1038/srep26138 (2016). 27188985 10.1038/srep26138 PMC4870629

[58] Tang, W. W. et al. Gut microbiota-dependent trimethylamine N-oxide (TMAO) pathway contributes to both development of renal insufficiency and mortality risk in chronic kidney disease. *Circul. Res.* **116** (3), 448–455. 10.1161/CIRCRESAHA.116.305360 (2015). 10.1161/CIRCRESAHA.116.305360 PMC4312512 25599331

[59] Sun, C. Y. et al. Indoxyl sulfate caused behavioral abnormality and neurodegeneration in mice with unilateral nephrectomy. *Aging (Albany NY)*. **13** (5), 6681. 10.18632/aging.202523 (2021). 33621199 10.18632/aging.202523 PMC7993681

[60] Gryp, T., Vanholder, R., Vaneechoutte, M. & Glorieux, G. p-Cresyl sulfate. *Toxins* **9** (2), 52. 10.3390/toxins9020052 (2017). 28146081 10.3390/toxins9020052 PMC5331431

[61] Vanholder, R., Schepers, E., Pletinck, A., Nagler, E. V. & Glorieux, G. The uremic toxicity of indoxyl sulfate and p-cresyl sulfate: a systematic review. *J. Am. Soc. Nephrol.* **25** (9), 1897–1907. 10.1681/ASN.2013101062 (2014). 24812165 10.1681/ASN.2013101062 PMC4147984

[62] Lin, C. J., Wu, V., Wu, P. C. & Wu, C. J. Meta-analysis of the associations of p-cresyl sulfate (PCS) and indoxyl sulfate (IS) with cardiovascular events and all-cause mortality in patients with chronic renal failure. *PloS one*. **10** (7), e0132589. 10.1371/journal.pone.0132589 (2015). 26173073 10.1371/journal.pone.0132589 PMC4501756

[63] Wang, X. et al. Aberrant gut microbiota alters host metabolome and impacts renal failure in humans and rodents. *Gut* **69** (12), 2131–2142. 10.1136/gutjnl-2019-319766 (2020). 32241904 10.1136/gutjnl-2019-319766 PMC7677483

[64] Moco, S., Martin, F. P. J. & Rezzi, S. Metabolomics view on gut microbiome modulation by polyphenol-rich foods. *J. Proteome Res.* **11** (10), 4781–4790. 10.1021/pr300581s (2012). 22905879 10.1021/pr300581s

[65] Sumida, K. et al. Constipation and incident CKD. *J. Am. Soc. Nephrol.* **28** (4), 1248–1258. 10.1681/ASN.2016060656 (2017). 28122944 10.1681/ASN.2016060656 PMC5373459

[66] Koppe, L., Mafra, D. & Fouque, D. Probiotics and chronic kidney disease. *Kidney Int.* **88** (5), 958–966. 10.1038/ki.2015.255 (2015). 26376131 10.1038/ki.2015.255

[67] Oestvang, J. & Johansen, B. PhospholipaseA2: a key regulator of inflammatory signalling and a connector to fibrosis development in atherosclerosis. *Biochim. Biophys. Acta (BBA) Mol. Cell Biol. Lipids* **1761**(11),1309–1316. 10.1016/j.bbalip.2006.06.003 (2006). 10.1016/j.bbalip.2006.06.003 16904370

[68] Baragetti, A. et al. Low plasma lecithin: cholesterol acyltransferase (LCAT) concentration predicts chronic kidney disease. *J. Clin. Med.* **9** (7), 2289. 10.3390/jcm9072289 (2020). 32708515 10.3390/jcm9072289 PMC7408930

[69] Lee, Y. K. et al. Lysophosphatidylcholine, oxidized low-density lipoprotein and cardiovascular disease in Korean hemodialysis patients: Analysis at 5 years of follow-up. *J. Korean Med. Sci.* **28** (2), 268–273. 10.3346/jkms.2013.28.2.268 (2013). 23400766 10.3346/jkms.2013.28.2.268 PMC3565139

[70] Xu, Z., Yang, S. & Cui, L. Understanding the heterogeneity and dysfunction of HDL in chronic kidney disease: insights from recent reviews. *BMC Nephrol.* **25** (1), 400. 10.1186/s12882-024-03808-3 (2024). 39511510 10.1186/s12882-024-03808-3 PMC11542271

[71] Jones, L. L., McDonald, D. A. & Borum, P. R. Acylcarnitines: role in brain. *Prog. Lipid Res.* **49** (1), 61–75. 10.1016/j.plipres.2009.08.004 (2010). 19720082 10.1016/j.plipres.2009.08.004

[72] Kang, E. et al. Identification of Serum metabolites for predicting chronic kidney disease progression according to chronic kidney disease cause. *Metabolites* **12** (11), 1125 (2022). 36422264 10.3390/metabo12111125 PMC9696352

[73] Yamaguchi, Y. et al. Plasma metabolites associated with chronic kidney disease and renal function in adults from the Baltimore Longitudinal Study of Aging. *Metabolomics* **17** (1), 9. 10.1007/s11306-020-01762-3 (2021). 33428023 10.1007/s11306-020-01762-3 PMC9220986

[74] Bartman, C. R., TeSlaa, T. & Rabinowitz, J. D. Quantitative flux analysis in mammals. *Nat. metabolism*. **3** (7), 896–908. 10.1038/s42255-021-00419-2 (2021). 10.1038/s42255-021-00419-2 PMC9289955 34211182

[75] Gjorgjieva, M., Monteillet, L., Calderaro, J., Mithieux, G. & Rajas, F. Polycystic kidney features of the renal pathology in glycogen storage disease type I: possible evolution to renal neoplasia. *J. Inherit. Metab. Dis.* **41**, 955–963. 10.1007/s10545-018-0207-y (2018). 29869165 10.1007/s10545-018-0207-y

[76] Hourmozdi, J. J. et al. Change in Lactate Levels After Hemodialysis in Patients With End-Stage Renal Disease. *Ann. Emerg. Med.* **71** (6), 737–742. 10.1016/j.annemergmed.2017.09.022 (2018). 29107408 10.1016/j.annemergmed.2017.09.022

[77] Riphagen, I. J. et al. Methylmalonic acid, vitamin B12, renal function, and risk of all-cause mortality in the general population: results from the prospective Lifelines-MINUTHE study. *BMC Med.* **18** (1), 380. 10.1186/s12916-020-01853-x (2020). 33298054 10.1186/s12916-020-01853-x PMC7726887

[78] Tang, Z. et al. Role of purines in regulation of metabolic reprogramming. *Purinergic Signal.* **15** (4), 423–438. 10.1007/s11302-019-09676-z (2019). 31493132 10.1007/s11302-019-09676-z PMC6923298

[79] Del Vecchio, L., Carini, M., Cavalli, A., Locatelli, F. & Laher, I. Oxidative Stress and Chronic Renal Disease – Clinical Aspects. In *Systems Biology of Free Radicals and Antioxidants, Springer Berlin Heidelberg: Berlin, Heidelberg*. 2625–2644. (2014).

[80] Yang, Q., Su, S., Luo, N. & Cao, G. Adenine-induced animal model of chronic kidney disease: current applications and future perspectives. *Ren. Fail.* **46** (1), 2336128. 10.1080/0886022x.2024.2336128 (2024). 38575340 10.1080/0886022X.2024.2336128 PMC10997364

[81] Freed, T. A. et al. Validation of a metabolite panel for a more accurate estimation of glomerular filtration rate using quantitative LC-MS/MS. *Clin. Chem.* **65** (3), 406–418. 10.1373/clinchem.2018.288092 (2019). 30647123 10.1373/clinchem.2018.288092 PMC6646882

[82] Wen, D. et al. Metabolite profiling of CKD progression in the chronic renal insufficiency cohort study. *JCI Insight*. **7** (20). 10.1172/jci.insight.161696 (2022). 10.1172/jci.insight.161696 PMC9714776 36048534

[83] Li, R. et al. Targeted metabolomics study of serum bile acid profile in patients with end-stage renal disease undergoing hemodialysis. *PeerJ* **7**, e7145. 10.7717/peerj.7145 (2019). 31245185 10.7717/peerj.7145 PMC6585905

[84] Zhang, Q. et al. Metabolomic profiling reveals the step-wise alteration of bile acid metabolism in patients with diabetic kidney disease. *Nutr. Diabetes*. **14** (1), 85. 10.1038/s41387-024-00315-0 (2024). 39384774 10.1038/s41387-024-00315-0 PMC11464666

[85] Chu, L., Zhang, K., Zhang, Y., Jin, X. & Jiang, H. Mechanism underlying an elevated serum bile acid level in chronic renal failure patients. *Int. Urol. Nephrol.* **47**, 345–351. 10.1007/s11255-014-0901-0 (2015). 25539619 10.1007/s11255-014-0901-0

[86] Gai, Z. et al. Effect of chronic renal failure on the hepatic, intestinal, and renal expression of bile acid transporters. *Am. J. Physiology-Renal Physiol.* **306** (1), F130–F137. 10.1152/ajprenal.00114.2013 (2014). 10.1152/ajprenal.00114.2013 24197062

[87] Cappuccilli, M. et al. Supplementation and Nutritional Intake of Methyl Donors in Patients with Chronic Kidney Disease: A Critical Review of the Impact on Epigenetic Machinery. *Nutrients* **12** (5). 10.3390/nu12051234 (2020). 10.3390/nu12051234 PMC7281987 32349312

[88] Hughes, A. T., Milan, A. M., Shweihdi, E., Gallagher, J. & Ranganath, L. Method development and validation for analysis of phenylalanine, 4-hydroxyphenyllactic acid and 4-hydroxyphenylpyruvic acid in serum and urine. *JIMD Rep.* **63** (4), 341–350. 10.1002/jmd2.12287 (2022). 35822095 10.1002/jmd2.12287 PMC9259389

[89] Gu, X. et al. Identification of serum biomarkers for chronic kidney disease using serum metabolomics. *Ren. Fail.* **46** (2), 2409346. 10.1080/0886022X.2024.2409346 (2024). 39378112 10.1080/0886022X.2024.2409346 PMC11463012

