# Supplementary material for: Serum metabolomics identifies gut-derived uremic toxins and bile acid dysregulation associated with chronic kidney disease severity
Source: Sci Rep. 2026 Apr 14;16:12375. doi: 10.1038/s41598-026-44271-4 (PMC13083900; doi:10.1038/s41598-026-44271-4)
Supplement: Supplementary file 1 — Supplementary Material 1 [file 41598_2026_44271_MOESM1_ESM.docx]

**Table S1**. Clinical characteristics and data availability in CKD patients

| **Variable** | **eCKD (n =25)** | **ESKD (n=25)** |
| --- | --- | --- |
| Diabetes mellitus | 8/25 (32.00%) | 11/25 (44.00%) |
| Hypertension | 5/25 (20.00%) | 8/25 (32.00%) |
| Dialysis | NA | 13/25 (52.00%) |
| Missing DM data | 3/25 (12.00%) | 4/25 (16.00%) |
| Missing HTN data | 5/25 (20.00%) | 5/25 (20.00%) |
| Missing dialysis data | NA | 4/25 (16.00%) |
