# Supplementary material for: Serum metabolomics identifies gut-derived uremic toxins and bile acid dysregulation associated with chronic kidney disease severity
Source: Sci Rep. 2026 Apr 14;16:12375. doi: 10.1038/s41598-026-44271-4 (PMC13083900; doi:10.1038/s41598-026-44271-4)
Supplement: Supplementary file 2 — Supplementary Material 2 [file 41598_2026_44271_MOESM2_ESM.docx]

**Table S2.** Validation and calibration parameters of targeted metabolites

| **Metabolite** | **Linear range (µM)** | **Equation** | **R^2^** | **LOD (µM)** | **LOQ (µM)** |
| --- | --- | --- | --- | --- | --- |
| IS | 0.5-20 | y=0.97x+0.01 | 0.9995 | 0.13 | 0.40 |
| p-HPhLA | 0.5-20 | y=0.96x+0.01 | 0.9996 | 0.12 | 0.38 |
| TMAO | 0.5-20 | y=1.02x+0.02 | 0.9994 | 0.13 | 0.42 |
| GCDCA | 0.5-20 | y=0.95x+0.03 | 0.9988 | 0.16 | 0.52 |
| XA | 0.5-20 | y=0.97x+0.03 | 0.9992 | 0.14 | 0.45 |

IS, indoxyl sulfate; GCDCA, glycochenodeoxycholic acid; LOD, limits of detection; LOQ, limits of quantification; p-HPhLA, p-hydroxyphenyllactic acid; TMAO, trimethylamine N-oxide; XA, xanthurenic acid.
