# Supplementary material for: Serum metabolomics identifies gut-derived uremic toxins and bile acid dysregulation associated with chronic kidney disease severity
Source: Sci Rep. 2026 Apr 14;16:12375. doi: 10.1038/s41598-026-44271-4 (PMC13083900; doi:10.1038/s41598-026-44271-4)
Supplement: Supplementary file 3 — Supplementary Material 3 [file 41598_2026_44271_MOESM3_ESM.docx]

**Table S3.** XCMS data-processing parameters for untargeted LC–MS metabolomics.

| **Step** | **Parameter** | **Description** | **Setting** |
| --- | --- | --- | --- |
| **Peak detection (centWave)** | Algorithm | Peak detection algorithm | centWave |
|  | ppm | Max tolerated m/z deviation | 3 ppm |
|  | peakwidth | Expected chromatographic peak width (sec) | 5–20 |
|  | snthresh | Signal-to-noise threshold | 10 |
|  | prefilter (k, I) | Minimum scans ≥ intensity threshold | (3, 5000) |
|  | noise | Minimum baseline noise threshold | 1000 |
|  | mzdiff | Minimum m/z difference between peaks | 0 (default) |
|  | mzCenterFun | Method for m/z centering | wMean (default) |
| **Retention-time correction (obiwarp)** | Method | Alignment algorithm | obiwarp |
|  | binSize | m/z binning width | 0.1 |
| **Peak grouping (PeakDensity)** | bw | RT bandwidth (sec) | 5.0 |
|  | mzwid | Width of overlapping m/z slices | 0.01 |
|  | minfrac | Minimum fraction of samples required | 0.80 |
| **Annotation (CAMERA)** | Mode | groupFWHM + findIsotopes | Enabled |
|  | Max charge | Maximum allowed ion charge | 2 |
|  | Adduct/isotope detection | Identification of adducts, isotopes, fragments | Enabled |
|  | RT output | Units and rounding | Minutes, 2 decimals |
