# Supplementary material for: Serum metabolomics identifies gut-derived uremic toxins and bile acid dysregulation associated with chronic kidney disease severity
Source: Sci Rep. 2026 Apr 14;16:12375. doi: 10.1038/s41598-026-44271-4 (PMC13083900; doi:10.1038/s41598-026-44271-4)
Supplement: Supplementary file 4 — Supplementary Material 4 [file 41598_2026_44271_MOESM4_ESM.docx]

**Table S4:** Biochemical characteristics of participants in the validation cohort

| **Parameters** | **Control**  **(n=15)** | **eCKD**  **(n=35)** | **ESKD**  **(n=35)** |
| --- | --- | --- | --- |
| **Age** | 53.33±5.765 | 55.37±7.62 | 59.09±10.351 |
| **Male** | 6 (40.00 %) | 20 (57.14 %) | 18 (51.43 %) |
| **Female** | 9 (60.00 %) | 15 (42.86 %) | 17 (48.57 %) |
| **BMI** | 24.53±5.167 | 26.63±5.088 | 23.89±4.819 |
| **HGB (g/dL)** | 12.39±2.39 | 11.7±2.64 | 10.48±2.45^a^ |
| **Ionized Calcium (mg/dL)** | 4.63±0.98 | 4.26±1.74 | 3.15±1.04^ab^ |
| **Sodium (mEq/L)** | 138.78±10.88 | 129.90±8.84^a^ | 124.64±9.77^a^ |
| **Phosphorous (mg/dL)** | 3.17±1.45 | 3.78±1.47 | 4.67±1.78^a^ |
| **Albumin (g/dL)** | 4.80±1.78 | 3.54±1.13^a^ | 2.99±0.82^a^ |
| **Globulin (g/dL)** | 2.97±0.45 | 3.62±0.78^a^ | 3.18±0.54^b^ |
| **A/G ratio** | 1.64±0.63 | 1.03±0.41^a^ | 0.98±0.35^a^ |
| **Creatinine (mg/dL)** | 0.69±0.15 | 2.78±1.27 | 4.36±2.58 ^ab^ |
| **Serum Urea (mg/dL)** | 30.54±12.65 | 60.54±15.40^a^ | 78.55±10.52^ab^ |
| **eGFR (ml/min/1.73 m^2^)** | 103.00±15.35 | 31.57±20.98^a^ | 21.03±16.71^ab^ |
| **Total Cholesterol (mg/dL)** | 166.78±55.07 | 199.63±55.36 | 200.55± 35.66 |
| **HDL (mg/dL)** | 61.99±11.22 | 55.44±12.55 | 53.55±10.55 |
| **LDL (mg/dL)** | 110.23±29.88 | 114.66±18.99 | 118.64±23.40 |
| **Triglycerides (mg/dL)** | 87.79±20.66 | 93.66±21.66 | 98.54±19.88 |
