# Supplementary material for: Serum metabolomics identifies gut-derived uremic toxins and bile acid dysregulation associated with chronic kidney disease severity
Source: Sci Rep. 2026 Apr 14;16:12375. doi: 10.1038/s41598-026-44271-4 (PMC13083900; doi:10.1038/s41598-026-44271-4)
Supplement: Supplementary file 5 — Supplementary Material 5 [file 41598_2026_44271_MOESM5_ESM.docx]

**Table S5:** Significant metabolite alterations between healthy controls, eCKD and ESKD

| **Metabolite** | **Mode** | **f.value** | ***p*.value** | **FDR** | **Tukey's HSD** |
| --- | --- | --- | --- | --- | --- |
| p-Hydroxyphenyllactic Acid | negative | 793.22 | 2.28E-47 | 1.82E-45 | eCKD-NC; ESKD-NC; ESKD-eCKD |
| Creatinine | negative | 753.23 | 1.20E-46 | 4.80E-45 | eCKD-NC; ESKD-NC; ESKD-eCKD |
| Glycochenodeoxycholate | negative | 548.12 | 2.98E-42 | 7.93E-41 | ESKD-NC; ESKD-eCKD |
| Feruloylputrescine | negative | 461.36 | 6.66E-40 | 1.33E-38 | eCKD-NC; ESKD-NC |
| Taurocholate | negative | 352.77 | 2.68E-36 | 4.29E-35 | ESKD-NC; ESKD-eCKD |
| Inosine | negative | 325 | 3.26E-35 | 4.35E-34 | ESKD-NC; ESKD-eCKD |
| 5-dodecenoate (12:1n7) | negative | 301.55 | 3.15E-34 | 3.60E-33 | eCKD-NC; ESKD-NC; ESKD-eCKD |
| Ornithine | negative | 204.19 | 3.11E-29 | 2.87E-28 | eCKD-NC; ESKD-NC; ESKD-eCKD |
| Oxalic acid | negative | 203.93 | 3.23E-29 | 2.87E-28 | eCKD-NC; ESKD-NC |
| Phosphocholine | negative | 175.06 | 2.48E-27 | 1.99E-26 | eCKD-NC; ESKD-NC |
| Deoxycholic acid | negative | 163.17 | 1.77E-26 | 1.29E-25 | ESKD-NC; ESKD-eCKD |
| Citric acid | negative | 162.31 | 2.05E-26 | 1.33E-25 | eCKD-NC; ESKD-NC |
| Acetyl carnitine | negative | 162.02 | 2.16E-26 | 1.33E-25 | ESKD-NC; ESKD-eCKD |
| Calcitriol | negative | 158.78 | 3.78E-26 | 2.16E-25 | ESKD-NC; ESKD-eCKD |
| Succinic Acid | negative | 151.06 | 1.49E-25 | 7.96E-25 | eCKD-NC; ESKD-NC; ESKD-eCKD |
| Adipic acid | negative | 149.86 | 1.86E-25 | 9.28E-25 | ESKD-NC; ESKD-eCKD |
| Asparagine | negative | 141.72 | 8.49E-25 | 3.99E-24 | eCKD-NC; ESKD-NC; ESKD-eCKD |
| Cinnamic Acid | negative | 138.66 | 1.53E-24 | 6.81E-24 | eCKD-NC; ESKD-NC |
| Arginine | negative | 125.43 | 2.23E-23 | 9.39E-23 | eCKD-NC; ESKD-NC; ESKD-eCKD |
| Hypotaurine | negative | 105.54 | 1.97E-21 | 7.87E-21 | eCKD-NC; ESKD-NC |
| Thymol sulfate | negative | 104.96 | 2.27E-21 | 8.63E-21 | ESKD-NC; ESKD-eCKD |
| 3-hydroxydecanoate | negative | 103.65 | 3.11E-21 | 1.13E-20 | ESKD-NC; ESKD-eCKD |
| Myristoleate | negative | 102.84 | 3.79E-21 | 1.32E-20 | eCKD-NC; ESKD-NC |
| Lactate | negative | 101.26 | 5.61E-21 | 1.87E-20 | ESKD-NC; ESKD-eCKD |
| Indoxyl sulphate | negative | 99.465 | 8.79E-21 | 2.81E-20 | ESKD-NC; ESKD-eCKD |
| N-formyl methionine | negative | 93.741 | 3.84E-20 | 1.18E-19 | ESKD-NC; ESKD-eCKD |
| Phenylalanine | negative | 86.462 | 2.76E-19 | 8.18E-19 | ESKD-NC; ESKD-eCKD |
| Malic Acid | negative | 85.279 | 3.85E-19 | 1.10E-18 | eCKD-NC; ESKD-NC |
| Cholesteryl sulphate | negative | 84.921 | 4.26E-19 | 1.17E-18 | eCKD-NC; ESKD-NC |
| Hippuric acid | negative | 78.537 | 2.73E-18 | 7.27E-18 | eCKD-NC; ESKD-NC |
| Citrulline | negative | 76.247 | 5.44E-18 | 1.40E-17 | eCKD-NC; ESKD-NC |
| Kynurenic acid | negative | 71.449 | 2.43E-17 | 6.08E-17 | ESKD-NC; ESKD-eCKD |
| Phenol sulfate | negative | 68.098 | 7.22E-17 | 1.75E-16 | ESKD-NC; ESKD-eCKD |
| 16-hydroxypalmitate | negative | 65.611 | 1.66E-16 | 3.89E-16 | ESKD-NC; ESKD-eCKD |
| Lithocholic acid | negative | 62.793 | 4.35E-16 | 9.94E-16 | ESKD-NC; ESKD-eCKD |
| Octadecanedioate (C18) | negative | 62.394 | 5.00E-16 | 1.11E-15 | ESKD-NC; ESKD-eCKD |
| Thymidine | negative | 58.813 | 1.79E-15 | 3.87E-15 | ESKD-NC; ESKD-eCKD |
| Oleic acid | negative | 52.561 | 1.88E-14 | 3.95E-14 | ESKD-NC; ESKD-eCKD |
| Stearic acid | negative | 50.976 | 3.49E-14 | 7.17E-14 | ESKD-NC; ESKD-eCKD |
| Arachidonic | negative | 46.624 | 2.06E-13 | 4.11E-13 | eCKD-NC; ESKD-NC; ESKD-eCKD |
| Homocysteine | negative | 39.346 | 4.99E-12 | 9.74E-12 | ESKD-NC; ESKD-eCKD |
| Adrenate | negative | 37.41 | 1.23E-11 | 2.35E-11 | eCKD-NC; ESKD-NC |
| P-cresyl sulphate | negative | 37.03 | 1.47E-11 | 2.74E-11 | eCKD-NC; ESKD-NC |
| Glycolithocholate sulphate | negative | 36.89 | 1.58E-11 | 2.86E-11 | eCKD-NC; ESKD-NC |
| Pyruvate | negative | 35.909 | 2.52E-11 | 4.48E-11 | ESKD-NC; ESKD-eCKD |
| Homovanillate | negative | 35.142 | 3.66E-11 | 6.36E-11 | ESKD-NC; ESKD-eCKD |
| 4-vinylphenol sulfate | negative | 34.469 | 5.09E-11 | 8.66E-11 | eCKD-NC; ESKD-NC; ESKD-eCKD |
| Ursodeoxycholate | negative | 32.897 | 1.11E-10 | 1.86E-10 | eCKD-NC; ESKD-NC |
| Sarcosine | negative | 27.999 | 1.45E-09 | 2.37E-09 | ESKD-NC; ESKD-eCKD |
| Linoleic acid | negative | 26.336 | 3.64E-09 | 5.82E-09 | ESKD-NC; ESKD-eCKD |
| Aspartate | negative | 24.073 | 1.32E-08 | 2.08E-08 | eCKD-NC; ESKD-NC |
| 2-Keto Tridecanoic Acid | negative | 22.387 | 3.58E-08 | 5.51E-08 | eCKD-NC; ESKD-NC |
| Xanthine | negative | 22.327 | 3.71E-08 | 5.60E-08 | eCKD-NC; ESKD-NC |
| heptanoate (7:0) | negative | 22.008 | 4.50E-08 | 6.66E-08 | ESKD-NC; ESKD-eCKD |
| 2-hydoxystearate | negative | 20.443 | 1.17E-07 | 1.71E-07 | eCKD-NC; ESKD-NC; ESKD-eCKD |
| Myo-inositol | negative | 20.326 | 1.26E-07 | 1.80E-07 | ESKD-NC; ESKD-eCKD |
| Hydroxyisocaproate | negative | 19.71 | 1.86E-07 | 2.60E-07 | ESKD-NC; ESKD-eCKD |
| 17-Methyloctadecanoic Acid | negative | 19.184 | 2.59E-07 | 3.57E-07 | eCKD-NC; ESKD-NC |
| Methionine | negative | 17.106 | 9.96E-07 | 1.35E-06 | eCKD-NC; ESKD-NC; ESKD-eCKD |
| Glutamate | negative | 14.634 | 5.33E-06 | 7.11E-06 | eCKD-NC; ESKD-NC |
| Ceramide | negative | 14.095 | 7.77E-06 | 1.02E-05 | eCKD-NC; ESKD-NC |
| N-acetyl-alanine | negative | 13.498 | 1.19E-05 | 1.53E-05 | eCKD-NC; ESKD-NC |
| 10-heptadecenoate (17:1n7) | negative | 13.138 | 1.54E-05 | 1.95E-05 | eCKD-NC; ESKD-NC |
| Nonadecanoic acid | negative | 12.611 | 2.25E-05 | 2.77E-05 | eCKD-NC; ESKD-NC |
| Eicosenoic acid | negative | 11.334 | 5.76E-05 | 6.98E-05 | eCKD-NC; ESKD-NC |
| Oleic Acid-2,6-diisopropylanilide | negative | 10.71 | 9.21E-05 | 0.00010992 | eCKD-NC; ESKD-NC |
| Undecanoic acid | negative | 10.288 | 0.0001269 | 0.0001493 | ESKD-NC |
| Nonadecanal | negative | 9.981 | 0.00016068 | 0.0001863 | eCKD-NC; ESKD-NC |
| phenylacetate | negative | 9.7868 | 0.00018667 | 0.00021334 | eCKD-NC; ESKD-eCKD |
| Decanoic acid | negative | 9.7557 | 0.00019123 | 0.00021547 | eCKD-NC |
| 2-hydroxyisovalerate | negative | 7.8643 | 0.00085512 | 0.00095013 | ESKD-eCKD |
| Catechol sulfate | negative | 7.2213 | 0.0014454 | 0.001584 | eCKD-NC |
| 3-Hydroxyisovalerate | negative | 5.733 | 0.0050313 | 0.0054393 | ESKD-NC |
| 4-Methyl-2-oxopentanoate | negative | 4.9309 | 0.010051 | 0.010721 | ESKD-eCKD |
| hydrochlorothiazide | negative | 4.6039 | 0.013383 | 0.014087 | ESKD-NC |
| Laurate (12:0) | negative | 4.5205 | 0.014402 | 0.014963 | ESKD-NC; ESKD-eCKD |
| Palmitoleate (16:1n17) | negative | 4.2185 | 0.018811 | 0.019294 | eCKD-NC |
| Myristate | negative | 3.7863 | 0.027676 | 0.028026 | ESKD-NC |
| 3-methyl-2-oxovalerate | negative | 3.66 | 0.031007 | 0.031007 | ESKD-eCKD |
| Phenylacetylglutamine | positive | 2168.2 | 1.28E-61 | 1.44E-59 | eCKD-NC; ESKD-NC; ESKD-eCKD |
| Trimethylamine N-Oxide | positive | 1587.1 | 3.67E-57 | 2.08E-55 | eCKD-NC; ESKD-NC; ESKD-eCKD |
| Ascorbate | positive | 1406.9 | 1.91E-55 | 7.18E-54 | eCKD-NC; ESKD-NC |
| Methylguanidine | positive | 1372 | 4.33E-55 | 1.22E-53 | eCKD-NC; ESKD-NC; ESKD-eCKD |
| 3-hydroxyanthranilate | positive | 1357.3 | 6.17E-55 | 1.39E-53 | ESKD-NC; ESKD-eCKD |
| Glycyl-proline | positive | 1201 | 3.34E-53 | 6.30E-52 | ESKD-NC; ESKD-eCKD |
| Lysyl-proline | positive | 1120.7 | 3.18E-52 | 5.13E-51 | ESKD-NC; ESKD-eCKD |
| Threonine | positive | 864.22 | 1.44E-48 | 2.04E-47 | eCKD-NC; ESKD-NC; ESKD-eCKD |
| Lysophosphatidylcholine (18:1) | positive | 769.75 | 5.98E-47 | 7.51E-46 | eCKD-NC; ESKD-NC; ESKD-eCKD |
| Creatinine | positive | 753.23 | 1.20E-46 | 1.35E-45 | eCKD-NC; ESKD-NC; ESKD-eCKD |
| Anthranilate | positive | 653.74 | 1.11E-44 | 1.14E-43 | eCKD-NC; ESKD-NC; ESKD-eCKD |
| Urea | positive | 600.74 | 1.63E-43 | 1.54E-42 | eCKD-NC; ESKD-NC; ESKD-eCKD |
| Homogentisate | positive | 516.07 | 1.99E-41 | 1.62E-40 | ESKD-NC; ESKD-eCKD |
| Docosahexaenoic acid | positive | 515.95 | 2.00E-41 | 1.62E-40 | eCKD-NC; ESKD-NC |
| Pseudouridine | positive | 504.48 | 4.06E-41 | 3.06E-40 | eCKD-NC; ESKD-NC; ESKD-eCKD |
| Methylmalonate | positive | 501.6 | 4.86E-41 | 3.43E-40 | eCKD-NC; ESKD-NC; ESKD-eCKD |
| Taurine | positive | 500.41 | 5.23E-41 | 3.48E-40 | eCKD-NC; ESKD-NC; ESKD-eCKD |
| Glycyl-valine | positive | 483.52 | 1.54E-40 | 9.65E-40 | ESKD-NC; ESKD-eCKD |
| Lactose | positive | 481.62 | 1.74E-40 | 1.03E-39 | eCKD-NC; ESKD-NC |
| Xanthurenic acid | positive | 462.24 | 6.28E-40 | 3.55E-39 | ESKD-NC; ESKD-eCKD |
| Pipecolate | positive | 457.85 | 8.46E-40 | 4.55E-39 | ESKD-NC; ESKD-eCKD |
| N-Acetylneuraminic Acid | positive | 427.7 | 7.06E-39 | 3.63E-38 | eCKD-NC; ESKD-NC; ESKD-eCKD |
| 5-hydroxyindoleacetic acid | positive | 401.7 | 4.93E-38 | 2.42E-37 | ESKD-NC; ESKD-eCKD |
| N-hydroxy-valine | positive | 395.44 | 8.01E-38 | 3.77E-37 | ESKD-NC; ESKD-eCKD |
| Biliverdin | positive | 394.47 | 8.64E-38 | 3.91E-37 | ESKD-NC; ESKD-eCKD |
| Aspartyl-phenylalanine | positive | 380.22 | 2.69E-37 | 1.17E-36 | ESKD-NC; ESKD-eCKD |
| 4-pyridoxate | positive | 376.65 | 3.59E-37 | 1.50E-36 | eCKD-NC; ESKD-NC |
| 3-Phenylpropionate | positive | 374.03 | 4.45E-37 | 1.80E-36 | eCKD-NC; ESKD-NC; ESKD-eCKD |
| 2-phenylglycine | positive | 368.99 | 6.76E-37 | 2.63E-36 | ESKD-NC; ESKD-eCKD |
| Palmitic acid | positive | 359.84 | 1.46E-36 | 5.50E-36 | eCKD-NC; ESKD-NC |
| Adenine | positive | 356.74 | 1.90E-36 | 6.93E-36 | eCKD-NC; ESKD-NC; ESKD-eCKD |
| L-Glutamine | positive | 340.73 | 7.74E-36 | 2.73E-35 | ESKD-NC; ESKD-eCKD |
| Dimethylarginine | positive | 340.04 | 8.24E-36 | 2.82E-35 | eCKD-NC; ESKD-NC; ESKD-eCKD |
| Betaine | positive | 327.1 | 2.68E-35 | 8.91E-35 | eCKD-NC; ESKD-NC; ESKD-eCKD |
| Methioninesulfoxide | positive | 319.96 | 5.24E-35 | 1.69E-34 | eCKD-NC; ESKD-NC; ESKD-eCKD |
| Palmitoylcarnitine | positive | 310.91 | 1.25E-34 | 3.92E-34 | eCKD-NC; ESKD-NC |
| Allantoin | positive | 306.69 | 1.89E-34 | 5.77E-34 | eCKD-NC; ESKD-NC; ESKD-eCKD |
| Theobromine | positive | 301.04 | 3.31E-34 | 9.85E-34 | eCKD-NC; ESKD-NC; ESKD-eCKD |
| Glycerophosphoryl choline | positive | 296.76 | 5.10E-34 | 1.48E-33 | eCKD-NC; ESKD-NC; ESKD-eCKD |
| Glycocholate | positive | 295.55 | 5.77E-34 | 1.63E-33 | ESKD-NC; ESKD-eCKD |
| Dimethylglycine | positive | 293.64 | 7.01E-34 | 1.93E-33 | eCKD-NC; ESKD-NC; ESKD-eCKD |
| Valine | positive | 279.54 | 3.07E-33 | 8.25E-33 | eCKD-NC; ESKD-NC; ESKD-eCKD |
| paraxanthine | positive | 273.68 | 5.77E-33 | 1.52E-32 | eCKD-NC; ESKD-NC; ESKD-eCKD |
| Glutamyl-valine | positive | 264.53 | 1.59E-32 | 4.08E-32 | ESKD-NC; ESKD-eCKD |
| Porphobilinogen | positive | 252.59 | 6.25E-32 | 1.57E-31 | eCKD-NC; ESKD-NC; ESKD-eCKD |
| Suberate | positive | 241.65 | 2.31E-31 | 5.68E-31 | eCKD-NC; ESKD-NC; ESKD-eCKD |
| Prolyl-leucine | positive | 234.06 | 5.90E-31 | 1.42E-30 | ESKD-NC; ESKD-eCKD |
| Cystine | positive | 201.52 | 4.54E-29 | 1.07E-28 | eCKD-NC; ESKD-NC; ESKD-eCKD |
| Homoarginine | positive | 197.4 | 8.20E-29 | 1.89E-28 | eCKD-NC; ESKD-eCKD |
| Xanthosine | positive | 186.19 | 4.35E-28 | 9.83E-28 | eCKD-NC; ESKD-NC |
| Cortisol | positive | 185.83 | 4.60E-28 | 1.02E-27 | eCKD-NC; ESKD-NC; ESKD-eCKD |
| Butyrylcarnitine | positive | 171.82 | 4.20E-27 | 9.12E-27 | eCKD-NC; ESKD-NC; ESKD-eCKD |
| Cortisone | positive | 164.02 | 1.53E-26 | 3.27E-26 | eCKD-NC; ESKD-NC; ESKD-eCKD |
| Norleucine | positive | 150.21 | 1.74E-25 | 3.64E-25 | ESKD-NC; ESKD-eCKD |
| Argininosuccinic Acid | positive | 150.09 | 1.78E-25 | 3.66E-25 | eCKD-NC; ESKD-NC |
| Glycyl-tyrosine | positive | 135.74 | 2.72E-24 | 5.48E-24 | eCKD-NC; ESKD-NC |
| Hydroquinone | positive | 134.71 | 3.33E-24 | 6.61E-24 | ESKD-NC; ESKD-eCKD |
| Theophylline | positive | 133.1 | 4.60E-24 | 8.96E-24 | eCKD-NC; ESKD-NC; ESKD-eCKD |
| N6-Methyllysine | positive | 121.09 | 5.65E-23 | 1.07E-22 | eCKD-NC; ESKD-NC; ESKD-eCKD |
| Indolepropionate | positive | 121.06 | 5.67E-23 | 1.07E-22 | eCKD-NC; ESKD-eCKD |
| Lysyl-glutamate | positive | 119.87 | 7.35E-23 | 1.36E-22 | eCKD-NC; ESKD-NC |
| Sphingomyelin | positive | 110.63 | 5.90E-22 | 1.08E-21 | eCKD-NC; ESKD-NC |
| Tyrosine | positive | 99.078 | 9.69E-21 | 1.74E-20 | eCKD-NC; ESKD-NC; ESKD-eCKD |
| Prolyl-lysine | positive | 98.263 | 1.19E-20 | 2.08E-20 | ESKD-NC; ESKD-eCKD |
| N-acetylornithine | positive | 98.243 | 1.20E-20 | 2.08E-20 | eCKD-NC; ESKD-NC; ESKD-eCKD |
| Serine | positive | 95.802 | 2.24E-20 | 3.84E-20 | ESKD-NC; ESKD-eCKD |
| Tryptophan | positive | 94.502 | 3.14E-20 | 5.30E-20 | eCKD-NC; ESKD-NC; ESKD-eCKD |
| Dihydrocapsaicin | positive | 89.788 | 1.10E-19 | 1.84E-19 | eCKD-NC; ESKD-NC; ESKD-eCKD |
| 1-aminobutyrate | positive | 76.792 | 4.61E-18 | 7.55E-18 | ESKD-NC; ESKD-eCKD |
| Prolyl-tyrosine | positive | 74.521 | 9.26E-18 | 1.49E-17 | ESKD-NC; ESKD-eCKD |
| 3-methylhistidine | positive | 74.473 | 9.40E-18 | 1.50E-17 | eCKD-NC; ESKD-NC |
| Dihydrobiopterin | positive | 73.805 | 1.16E-17 | 1.82E-17 | eCKD-NC; ESKD-NC |
| Kynurenic acid | positive | 71.449 | 2.43E-17 | 3.77E-17 | ESKD-NC; ESKD-eCKD |
| Diethanolamine | positive | 65.471 | 1.74E-16 | 2.65E-16 | eCKD-NC; ESKD-NC; ESKD-eCKD |
| Cytosine | positive | 63.857 | 3.01E-16 | 4.54E-16 | ESKD-NC; ESKD-eCKD |
| Uric acid | positive | 55.625 | 5.81E-15 | 8.64E-15 | eCKD-NC; ESKD-NC; ESKD-eCKD |
| Choline | positive | 36.604 | 1.81E-11 | 2.65E-11 | eCKD-NC; ESKD-NC |
| 5-oxoproline | positive | 35.85 | 2.59E-11 | 3.76E-11 | eCKD-NC; ESKD-NC |
| Histidine | positive | 34.62 | 4.72E-11 | 6.76E-11 | eCKD-NC; ESKD-NC; ESKD-eCKD |
| N-acetylglutamine | positive | 33.909 | 6.71E-11 | 9.48E-11 | eCKD-NC; ESKD-NC; ESKD-eCKD |
| Beta-Hydroxybutyrate | positive | 32.554 | 1.33E-10 | 1.85E-10 | eCKD-NC; ESKD-NC; ESKD-eCKD |
| Proline | positive | 30.56 | 3.70E-10 | 5.10E-10 | eCKD-NC; ESKD-eCKD |
| γ -Glutamyl-phenylalanine | positive | 30.367 | 4.10E-10 | 5.58E-10 | eCKD-NC; ESKD-NC; ESKD-eCKD |
| 3-methoxytyrosine | positive | 25.667 | 5.30E-09 | 7.13E-09 | eCKD-NC; ESKD-NC |
| Acetone | positive | 24.128 | 1.28E-08 | 1.70E-08 | eCKD-NC; ESKD-NC; ESKD-eCKD |
| Hydroxyproline | positive | 20.174 | 1.39E-07 | 1.80E-07 | eCKD-NC; ESKD-NC |
| γ -Glutamyl-methionine | positive | 19.604 | 1.98E-07 | 2.55E-07 | ESKD-NC; ESKD-eCKD |
| 4-hydroxyphenyl acetic acid | positive | 15.891 | 2.25E-06 | 2.82E-06 | eCKD-NC; ESKD-NC; ESKD-eCKD |
| Cysteinyl-glycine | positive | 11.942 | 3.66E-05 | 4.55E-05 | eCKD-NC; ESKD-NC; ESKD-eCKD |
| Glucose | positive | 11.392 | 5.51E-05 | 6.77E-05 | eCKD-NC; ESKD-NC |
| Orotate | positive | 10.839 | 8.35E-05 | 0.00010149 | eCKD-NC; ESKD-NC |
| Niacinamide | positive | 10.596 | 0.00010034 | 0.00012062 | eCKD-NC; ESKD-NC |
| γ -Glutamyl-tyrosine | positive | 9.4964 | 0.00023391 | 0.00027823 | ESKD-NC |
| Spermine | positive | 9.4678 | 0.00023918 | 0.00028153 | eCKD-NC; ESKD-NC |
| Gluconic acid | positive | 9.0405 | 0.00033427 | 0.00038941 | eCKD-NC; ESKD-NC |
| Serotonin | positive | 8.8057 | 0.00040239 | 0.00046398 | ESKD-NC; ESKD-eCKD |
| Glucosamine | positive | 8.6824 | 0.0004437 | 0.00050645 | eCKD-NC; ESKD-NC |
| Pantothenic Acid | positive | 8.5795 | 0.00048155 | 0.00054415 | ESKD-NC |
| Glutathione | positive | 8.4627 | 0.00052852 | 0.00059132 | ESKD-NC |
| Hexanoylcarnitine | positive | 8.3011 | 0.00060145 | 0.00066632 | ESKD-NC |
| Alpha-Ketoglutarate | positive | 8.0209 | 0.00075347 | 0.00082662 | eCKD-NC; ESKD-NC |
| γ-glutamyl-leucine | positive | 7.973 | 0.00078316 | 0.00085093 | ESKD-NC |
| Bilirubin | positive | 7.802 | 0.00089944 | 0.00096797 | ESKD-NC |
| Glycerol 3-Phosphate | positive | 7.7358 | 0.00094908 | 0.0010118 | eCKD-NC; ESKD-NC |
| Indoleacetate | positive | 7.4566 | 0.0011917 | 0.0012585 | ESKD-NC |
| 1-methylxanthine | positive | 7.1158 | 0.0015766 | 0.0016496 | eCKD-NC; ESKD-NC |
| Leucine | positive | 6.7476 | 0.002139 | 0.0022175 | eCKD-NC; ESKD-NC |
| Isoleucine | positive | 6.1894 | 0.0034151 | 0.0035083 | eCKD-NC; ESKD-NC |
| Malonate | positive | 5.8314 | 0.0046263 | 0.0047097 | ESKD-NC |
| Carnosine | positive | 4.9925 | 0.0095261 | 0.0096111 | ESKD-NC |
| Vitamin B2 | positive | 4.8068 | 0.011201 | 0.011201 | ESKD-NC |

Group differences were assessed using one-way ANOVA (F-values, *p*-values) with Tukey’s HSD post-hoc testing. FDR correction was applied using the Benjamini–Hochberg method. eCKD: early-stage chronic kidney disease, ESKD: end-stage kidney disease, FDR: false discovery rate, NC: healthy controls.
