# Supplementary material for: Serum metabolomics identifies gut-derived uremic toxins and bile acid dysregulation associated with chronic kidney disease severity
Source: Sci Rep. 2026 Apr 14;16:12375. doi: 10.1038/s41598-026-44271-4 (PMC13083900; doi:10.1038/s41598-026-44271-4)
Supplement: Supplementary file 6 — Supplementary Material 6 [file 41598_2026_44271_MOESM6_ESM.docx]

**Table S6:** Summary of significant differential metabolite profiling with VIP scores, fold changes, and pathway associations

| **Metabolite** | **m/z** | **RT** | **Mode** | **Groups** | **VIP** | **FC** | **log2(FC)** | **FDR** | **LOG10(p)** | **Up/ Down** | **Pathway** |
| --- | --- | --- | --- | --- | --- | --- | --- | --- | --- | --- | --- |
| Arginine | 175.10 | 2.60 | negative | eCKD/NC | 1.7131328 | 2 | 1 | 7.03E-24 | 23.153 | Up | Amino acid metabolism |
| Feruloylputrescine | 317.20 | 7.40 | negative | eCKD/NC | 1.7124022 | 0.31935 | -1.64679 | 1.21E-24 | 23.916 | Down | Polyamine metabolism, Ferulate metabolism |
| Oxalate | 89.00 | 1.20 | negative | eCKD/NC | 1.6937447 | 4.3906 | 2.134418 | 3.96E-23 | 22.403 | Up | Amino acid metabolism |
| Phosphocholine | 184.10 | 4.40 | negative | eCKD/NC | 1.6759053 | 18.984 | 4.246712 | 1.28E-20 | 19.894 | Up | Phospholipid metabolism |
| Citric acid | 191.00 | 2.70 | negative | eCKD/NC | 1.6739528 | 5.7601 | 2.526094 | 1.31E-20 | 19.883 | Up | TCA cycle |
| Cinnamic acid | 147.00 | 3.30 | negative | eCKD/NC | 1.6697401 | 5.173 | 2.371001 | 1.78E-20 | 19.749 | Up | Phenylpropanoid metabolism |
| Succinate | 117.00 | 2.00 | negative | eCKD/NC | 1.6580881 | 5.8318 | 2.543941 | 2.67E-19 | 18.573 | Up | TCA cycle |
| Hypotaurine | 108.01 | 1.80 | negative | eCKD/NC | 1.6479906 | 2.4581 | 1.297544 | 1.03E-18 | 17.989 | Up | Taurine and hypotaurine metabolism |
| Creatinine | 112.10 | 6.90 | negative | eCKD/NC | 1.6427102 | 8.3148 | 3.055682 | 4.66E-18 | 17.332 | Up | Amino acid metabolism |
| Malic Acid | 133.01 | 2.30 | negative | eCKD/NC | 1.635291 | 3.5626 | 1.832931 | 2.18E-18 | 17.661 | Up | TCA cycle |
| Cholesteryl sulphate | 465.35 | 7.10 | negative | eCKD/NC | 1.5888984 | 3.1891 | 1.673149 | 1.10E-15 | 14.957 | Up | Steroid biosynthesis |
| Myristoleate | 225.18 | 5.70 | negative | eCKD/NC | 1.5730771 | 2.6629 | 1.412998 | 1.66E-14 | 13.78 | Up | Fatty acid metabolism |
| Citrulline | 174.09 | 2.00 | negative | eCKD/NC | 1.4930032 | 2.1097 | 1.077038 | 3.61E-12 | 11.442 | Up | Urea cycle |
| Hippurate | 178.05 | 3.70 | negative | eCKD/NC | 1.483459 | 2.7589 | 1.464093 | 7.09E-12 | 11.149 | Up | Amino acid metabolism |
| p-cresyl Sulfate | 187.01 | 8.74 | negative | eCKD/NC | 1.4543118 | 1.51 | 0.594549 | 5.47E-11 | 10.262 | Up | Amino acid metabolism (Gut-microbiota) |
| p-Hydroxyphenyllactic Acid | 181.10 | 3.50 | negative | eCKD/NC | 1.424605 | 4.9901 | 2.319069 | 1.58E-10 | 9.8025 | Up | Amino acid metabolism |
| Asparagine | 131.05 | 3.90 | negative | eCKD/NC | 1.4176924 | 0.665 | -0.58857 | 3.27E-10 | 9.4861 | Down | Amino acid metabolism |
| Aspartate | 132.00 | 2.10 | negative | eCKD/NC | 1.4117466 | 1.8088 | 0.855033 | 3.53E-10 | 9.4524 | Up | Urea cycle, Amino acid metabolism, TCA Cycle |
| glycolithocholate sulphate | 255.63 | 6.03 | negative | eCKD/NC | 1.4046167 | 0.667 | -0.58424 | 3.53E-10 | 9.4524 | Down | Bile acid metabolism |
| Xanthine | 151.03 | 1.70 | negative | eCKD/NC | 1.3268407 | 1.6994 | 0.765025 | 1.28E-08 | 7.8925 | Up | Purine metabolism |
| 2-hydoxystearate | 299.26 | 14.55 | negative | eCKD/NC | 1.2427836 | 1.5008 | 0.585732 | 2.82E-07 | 6.5491 | Up | Fatty acid metabolism |
| 17-methyloctadecanoic acid | 285.30 | 9.20 | negative | eCKD/NC | 1.1276521 | 1.6218 | 0.697596 | 2.25E-05 | 4.6481 | Up | Fatty acid metabolism |
| 2-Keto Tridecanoic Acid | 213.00 | 5.60 | negative | eCKD/NC | 1.0467089 | 0.34841 | -1.52114 | 0.0001093 | 3.9613 | Down | Fatty acid metabolism |
| Nonadecanoate | 297.28 | 3.40 | negative | eCKD/NC | 1.0032665 | 1.8667 | 0.90049 | 0.000174 | 3.7595 | Up | Fatty acid metabolism |
| Glutamate | 146.00 | 4.30 | negative | eCKD/NC | 0.9363383 | 1.7567 | 0.812868 | 0.0004888 | 3.3109 | Up | Amino acid metabolism |
| Eicosenoate | 309.30 | 10.10 | negative | eCKD/NC | 0.9142887 | 1.5 | 0.584963 | 0.0005811 | 3.2357 | Up | Fatty acid metabolism |
| Oleic acid-2,6-diisopropylanilide | 443.30 | 10.50 | negative | eCKD/NC | 0.8989977 | 1.8333 | 0.874443 | 0.0010662 | 2.9722 | Up | Fatty acid metabolism |
| Ceramide | 580.49 | 19.46 | negative | eCKD/NC | 0.894598 | 1.5 | 0.584963 | 0.001191 | 2.9241 | Up | Phospholipid metabolism |
| Arachidonate | 303.20 | 16.26 | negative | eCKD/NC | 0.8843654 | 1.6286 | 0.703632 | 0.0009203 | 3.0361 | Up | Fatty acid metabolism |
| Nonadecanal | 283.30 | 9.10 | negative | eCKD/NC | 0.8076864 | 1.9104 | 0.933875 | 0.0026203 | 2.5816 | Up | Fatty acid metabolism |
| Kynurenic acid | 188.04 | 5.90 | negative | eCKD/NC | 0.6521907 | 0.668 | -0.58208 | 0.022572 | 1.6464 | Down | Tryptophan metabolism |
| Creatinine | 112.10 | 6.90 | negative | ESKD/NC | 1.4159265 | 24.113 | 4.591739 | 8.49E-33 | 32.071 | Up | Amino acid metabolism |
| p-Hydroxyphenyllactic Acid | 181.10 | 3.50 | negative | ESKD/NC | 1.4128805 | 24.512 | 4.615416 | 1.86E-31 | 30.73 | Up | Amino acid metabolism |
| Glycochenodeoxycholate | 448.31 | 9.95 | negative | ESKD/NC | 1.3968465 | 3.7755 | 1.916668 | 1.76E-27 | 26.753 | Up | Bile acid metabolism |
| Feruloylputrescine | 317.20 | 7.40 | negative | ESKD/NC | 1.3801563 | 0.30065 | -1.73384 | 2.15E-24 | 23.667 | Down | Polyamine metabolism, Ferulate metabolism |
| Inosine | 267.07 | 1.23 | negative | ESKD/NC | 1.3674953 | 2.6804 | 1.422448 | 4.10E-22 | 21.388 | Up | Purine metabolism |
| Phosphocholine | 184.10 | 4.40 | negative | ESKD/NC | 1.3637885 | 20.016 | 4.323082 | 1.75E-21 | 20.758 | Up | Phospholipid metabolism |
| Taurocholate | 514.28 | 6.67 | negative | ESKD/NC | 1.3633915 | 2.5806 | 1.367707 | 1.46E-21 | 20.835 | Up | Bile acid metabolism |
| Oxalate | 89.00 | 1.20 | negative | ESKD/NC | 1.3621533 | 4.8084 | 2.265557 | 2.22E-21 | 20.653 | Up | Amino acid metabolism |
| 5-dodecenoate (12:1n7) | 197.16 | 8.70 | negative | ESKD/NC | 1.3587233 | 0.45455 | -1.13749 | 1.93E-21 | 20.715 | Down | Fatty acid metabolism |
| Calcitriol | 416.63 | 25.40 | negative | ESKD/NC | 1.3585727 | 0.22449 | -2.15528 | 9.58E-21 | 20.019 | Down | Vitamin D metabolism |
| Acetyl carnitine | 202.11 | 2.40 | negative | ESKD/NC | 1.3532585 | 0.15766 | -2.66511 | 6.67E-21 | 20.176 | Down | Fatty acid metabolism |
| Citric acid | 191.00 | 2.70 | negative | ESKD/NC | 1.3530642 | 6.0016 | 2.585347 | 1.43E-20 | 19.844 | Up | TCA cycle |
| Hippurate | 178.05 | 3.70 | negative | ESKD/NC | 1.3467615 | 3.0025 | 1.586164 | 9.27E-20 | 19.033 | Up | Amino acid metabolism |
| Lactate | 89.03 | 1.80 | negative | ESKD/NC | 1.337013 | 0.18 | -2.47393 | 3.33E-19 | 18.477 | Down | Glyclosis |
| Myristoleate | 225.18 | 5.70 | negative | ESKD/NC | 1.3329123 | 2.9371 | 1.554392 | 6.94E-19 | 18.159 | Up | Fatty acid metabolism |
| Cinnamic acid | 147.00 | 3.30 | negative | ESKD/NC | 1.3320322 | 5.5425 | 2.470537 | 1.14E-18 | 17.943 | Up | Phenylpropanoid metabolism |
| Citrulline | 174.09 | 2.00 | negative | ESKD/NC | 1.3182266 | 2.2903 | 1.195537 | 6.19E-18 | 17.208 | Up | Urea cycle |
| Phenylalanine | 164.07 | 5.20 | negative | ESKD/NC | 1.3181541 | 0.19067 | -2.39085 | 4.23E-18 | 17.374 | Down | Amino acid metabolism |
| Ornithine | 132.09 | 6.30 | negative | ESKD/NC | 1.3160491 | 0.2963 | -1.75487 | 1.94E-17 | 16.712 | Down | Urea cycle and AA metabolism |
| Cholesteryl sulphate | 465.35 | 7.10 | negative | ESKD/NC | 1.2988792 | 3.5632 | 1.833173 | 1.02E-15 | 14.99 | Up | Steroid biosynthesis |
| Succinate | 117.00 | 2.00 | negative | ESKD/NC | 1.2968461 | 3.4991 | 1.806984 | 6.00E-16 | 15.222 | Up | TCA cycle |
| Hypotaurine | 108.01 | 1.80 | negative | ESKD/NC | 1.2950233 | 2.7256 | 1.446574 | 2.49E-16 | 15.604 | Up | Taurine and hypotaurine metabolism |
| Adipate | 145.05 | 6.10 | negative | ESKD/NC | 1.2796801 | 3.0667 | 1.616687 | 4.86E-15 | 14.313 | Up | Fatty acid metabolism |
| Arginine | 175.10 | 2.60 | negative | ESKD/NC | 1.2791977 | 3.2 | 1.678072 | 1.02E-15 | 14.99 | Up | Amino acid metabolism |
| Malic Acid | 133.01 | 2.30 | negative | ESKD/NC | 1.2727681 | 4.0374 | 2.013427 | 2.06E-14 | 13.687 | Up | TCA cycle |
| Thymol sulfate | 229.05 | 7.35 | negative | ESKD/NC | 1.2672047 | 1.5038 | 0.588613 | 1.83E-14 | 13.738 | Up | Phase II metabolism |
| Deoxycholate | 391.28 | 12.10 | negative | ESKD/NC | 1.2629242 | 2.6154 | 1.387032 | 2.35E-14 | 13.629 | Up | Bile acid metabolism |
| Arachidonate | 303.20 | 16.26 | negative | ESKD/NC | 1.1598333 | 2.4591 | 1.29813 | 4.56E-14 | 13.341 | Up | Fatty acid metabolism |
| 3-hydroxydecanoate | 187.13 | 8.40 | negative | ESKD/NC | 1.1453329 | 0.665 | -0.58857 | 2.03E-13 | 12.692 | Down | Fatty acid metabolism |
| Phenol sulfate | 172.99 | 4.60 | negative | ESKD/NC | 1.140668 | 1.5002 | 0.585155 | 1.77E-13 | 12.751 | Up | Xenobiotic Metabolism |
| 16-hydroxypalmitate | 271.22 | 3.79 | negative | ESKD/NC | 1.1305962 | 0.668 | -0.58208 | 6.08E-13 | 12.216 | Down | Fatty acid metabolism |
| Thymidine | 241.08 | 0.68 | negative | ESKD/NC | 1.1209837 | 1.54 | 0.62293 | 2.37E-12 | 11.624 | Up | Pyrimidine metabolism |
| Asparagine | 131.05 | 3.90 | negative | ESKD/NC | 1.113624 | 2.1305 | 1.091192 | 4.36E-12 | 11.361 | Up | Amino acid metabolism |
| octadecanedioate (C18) | 313.24 | 8.40 | negative | ESKD/NC | 1.0874305 | 0.667 | -0.58424 | 2.19E-11 | 10.66 | Down | Fatty acid metabolism |
| Indoxyl sulphate | 212.23 | 6.20 | negative | ESKD/NC | 1.0829889 | 2.2286 | 1.156138 | 4.23E-11 | 10.374 | Up | Tryptophan metabolism, Uremic toxins (Gut microbiota) |
| N-formyl methionine | 176.00 | 6.94 | negative | ESKD/NC | 1.0669805 | 2.375 | 1.247928 | 1.19E-10 | 9.9258 | Up | Amino acid metabolism |
| 4-vinylphenol sulfate | 199.01 | 5.53 | negative | ESKD/NC | 1.0215587 | 1.5008 | 0.585732 | 2.11E-09 | 8.6749 | Up | Amino acid metabolism (Gut-microbiota) |
| Kynurenic acid | 188.04 | 5.90 | negative | ESKD/NC | 1.0180468 | 2.25 | 1.169925 | 3.48E-09 | 8.458 | Up | Tryptophan metabolism |
| Oleic acid | 281.20 | 11.50 | negative | ESKD/NC | 0.9898889 | 1.9839 | 0.988339 | 1.15E-08 | 7.941 | Up | Fatty acid metabolism |
| Lithocholate | 375.27 | 10.90 | negative | ESKD/NC | 0.9517941 | 1.9231 | 0.943434 | 7.89E-08 | 7.1031 | Up | Bile acid metabolism |
| p-cresyl Sulfate | 187.01 | 8.74 | negative | ESKD/NC | 0.9323259 | 1.5007 | 0.585636 | 2.14E-07 | 6.6687 | Up | Amino acid metabolism (Gut-microbiota) |
| Linoleic acid | 279.24 | 22.70 | negative | ESKD/NC | 0.9224483 | 2.3333 | 1.222372 | 2.03E-07 | 6.693 | Up | Fatty acid metabolism |
| Glutamate | 146.00 | 4.30 | negative | ESKD/NC | 0.918107 | 1.9742 | 0.981268 | 2.64E-07 | 6.5779 | Up | Amino acid metabolism |
| Stearic acid | 284.27 | 23.16 | negative | ESKD/NC | 0.9084094 | 1.5652 | 0.646347 | 5.21E-07 | 6.2832 | Up | Fatty acid metabolism |
| Aspartate | 132.00 | 2.10 | negative | ESKD/NC | 0.907694 | 1.9912 | 0.993638 | 4.49E-07 | 6.3479 | Up | Urea cycle, Amino acid metabolism, TCA Cycle |
| Ceramide | 580.49 | 19.46 | negative | ESKD/NC | 0.9011536 | 1.625 | 0.70044 | 5.92E-07 | 6.2279 | Up | Phospholipid metabolism |
| Pyruvate | 87.30 | 3.60 | negative | ESKD/NC | 0.8692223 | 1.9893 | 0.992261 | 2.15E-06 | 5.6679 | Up | TCA cycle |
| 17-methyloctadecanoic acid | 285.30 | 9.20 | negative | ESKD/NC | 0.8668763 | 1.8709 | 0.903732 | 1.96E-06 | 5.7084 | Up | Fatty acid metabolism |
| Xanthine | 151.03 | 1.70 | negative | ESKD/NC | 0.8609329 | 1.8006 | 0.848478 | 2.77E-06 | 5.5575 | Up | Purine metabolism |
| Homocysteine | 134.03 | 3.90 | negative | ESKD/NC | 0.8606111 | 1.8571 | 0.893052 | 3.30E-06 | 5.482 | Up | Amino acid metabolism |
| Nonadecanal | 283.30 | 9.10 | negative | ESKD/NC | 0.8179403 | 2.1321 | 1.092275 | 1.62E-05 | 4.7905 | Up | Fatty acid metabolism |
| 2-Keto Tridecanoic Acid | 213.00 | 5.60 | negative | ESKD/NC | 0.8052269 | 0.29834 | -1.74497 | 1.39E-05 | 4.8561 | Down | Fatty acid metabolism |
| Oleic acid-2,6-diisopropylanilide | 443.30 | 10.50 | negative | ESKD/NC | 0.7731108 | 2.0018 | 1.001298 | 3.87E-05 | 4.4119 | Up | Fatty acid metabolism |
| Nonadecanoate | 297.28 | 3.40 | negative | ESKD/NC | 0.7603518 | 2 | 1 | 5.21E-05 | 4.2832 | Up | Fatty acid metabolism |
| Eicosenoate | 309.30 | 10.10 | negative | ESKD/NC | 0.7601467 | 1.65 | 0.722466 | 6.05E-05 | 4.2186 | Up | Fatty acid metabolism |
| Myo-inositol | 179.05 | 2.90 | negative | ESKD/NC | 0.7221619 | 1.7143 | 0.77762 | 0.0001751 | 3.7567 | Up | Phospholipid metabolism |
| p-Hydroxyphenyllactic Acid | 181.10 | 3.50 | negative | ESKD vs eCKD | 1.6127762 | 4.9122 | 2.2964 | 4.30E-29 | 28.367 | Up | Amino acid metabolism |
| Glycochenodeoxycholate | 448.31 | 9.95 | negative | ESKD vs eCKD | 1.6091947 | 3.7 | 1.8875 | 2.78E-29 | 28.556 | Up | Bile acid metabolism |
| 5-dodecenoate (12:1n7) | 197.16 | 8.70 | negative | ESKD vs eCKD | 1.5959189 | 0.4 | -1.3219 | 2.13E-27 | 26.672 | Down | Fatty acid metabolism |
| Inosine | 267.07 | 1.23 | negative | ESKD vs eCKD | 1.59589 | 2.6 | 1.3785 | 4.89E-27 | 26.311 | Up | Purine metabolism |
| Creatinine | 112.10 | 6.90 | negative | ESKD vs eCKD | 1.5862772 | 2.9 | 1.5361 | 2.53E-26 | 25.597 | Up | Amino acid metabolism |
| Taurocholate | 514.28 | 6.67 | negative | ESKD vs eCKD | 1.5821172 | 2.5032 | 1.3238 | 1.23E-25 | 24.911 | Up | Bile acid metabolism |
| Ornithine | 132.09 | 6.30 | negative | ESKD vs eCKD | 1.5717513 | 0.25 | -2 | 2.66E-25 | 24.576 | Down | Urea cycle and AA metabolism |
| Acetyl carnitine | 202.11 | 2.40 | negative | ESKD vs eCKD | 1.5476628 | 0.17677 | -2.5001 | 1.71E-21 | 20.767 | Down | Fatty acid metabolism |
| Calcitriol | 416.63 | 25.40 | negative | ESKD vs eCKD | 1.5344894 | 0.22 | -2.1844 | 4.44E-20 | 19.353 | Down | Vitamin D metabolism |
| Indoxyl sulphate | 212.23 | 6.20 | negative | ESKD vs eCKD | 1.5145748 | 2.6897 | 1.4274 | 4.89E-19 | 18.311 | Up | Tryptophan metabolism, Uremic toxins (Gut microbiota) |
| Adipic acid | 145.05 | 6.10 | negative | ESKD vs eCKD | 1.4964185 | 2.6 | 1.3785 | 9.32E-18 | 17.031 | Up | Fatty acid metabolism |
| Asparagine | 131.05 | 3.90 | negative | ESKD vs eCKD | 1.4872972 | 3.1 | 1.6323 | 9.32E-18 | 17.031 | Up | Amino acid metabolism |
| Deoxycholate | 391.28 | 12.10 | negative | ESKD vs eCKD | 1.4843731 | 2.99 | 1.5802 | 9.17E-18 | 17.038 | Up | Bile acid metabolism |
| N-formyl methionine | 176.00 | 6.94 | negative | ESKD vs eCKD | 1.4750552 | 3.1 | 1.6323 | 2.95E-17 | 16.53 | Up | Amino acid metabolism |
| 3-hydroxydecanoate | 187.13 | 8.40 | negative | ESKD vs eCKD | 1.4743314 | 0.63 | -0.66658 | 7.99E-17 | 16.098 | Down | Fatty acid metabolism |
| Thymol sulfate | 229.05 | 7.35 | negative | ESKD vs eCKD | 1.4636058 | 1.5006 | 0.585539 | 5.27E-16 | 15.278 | Up | PhasII metabolism |
| Lactate | 89.025 | 1.80 | negative | ESKD vs eCKD | 1.4555179 | 0.21176 | -2.2395 | 8.86E-16 | 15.053 | Down | Glycolsis |
| Phenylalanine | 164.07 | 5.20 | negative | ESKD vs eCKD | 1.4208391 | 0.22 | -2.1844 | 1.10E-14 | 13.957 | Down | Amino acid metabolism |
| Kynurenic acid | 188.04 | 5.90 | negative | ESKD vs eCKD | 1.4154972 | 3.267 | 1.708 | 1.72E-14 | 13.764 | Up | Tryptophan metabolism |
| Lithocholate | 375.27 | 10.90 | negative | ESKD vs eCKD | 1.3843351 | 2.4 | 1.263 | 3.75E-13 | 12.426 | Up | Bile acid metabolism |
| Homocysteine | 134.03 | 3.90 | negative | ESKD vs eCKD | 1.3692602 | 2.2 | 1.1375 | 1.10E-12 | 11.957 | Up | Amino acid metabolism |
| Stearic acid | 284.27 | 23.16 | negative | ESKD vs eCKD | 1.3429043 | 1.86 | 0.89531 | 3.21E-12 | 11.493 | Up | Fatty acid metabolism |
| octadecanedioate (C18) | 313.24 | 8.40 | negative | ESKD vs eCKD | 1.3402165 | 0.64 | -0.64329 | 8.32E-12 | 11.08 | Down | Fatty acid metabolism |
| Oleic acid | 281.20 | 11.50 | negative | ESKD vs eCKD | 1.3262704 | 1.87 | 0.90304 | 1.11E-11 | 10.956 | Up | Fatty acid metabolism |
| 16-hydroxypalmitate | 271.22 | 3.79 | negative | ESKD vs eCKD | 1.3249104 | 0.65 | -0.61249 | 8.37E-12 | 11.077 | Down | Fatty acid metabolism |
| Pyruvate | 87.30 | 3.60 | negative | ESKD vs eCKD | 1.3111808 | 2.1484 | 1.1032 | 3.85E-11 | 10.414 | Up | TCA cycle |
| Phenol sulfate | 172.99 | 4.60 | negative | ESKD vs eCKD | 1.3100241 | 1.5001 | 0.585059 | 4.69E-11 | 10.329 | Up | Xenobiotic Metabolism |
| Thymidine | 241.08 | 0.68 | negative | ESKD vs eCKD | 1.0477466 | 1.5009 | 0.585828 | 9.23E-10 | 9.035 | Up | Pyrimidine metabolism |
| Arginine | 175.10 | 2.60 | negative | ESKD vs eCKD | 1.0373099 | 1.6 | 0.67807 | 1.07E-09 | 8.9696 | Up | Amino acid metabolism |
| Succinate | 117.00 | 2.00 | negative | ESKD vs eCKD | 0.9816832 | 0.6 | -0.73697 | 2.80E-09 | 8.5525 | Down | TCA cycle |
| Myo-inositol | 179.05 | 2.90 | negative | ESKD vs eCKD | 0.950966 | 1.81 | 0.85599 | 1.24E-07 | 6.9062 | Up | Phospholipid metabolism |
| Linoleic acid | 279.24 | 22.70 | negative | ESKD vs eCKD | 0.9327603 | 2.0924 | 1.0651 | 2.62E-06 | 5.5825 | Up | Fatty acid metabolism |
| Arachidonate | 303.20 | 16.26 | negative | ESKD vs eCKD | 0.9305394 | 1.51 | 0.59455 | 3.75E-06 | 5.4255 | Up | Fatty acid metabolism |
| Phenylacetylglutamine | 265.12 | 8.07 | positive | eCKD/NC | 1.4780244 | 10.912 | 3.447844 | 2.24E-42 | 41.65 | Up | Amino acid metabolism |
| Ascorbate | 177.10 | 1.20 | positive | eCKD/NC | 1.4633839 | 0.19285 | -2.37445 | 1.67E-34 | 33.778 | Down | Vitamin C metabolism |
| Lactose | 343.12 | 0.73 | positive | eCKD/NC | 1.451943 | 4.7806 | 2.257192 | 3.46E-30 | 29.461 | Up | Galactose metabolism |
| Dimethylarginine | 203.14 | 9.20 | positive | eCKD/NC | 1.4494015 | 6.2 | 2.632268 | 2.82E-29 | 28.55 | Up | Amino acid metabolism |
| Pseudouridine | 245.08 | 5.80 | positive | eCKD/NC | 1.4493349 | 3.25 | 1.70044 | 5.17E-30 | 29.287 | Up | RNA metabolism |
| Methioninesulfoxide | 166.05 | 0.76 | positive | eCKD/NC | 1.4491765 | 2.7985 | 1.484654 | 1.62E-29 | 28.789 | Up | Amino acid metabolism |
| Palmitic acid | 255.23 | 9.50 | positive | eCKD/NC | 1.449092 | 5.9502 | 2.572938 | 5.73E-30 | 29.242 | Up | Fatty acid metabolism |
| Palmitoylcarnitine | 400.34 | 14.90 | positive | eCKD/NC | 1.439901 | 4.6149 | 2.206299 | 5.01E-27 | 26.3 | Up | Fatty acid metabolism |
| Allantoin | 159.06 | 1.56 | positive | eCKD/NC | 1.4353392 | 5.0857 | 2.346446 | 5.30E-27 | 26.276 | Up | Urea cycle |
| 4-pyridoxate | 184.06 | 0.71 | positive | eCKD/NC | 1.4351825 | 3.1347 | 1.648327 | 3.11E-27 | 26.507 | Up | Vitamin B6 metabolism |
| Lysophosphatidylcholine (18:1) | 522.36 | 5.80 | positive | eCKD/NC | 1.4307706 | 0.52853 | -0.91994 | 1.36E-25 | 24.866 | Down | Phospholipid metabolism |
| Docosahexaenoic acid | 329.25 | 17.76 | positive | eCKD/NC | 1.4302767 | 0.21773 | -2.19939 | 3.22E-26 | 25.493 | Down | Fatty acid metabolism |
| Urea | 61.03 | 2.70 | positive | eCKD/NC | 1.4280918 | 1.6667 | 0.736994 | 1.97E-25 | 24.706 | Up | Urea cycle |
| Dimethylglycine | 104.07 | 1.87 | positive | eCKD/NC | 1.415206 | 4.9108 | 2.295958 | 2.20E-23 | 22.657 | Up | Amino acid metabolism |
| N-Acetylneuraminic Acid | 310.12 | 1.20 | positive | eCKD/NC | 1.4109701 | 2.6471 | 1.404413 | 5.06E-23 | 22.296 | Up | Sialic acid metabolism |
| Theobromine | 181.07 | 1.72 | positive | eCKD/NC | 1.3960306 | 0.48 | -1.05889 | 1.32E-21 | 20.881 | Down | Purine metabolism |
| Trimethylamine N-Oxide | 76.08 | 0.85 | positive | eCKD/NC | 1.3872137 | 3.7255 | 1.897434 | 2.10E-20 | 19.677 | Up | Choline metabolism (Gut microbiota) |
| Cystine | 241.03 | 2.46 | positive | eCKD/NC | 1.3827532 | 1.7989 | 0.847115 | 2.59E-20 | 19.587 | Up | Amino acid metabolism |
| Betaine | 118.09 | 1.80 | positive | eCKD/NC | 1.3801736 | 0.5 | -1 | 1.75E-19 | 18.758 | Down | One-carbon metabolism and amino acid metabolism |
| Porphobilinogen | 227.1 | 6.88 | positive | eCKD/NC | 1.3757642 | 1.5192 | 0.603312 | 9.02E-20 | 19.045 | Up | Porphyrin metabolism |
| Xanthosine | 285.08 | 1.13 | positive | eCKD/NC | 1.3743353 | 2.4991 | 1.321409 | 1.31E-19 | 18.882 | Up | Purine metabolism |
| Creatinine | 114.06 | 0.62 | positive | eCKD/NC | 1.3695591 | 8.3148 | 3.055682 | 5.90E-19 | 18.229 | Up | Amino acid metabolism |
| Glycerophosphoryl choline | 258.10 | 12.50 | positive | eCKD/NC | 1.367152 | 1.7857 | 0.83649 | 7.88E-19 | 18.103 | Up | Phospholipid metabolism |
| Homoarginine | 189.12 | 9.39 | positive | eCKD/NC | 1.3668149 | 2.0401 | 1.02864 | 6.74E-19 | 18.171 | Up | Amino acid metabolism |
| 3-Phenylpropionate | 149.06 | 4.70 | positive | eCKD/NC | 1.3568934 | 1.5007 | 0.585636 | 2.53E-18 | 17.597 | Up | Amino acid metabolism |
| Glycyl-tyrosine | 239.11 | 8.03 | positive | eCKD/NC | 1.3533781 | 1.6701 | 0.739934 | 3.66E-18 | 17.437 | Up | Amino acid metabolism |
| Threonine | 120.07 | 0.78 | positive | eCKD/NC | 1.3442868 | 2.6008 | 1.378955 | 1.01E-17 | 16.996 | Up | Amino acid metabolism |
| Indolepropionate | 190.09 | 6.68 | positive | eCKD/NC | 1.3403318 | 1.5907 | 0.669662 | 7.11E-17 | 16.148 | Up | Tryptophan metabolism |
| Argininosuccinic Acid | 291.13 | 1.90 | positive | eCKD/NC | 1.3368891 | 8.2506 | 3.044499 | 6.20E-17 | 16.208 | Up | Urea cycle |
| Sphingomyelin | 731.60 | 22.42 | positive | eCKD/NC | 1.3350615 | 1.9102 | 0.933724 | 8.49E-17 | 16.071 | Up | Phospholipid metabolism |
| Butyrylcarnitine | 232.15 | 2.69 | positive | eCKD/NC | 1.3346088 | 0.51282 | -0.96348 | 4.80E-17 | 16.319 | Down | Fatty acid metabolism |
| Taurine | 126.02 | 0.72 | positive | eCKD/NC | 1.3339418 | 2.1333 | 1.093087 | 1.68E-17 | 16.776 | Up | Amino acid metabolism |
| N-acetylornithine | 175.12 | 0.71 | positive | eCKD/NC | 1.333288 | 1.64 | 0.713696 | 4.33E-17 | 16.364 | Up | Amino acid metabolism |
| Suberate | 173.81 | 3.18 | positive | eCKD/NC | 1.3195474 | 3.8754 | 1.954345 | 8.33E-16 | 15.079 | Up | Fatty acid metabolism |
| Lysyl-glutamate | 371.63 | 5.51 | positive | eCKD/NC | 1.3053425 | 0.669 | -0.57992 | 4.32E-15 | 14.365 | Down | Amino acid metabolism |
| Cortisol | 363.22 | 7.70 | positive | eCKD/NC | 1.2773888 | 0.5349 | -0.90266 | 1.21E-13 | 12.917 | Down | Steroid biosynthesis |
| Dihydrobiopterin | 240.11 | 1.05 | positive | eCKD/NC | 1.2767864 | 2.9245 | 1.54819 | 6.42E-14 | 13.192 | Up | Tetrahydrobiopterin biosynthesis |
| Adenine | 136.05 | 2.25 | positive | eCKD/NC | 1.2753233 | 1.7049 | 0.769687 | 7.10E-14 | 13.148 | Up | Purine metabolism |
| Uric acid | 169.04 | 3.94 | positive | eCKD/NC | 1.2605788 | 17.429 | 4.123418 | 4.79E-13 | 12.319 | Up | Purine metabolism |
| Methylmalonate | 117.02 | 3.10 | positive | eCKD/NC | 1.2536556 | 2.8484 | 1.510152 | 3.11E-13 | 12.507 | Up | Fatty acid metabolism |
| 3-methylhistidine | 170.09 | 0.89 | positive | eCKD/NC | 1.2191121 | 2.6748 | 1.419431 | 6.42E-12 | 11.193 | Up | Amino acid metabolism |
| N6-Methyllysine | 161.13 | 9.88 | positive | eCKD/NC | 1.1914379 | 1.5001 | 0.585059 | 5.01E-11 | 10.3 | Up | Amino acid metabolism |
| Dihydrocapsaicin | 308.22 | 2.20 | positive | eCKD/NC | 1.1838671 | 0.668 | -0.58208 | 1.14E-10 | 9.9417 | Down | Phenylpropanoid Metabolism |
| 5-oxoproline | 130.05 | 0.69 | positive | eCKD/NC | 1.1580716 | 1.5003 | 0.585251 | 6.16E-10 | 9.2104 | Up | Amino acid metabolism |
| γ-glutamyl-phenylalanine | 295.13 | 3.28 | positive | eCKD/NC | 1.1412018 | 1.509 | 0.593593 | 7.97E-10 | 9.0986 | Up | Amino acid metabolism |
| Anthranilate | 120.04 | 8.62 | positive | eCKD/NC | 1.0729532 | 1.8 | 0.847997 | 3.50E-08 | 7.4555 | Up | Tryptophan metabolism |
| Hydroxyproline | 132.10 | 7.90 | positive | eCKD/NC | 1.0433364 | 1.7936 | 0.842858 | 1.01E-07 | 6.9936 | Up | Amino acid metabolism |
| p-cresyl Sulfate | 187.00 | 6.20 | positive | eCKD/NC | 0.7015465 | 1.5008 | 0.585732 | 0.0011702 | 2.9318 | Up | Amino acid metabolism (Gut microbiota) |
| Kynurenic acid | 190.05 | 5.90 | positive | eCKD/NC | 0.5661888 | 0.665 | -0.58857 | 0.013143 | 1.8813 | Down | Tryptophan metabolism |
| Alpha-Ketoglutarate | 145.01 | 2.30 | positive | eCKD/NC | 0.4743072 | 0.666 | -0.58641 | 0.035049 | 1.4553 | Down | TCA cycle and amino acid metabolism |
| Phenylacetylglutamine | 265.12 | 8.07 | positive | ESKD/NC | 1.2973126 | 12.088 | 3.595504 | 1.33E-44 | 43.875 | Up | Amino acid metabolism |
| 3-hydroxyanthranilate | 154.05 | 5.42 | positive | ESKD/NC | 1.2885691 | 3.472 | 1.795767 | 1.63E-35 | 34.789 | Up | Tryptophan metabolism |
| Trimethylamine N-Oxide | 76.08 | 0.85 | positive | ESKD/NC | 1.2885082 | 15.275 | 3.9331 | 1.80E-36 | 35.746 | Up | Choline metabolism (Gut microbiota) |
| Methylguanidine | 74.071 | 6.88 | positive | ESKD/NC | 1.2879215 | 4.36 | 2.124328 | 2.61E-34 | 33.583 | Up | Amino acid metabolism |
| Glycyl-proline | 173.09 | 6.54 | positive | ESKD/NC | 1.286554 | 2.3884 | 1.256044 | 7.57E-34 | 33.121 | Up | Amino acid metabolism |
| Lysyl-proline | 355.65 | 6.58 | positive | ESKD/NC | 1.2863737 | 0.55484 | -0.84986 | 1.64E-33 | 32.784 | Down | Amino acid metabolism |
| Lysophosphatidylcholine (18:1) | 522.36 | 5.80 | positive | ESKD/NC | 1.2852267 | 0.34483 | -1.53604 | 1.64E-33 | 32.784 | Down | Phospholipid metabolism |
| Creatinine | 114.06 | 6.90 | positive | ESKD/NC | 1.2845882 | 24.113 | 4.591739 | 1.64E-33 | 32.784 | Up | Amino acid metabolism |
| Ascorbate | 177.10 | 1.20 | positive | ESKD/NC | 1.2823553 | 0.18715 | -2.41773 | 1.64E-33 | 32.784 | Down | Vitamin C metabolism |
| Anthranilate | 120.04 | 8.62 | positive | ESKD/NC | 1.2818919 | 4.86 | 2.280956 | 3.95E-32 | 31.403 | Up | Tryptophan metabolism |
| Cortisol | 363.22 | 7.70 | positive | ESKD/NC | 1.2783801 | 0.31772 | -1.65417 | 5.21E-31 | 30.283 | Down | Steroid biosynthesis |
| Threonine | 120.07 | 0.78 | positive | ESKD/NC | 1.2753861 | 16.68 | 4.060047 | 3.18E-29 | 28.498 | Up | Amino acid metabolism |
| Pipecolate | 130.09 | 1.19 | positive | ESKD/NC | 1.2720357 | 0.37766 | -1.40484 | 1.30E-28 | 27.887 | Down | Amino acid metabolism |
| Lactose | 343.12 | 0.73 | positive | ESKD/NC | 1.2701226 | 5.0777 | 2.344175 | 3.05E-28 | 27.516 | Up | Galactose metabolism |
| Glycyl-valine | 175.11 | 2.33 | positive | ESKD/NC | 1.2694524 | 2.4719 | 1.30562 | 4.59E-28 | 27.338 | Up | Amino acid metabolism |
| Dimethylglycine | 104.07 | 1.87 | positive | ESKD/NC | 1.2691707 | 5.3774 | 2.426909 | 1.04E-27 | 26.983 | Up | Amino acid metabolism |
| N-hydroxy-valine | 134.08 | 1.59 | positive | ESKD/NC | 1.2685529 | 0.39806 | -1.32894 | 4.59E-28 | 27.339 | Down | Amino acid metabolism |
| Taurine | 126.02 | 0.72 | positive | ESKD/NC | 1.2680287 | 4.2667 | 2.093121 | 6.39E-28 | 27.194 | Up | Amino acid metabolism |
| Pseudouridine | 245.08 | 5.80 | positive | ESKD/NC | 1.2669338 | 7.15 | 2.837943 | 3.81E-27 | 26.419 | Up | RNA metabolism |
| Xanthurenic acid | 206.04 | 2.39 | positive | ESKD/NC | 1.2660515 | 2.9967 | 1.583375 | 1.94E-27 | 26.713 | Up | Tryptophan metabolism |
| 4-pyridoxate | 184.06 | 0.71 | positive | ESKD/NC | 1.2656516 | 3.2653 | 1.707216 | 1.36E-26 | 25.866 | Up | Vitamin B6 metabolism |
| Biliverdin | 583.25 | 1.99 | positive | ESKD/NC | 1.2654982 | 0.3069 | -1.70416 | 1.66E-26 | 25.78 | Down | Porphyrin metabolism |
| Porphobilinogen | 227.10 | 6.88 | positive | ESKD/NC | 1.2650522 | 1.7223 | 0.784336 | 1.41E-26 | 25.852 | Up | Porphyrin metabolism |
| Theobromine | 181.07 | 1.72 | positive | ESKD/NC | 1.2642201 | 0.38 | -1.39593 | 1.41E-26 | 25.852 | Down | Purine metabolism |
| Urea | 61.03 | 2.70 | positive | ESKD/NC | 1.2640836 | 5.3333 | 2.415028 | 1.22E-26 | 25.913 | Up | Urea cycle |
| Docosahexaenoic acid | 329.25 | 17.76 | positive | ESKD/NC | 1.2637778 | 0.1828 | -2.45166 | 6.54E-27 | 26.184 | Down | Fatty acid metabolism |
| Paraxanthine | 181.07 | 2.32 | positive | ESKD/NC | 1.2636929 | 0.38 | -1.39593 | 1.70E-26 | 25.77 | Down | Urea cycle and Purine metabolism |
| Betaine | 118.09 | 1.80 | positive | ESKD/NC | 1.2635195 | 0.225 | -2.152 | 1.11E-26 | 25.955 | Down | One-carbon metabolism and amino acid metabolism |
| Homogentisate | 169.05 | 10.38 | positive | ESKD/NC | 1.2617361 | 2.6316 | 1.39594 | 2.74E-26 | 25.562 | Up | Amino acid metabolism |
| Argininosuccinic Acid | 291.13 | 1.90 | positive | ESKD/NC | 1.2610681 | 9.1879 | 3.199735 | 4.37E-26 | 25.36 | Up | Urea cycle |
| Palmitic acid | 255.23 | 9.50 | positive | ESKD/NC | 1.2603673 | 6.4211 | 2.68282 | 5.45E-26 | 25.264 | Up | Fatty acid metabolism |
| Palmitoylcarnitine | 400.34 | 14.90 | positive | ESKD/NC | 1.2597711 | 4.9852 | 2.317651 | 6.84E-26 | 25.165 | Up | Fatty acid metabolism |
| Adenine | 136.05 | 2.25 | positive | ESKD/NC | 1.2596325 | 2.8852 | 1.528671 | 4.07E-26 | 25.391 | Up | Purine metabolism |
| Methylmalonate | 117.02 | 3.10 | positive | ESKD/NC | 1.258982 | 14.551 | 3.863046 | 9.96E-26 | 25.002 | Up | Fatty acid metabolism |
| Allantoin | 159.06 | 1.56 | positive | ESKD/NC | 1.2562028 | 5.7143 | 2.514577 | 5.80E-26 | 25.236 | Up | Urea cycle |
| Valine | 118.09 | 0.79 | positive | ESKD/NC | 1.2558544 | 0.45778 | -1.12727 | 4.13E-25 | 24.384 | Down | Amino acid metabolism |
| Aspartyl-phenylalanine | 281.11 | 5.93 | positive | ESKD/NC | 1.255642 | 2.551 | 1.351063 | 5.77E-25 | 24.239 | Up | Amino acid metabolism |
| Glycerophosphoryl choline | 258.10 | 12.50 | positive | ESKD/NC | 1.2550622 | 2.5 | 1.321928 | 2.27E-25 | 24.644 | Up | Phospholipid metabolism |
| Suberate | 173.81 | 3.18 | positive | ESKD/NC | 1.2532917 | 7.3246 | 2.87275 | 8.10E-25 | 24.092 | Up | Fatty acid metabolism |
| 2-phenylglycine | 152.08 | 5.50 | positive | ESKD/NC | 1.2532734 | 2.58 | 1.367371 | 7.79E-25 | 24.108 | Up | Amino acid metabolism |
| 5-hydroxyindoleacetic acid | 192.07 | 4.11 | positive | ESKD/NC | 1.2506098 | 3.3677 | 1.751764 | 4.60E-24 | 23.337 | Up | Tryptophan metabolism (Gut microbiota) |
| Methionine sulfoxide | 166.05 | 0.76 | positive | ESKD/NC | 1.2486031 | 4.2015 | 2.070904 | 1.88E-23 | 22.726 | Up | Amino acid metabolism |
| N-Acetylneuraminic Acid | 310.12 | 1.20 | positive | ESKD/NC | 1.2475504 | 15.353 | 3.940449 | 1.21E-23 | 22.917 | Up | Sialic acid metabolism |
| Cortisone | 361.20 | 8.76 | positive | ESKD/NC | 1.2456528 | 0.6 | -0.73697 | 3.65E-23 | 22.438 | Down | Steroid biosynthesis |
| Glutamine | 147.08 | 8.44 | positive | ESKD/NC | 1.24328 | 3.3333 | 1.736951 | 5.09E-23 | 22.293 | Up | Amino acid metabolism |
| 3-Phenylpropionate | 149.06 | 4.70 | positive | ESKD/NC | 1.2424916 | 3.5897 | 1.843863 | 3.65E-23 | 22.438 | Up | Amino acid metabolism |
| Glutamyl-valine | 247.13 | 24.00 | positive | ESKD/NC | 1.2369326 | 2.5389 | 1.344204 | 3.41E-22 | 21.467 | Up | Amino acid metabolism |
| Prolyl-leucine | 229.16 | 2.69 | positive | ESKD/NC | 1.2367002 | 0.54819 | -0.86725 | 2.16E-22 | 21.667 | Down | Amino acid metabolism |
| Butyrylcarnitine | 232.15 | 2.69 | positive | ESKD/NC | 1.2357658 | 0.4 | -1.32193 | 7.39E-22 | 21.131 | Down | Fatty acid metabolism |
| Xanthosine | 285.08 | 1.13 | positive | ESKD/NC | 1.2274939 | 2.7083 | 1.437388 | 1.17E-21 | 20.934 | Up | Purine metabolism |
| 3-methylhistidine | 170.09 | 0.89 | positive | ESKD/NC | 1.2263039 | 2.9241 | 1.547993 | 2.82E-20 | 19.549 | Up | Amino acid metabolism |
| Theophylline | 179.06 | 1.01 | positive | ESKD/NC | 1.2195201 | 0.59 | -0.76121 | 6.94E-20 | 19.158 | Down | Purine metabolism |
| Dimethylarginine | 203.14 | 9.20 | positive | ESKD/NC | 1.2192158 | 4 | 2 | 4.58E-20 | 19.339 | Up | Amino acid metabolism |
| Cystine | 241.03 | 2.46 | positive | ESKD/NC | 1.2190022 | 2.9011 | 1.5366 | 3.95E-20 | 19.403 | Up | Amino acid metabolism |
| Glycocholate | 466.32 | 10.55 | positive | ESKD/NC | 1.1963345 | 7.6667 | 2.938606 | 1.57E-19 | 18.805 | Up | Bile acid metabolism |
| Norleucine | 132.09 | 1.29 | positive | ESKD/NC | 1.1854673 | 0.62813 | -0.67086 | 7.56E-19 | 18.121 | Down | Amino acid metabolism |
| N6-Methyllysine | 161.13 | 9.88 | positive | ESKD/NC | 1.1834916 | 1.6491 | 0.721679 | 2.31E-18 | 17.637 | Up | Amino acid metabolism |
| Glycyl-tyrosine | 239.11 | 8.03 | positive | ESKD/NC | 1.1820199 | 1.74 | 0.799087 | 4.57E-18 | 17.34 | Up | Amino acid metabolism |
| Lysyl-glutamate | 371.63 | 5.51 | positive | ESKD/NC | 1.1811614 | 0.65022 | -0.621 | 5.11E-17 | 16.291 | Down | Amino acid metabolism |
| Hydroquinone | 110.11 | 4.28 | positive | ESKD/NC | 1.179409 | 1.7755 | 0.828225 | 1.07E-16 | 15.973 | Up | Xenobiotic Metabolism |
| Tyrosine | 182.08 | 1.75 | positive | ESKD/NC | 1.177694 | 0.59447 | -0.75032 | 2.73E-16 | 15.563 | Down | Amino acid metabolism |
| Dihydrocapsaicin | 308.22 | 2.20 | positive | ESKD/NC | 1.1754123 | 0.5177 | -0.94981 | 3.03E-16 | 15.519 | Down | Phenylpropanoid Metabolism |
| Dihydrobiopterin | 240.11 | 1.05 | positive | ESKD/NC | 1.1747247 | 3.0755 | 1.620821 | 4.69E-16 | 15.328 | Up | Tetrahydrobiopterin biosynthesis |
| Serine | 106.05 | 7.68 | positive | ESKD/NC | 1.1708115 | 0.6303 | -0.66589 | 3.56E-16 | 15.449 | Down | Amino acid metabolism |
| Sphingomyelin | 731.60 | 22.42 | positive | ESKD/NC | 1.170685 | 2.0322 | 1.023042 | 4.25E-16 | 15.372 | Up | Phospholipid metabolism |
| Diethanolamine | 106.09 | 0.96 | positive | ESKD/NC | 1.1702447 | 0.665 | -0.58857 | 4.19E-16 | 15.378 | Down | Phospholipid metabolism |
| Tryptophan | 205.10 | 7.00 | positive | ESKD/NC | 1.1515707 | 0.14634 | -2.7726 | 6.94E-15 | 14.159 | Down | Tryptophan metabolism |
| 1-aminobutyrate | 104.08 | 0.64 | positive | ESKD/NC | 1.1495096 | 0.668 | -0.58208 | 1.13E-14 | 13.946 | Down | Amino acid metabolism |
| N-acetylornithine | 175.12 | 0.71 | positive | ESKD/NC | 1.1485858 | 1.5006 | 0.585539 | 1.71E-14 | 13.768 | Up | Amino acid metabolism |
| Prolyl-lysine | 355.64 | 6.85 | positive | ESKD/NC | 1.1415561 | 0.64286 | -0.63742 | 2.98E-14 | 13.526 | Down | Amino acid metabolism |
| Cytosine | 112.05 | 1.05 | positive | ESKD/NC | 1.1386423 | 1.632 | 0.706641 | 6.71E-14 | 13.173 | Up | Pyrimidine metabolism |
| Prolyl-tyrosine | 279.13 | 8.28 | positive | ESKD/NC | 1.1302647 | 0.6699 | -0.57798 | 1.40E-13 | 12.855 | Down | Amino acid metabolism |
| Uric acid | 169.04 | 3.94 | positive | ESKD/NC | 1.1156478 | 23.714 | 4.567667 | 6.63E-13 | 12.178 | Up | Purine metabolism |
| Histidine | 156.08 | 9.06 | positive | ESKD/NC | 1.0441834 | 0.667 | -0.58424 | 3.30E-10 | 9.481 | Down | Amino acid metabolism |
| N-acetylglutamine | 189.19 | 1.50 | positive | ESKD/NC | 1.0375631 | 1.5135 | 0.597889 | 5.21E-10 | 9.2831 | Up | Amino acid metabolism |
| Beta-Hydroxybutyrate | 103.04 | 3.10 | positive | ESKD/NC | 1.0369029 | 1.5008 | 0.585732 | 4.25E-10 | 9.3713 | Up | Fatty acid metabolism |
| Kynurenic acid | 190.05 | 5.90 | positive | ESKD/NC | 1.0088832 | 2.25 | 1.169925 | 2.59E-09 | 8.587 | Up | Tryptophan metabolism |
| 5-oxoproline | 130.05 | 0.69 | positive | ESKD/NC | 1.0015763 | 1.5001 | 0.585059 | 3.58E-09 | 8.4458 | Up | Amino acid metabolism |
| Acetone | 59.05 | 1.30 | positive | ESKD/NC | 0.9660229 | 1.5284 | 0.612022 | 4.24E-08 | 7.3723 | Up | Fatty acid metabolism |
| 3-methoxytyrosine | 213.10 | 2.27 | positive | ESKD/NC | 0.9624312 | 1.5002 | 0.585155 | 5.08E-08 | 7.2939 | Up | Amino acid metabolism |
| Hydroxyproline | 132.10 | 7.90 | positive | ESKD/NC | 0.9298678 | 1.95 | 0.963474 | 2.68E-07 | 6.5713 | Up | Amino acid metabolism |
| p-cresyl Sulfate | 187.00 | 6.20 | positive | ESKD/NC | 0.8981003 | 1.783 | 0.834307 | 7.82E-07 | 6.1067 | Up | Amino acid metabolism (Gut microbiota) |
| Orotate | 157.00 | 5.40 | positive | ESKD/NC | 0.7304482 | 1.5 | 0.584963 | 0.0002379 | 3.6237 | Up | Pyrimidine metabolism |
| Alpha-Ketoglutarate | 145.01 | 2.30 | positive | ESKD/NC | 0.6947291 | 0.5009 | -0.99741 | 0.0007411 | 3.1301 | Down | TCA cycle and amino acid metabolism |
| Pantothenic Acid | 220.12 | 6.60 | positive | ESKD/NC | 0.6864232 | 0.667 | -0.58424 | 0.0010026 | 2.9989 | Down | CoA biosynthesis |
| Lysyl-proline | 355.65 | 6.58 | positive | ESKD vs eCKD | 1.4597462 | 0.54 | -0.88897 | 8.16E-43 | 42.088 | Down | Amino acid metabolism |
| Methylguanidine | 74.071 | 6.88 | positive | ESKD vs eCKD | 1.4579652 | 3.3538 | 1.7458 | 5.30E-41 | 40.276 | Up | Amino acid metabolism |
| 3-hydroxyanthranilate | 154.05 | 5.42 | positive | ESKD vs eCKD | 1.4532719 | 3.1089 | 1.6364 | 4.65E-38 | 37.333 | Up | Tryptophan metabolism |
| Glycyl-proline | 173.09 | 6.54 | positive | ESKD vs eCKD | 1.4496856 | 2.3 | 1.2016 | 1.26E-36 | 35.898 | Up | Amino acid metabolism |
| Trimethylamine N-Oxide | 76.08 | 0.85 | positive | ESKD vs eCKD | 1.4490766 | 4.1 | 2.0356 | 3.78E-36 | 35.422 | Up | Choline metabolism (Gut microbiota) |
| Threonine | 120.07 | 0.78 | positive | ESKD vs eCKD | 1.4349691 | 6.4134 | 2.6811 | 2.49E-31 | 30.603 | Up | Amino acid metabolism |
| Xanthurenic acid | 206.04 | 2.39 | positive | ESKD vs eCKD | 1.4305242 | 3.1 | 1.6323 | 1.22E-29 | 28.913 | Up | Tryptophan metabolism |
| Anthranilate | 120.04 | 8.62 | positive | ESKD vs eCKD | 1.4290666 | 2.7 | 1.433 | 5.16E-30 | 29.288 | Up | Tryptophan metabolism |
| Pipecolate | 130.09 | 1.19 | positive | ESKD vs eCKD | 1.4282769 | 0.39 | -1.3585 | 1.24E-29 | 28.906 | Down | Amino acid metabolism |
| Homogentisate | 169.05 | 10.38 | positive | ESKD vs eCKD | 1.4185655 | 2.5 | 1.3219 | 2.29E-28 | 27.639 | Up | Amino acid metabolism |
| Biliverdin | 583.25 | 1.99 | positive | ESKD vs eCKD | 1.4165943 | 0.32692 | -1.613 | 1.01E-27 | 26.996 | Down | Porphyrin metabolism |
| N-hydroxy-valine | 134.08 | 1.59 | positive | ESKD vs eCKD | 1.4155365 | 0.40981 | -1.287 | 1.41E-27 | 26.852 | Down | Amino acid metabolism |
| Urea | 61.03 | 2.70 | positive | ESKD vs eCKD | 1.4155236 | 3.2 | 1.6781 | 1.97E-27 | 26.706 | Up | Urea cycle |
| Aspartyl-phenylalanine | 281.11 | 5.93 | positive | ESKD vs eCKD | 1.4151434 | 2.5 | 1.3219 | 9.54E-27 | 26.02 | Up | Amino acid metabolism |
| Glycyl-valine | 175.11 | 2.33 | positive | ESKD vs eCKD | 1.414926 | 2.2 | 1.1375 | 1.86E-27 | 26.73 | Up | Amino acid metabolism |
| 2-phenylglycine | 152.08 | 5.50 | positive | ESKD vs eCKD | 1.4107043 | 2.4 | 1.263034 | 5.85E-27 | 26.233 | Up | Amino acid metabolism |
| Creatinine | 114.06 | 6.90 | positive | ESKD vs eCKD | 1.4095342 | 2.9 | 1.5361 | 1.06E-26 | 25.973 | Up | Amino acid metabolism |
| 5-hydroxyindoleacetic acid | 192.07 | 4.11 | positive | ESKD vs eCKD | 1.4073261 | 3.24 | 1.696 | 1.83E-26 | 25.737 | Up | Tryptophan metabolism (Gut microbiota) |
| Methylmalonate | 117.02 | 3.10 | positive | ESKD vs eCKD | 1.4048312 | 5.1084 | 2.3529 | 5.44E-26 | 25.264 | Up | Fatty acid metabolism |
| N-Acetylneuraminic Acid | 310.12 | 1.20 | positive | ESKD vs eCKD | 1.3950363 | 5.8 | 2.5361 | 1.04E-24 | 23.984 | Up | Sialic acid metabolism |
| Glutamyl-valine | 247.13 | 24.00 | positive | ESKD vs eCKD | 1.3940137 | 2.4 | 1.263034 | 3.78E-24 | 23.422 | Up | Amino acid metabolism |
| Taurine | 126.02 | 0.72 | positive | ESKD vs eCKD | 1.3930521 | 2 | 1 | 3.16E-24 | 23.5 | Up | Amino acid metabolism |
| L-Glutamine | 147.08 | 8.44 | positive | ESKD vs eCKD | 1.3880703 | 3 | 1.585 | 2.82E-24 | 23.549 | Up | Amino acid metabolism |
| Prolyl-leucine | 229.16 | 2.69 | positive | ESKD vs eCKD | 1.3834345 | 0.58 | -0.78587 | 2.48E-23 | 22.606 | Down | Amino acid metabolism |
| Glycocholate | 466.32 | 10.55 | positive | ESKD vs eCKD | 1.3814238 | 8.5185 | 3.0906 | 9.68E-23 | 22.014 | Up | Bile acid metabolism |
| Valine | 118.09 | 0.79 | positive | ESKD vs eCKD | 1.3812835 | 0.49713 | -1.0083 | 1.22E-23 | 22.913 | Down | Amino acid metabolism |
| 3-Phenylpropionate | 149.06 | 4.70 | positive | ESKD vs eCKD | 1.3812545 | 2.4779 | 1.3091 | 4.83E-23 | 22.316 | Up | Amino acid metabolism |
| Homoarginine | 189.12 | 9.39 | positive | ESKD vs eCKD | 1.3759304 | 0.51 | -0.97143 | 1.56E-22 | 21.808 | Down | Amino acid metabolism |
| Pseudouridine | 245.08 | 5.80 | positive | ESKD vs eCKD | 1.374331 | 2.2 | 1.1375 | 4.15E-22 | 21.382 | Up | RNA metabolism |
| Tryptophan | 205.10 | 7.00 | positive | ESKD vs eCKD | 1.3713254 | 0.17647 | -2.5025 | 6.65E-22 | 21.177 | Down | Tryptophan metabolism |
| Adenine | 136.05 | 2.25 | positive | ESKD vs eCKD | 1.3606401 | 1.6923 | 0.75899 | 1.68E-21 | 20.775 | Up | Purine metabolism |
| Lysophosphatidylcholine (18:1) | 522.36 | 5.80 | positive | ESKD vs eCKD | 1.3527209 | 0.48 | -1.05889 | 5.59E-20 | 19.252 | Down | Phospholipid metabolism |
| Norleucine | 132.09 | 1.29 | positive | ESKD vs eCKD | 1.3363074 | 0.49 | -1.02915 | 2.88E-19 | 18.541 | Down | Amino acid metabolism |
| Indolepropionate | 190.09 | 6.68 | positive | ESKD vs eCKD | 1.3338142 | 0.55 | -0.8625 | 5.87E-19 | 18.232 | Down | Tryptophan metabolism (Gut microbiota) |
| Paraxanthine | 181.07 | 2.32 | positive | ESKD vs eCKD | 1.3294132 | 0.49351 | -1.0189 | 9.19E-19 | 18.037 | Down | Urea cycle and Purine metabolism |
| Hydroquinone | 110.11 | 4.28 | positive | ESKD vs eCKD | 1.3180903 | 2.14 | 1.097611 | 7.51E-18 | 17.125 | Up | Xenobiotic Metabolism |
| Prolyl-lysine | 355.64 | 6.85 | positive | ESKD vs eCKD | 1.3103161 | 0.56 | -0.8365 | 3.03E-17 | 16.518 | Down | Amino acid metabolism |
| Cortisone | 361.20 | 8.76 | positive | ESKD vs eCKD | 1.2909192 | 0.58 | -0.78588 | 3.14E-16 | 15.503 | Down | Steroid biosynthesis |
| Theophylline | 179.06 | 1.01 | positive | ESKD vs eCKD | 1.2729281 | 0.59 | -0.76121 | 1.65E-15 | 14.781 | Down | Purine metabolism |
| Serine | 106.05 | 7.68 | positive | ESKD vs eCKD | 1.2729096 | 0.6 | -0.73697 | 3.74E-15 | 14.427 | Down | Amino acid metabolism |
| Kynurenic acid | 190.05 | 5.90 | positive | ESKD vs eCKD | 1.2632188 | 3.2692 | 1.7089 | 1.11E-14 | 13.953 | Up | Tryptophan metabolism |
| Glycerophosphoryl choline | 258.10 | 12.50 | positive | ESKD vs eCKD | 1.2622699 | 1.98 | 0.992768 | 7.71E-15 | 14.113 | Up | Phospholipid metabolism |
| Suberate | 173.81 | 3.18 | positive | ESKD vs eCKD | 1.241056 | 1.89 | 0.91839 | 4.80E-14 | 13.319 | Up | Fatty acid metabolism |
| Cystine | 241.03 | 2.46 | positive | ESKD vs eCKD | 1.2348314 | 1.86 | 0.895303 | 8.27E-14 | 13.083 | Up | Amino acid metabolism |
| 1-aminobutyrate | 104.08 | 0.64 | positive | ESKD vs eCKD | 1.2315362 | 0.58 | -0.78588 | 1.55E-13 | 12.81 | Down | Amino acid metabolism |
| Methionine sulfoxide | 166.05 | 0.76 | positive | ESKD vs eCKD | 1.2311809 | 1.94 | 0.956057 | 7.25E-14 | 13.14 | Up | Amino acid metabolism |
| Dimethylarginine | 203.14 | 9.20 | positive | ESKD vs eCKD | 1.2246189 | 0.64516 | -0.63227 | 2.30E-13 | 12.638 | Down | Amino acid metabolism |
| Betaine | 118.09 | 1.80 | positive | ESKD vs eCKD | 1.0028031 | 0.45 | -1.152 | 5.39E-13 | 12.269 | Down | One-carbon metabolism and amino acid metabolism |
| Prolyl-tyrosine | 279.13 | 8.28 | positive | ESKD vs eCKD | 1.0022749 | 0.663 | -0.59292 | 5.43E-13 | 12.265 | Down | Amino acid metabolism |
| Cytosine | 112.05 | 1.05 | positive | ESKD vs eCKD | 0.983556 | 1.5002 | 0.585155 | 7.84E-11 | 10.106 | Up | Pyrimidine metabolism |
| Cortisol | 363.22 | 7.70 | positive | ESKD vs eCKD | 0.9663515 | 0.59397 | -0.75155 | 5.42E-07 | 6.2664 | Down | Steroid biosynthesis |
| Tyrosine | 182.08 | 1.75 | positive | ESKD vs eCKD | 0.9654002 | 0.6669 | -0.58446 | 1.46E-10 | 9.8351 | Down | Amino acid metabolism |
| Uric acid | 169.04 | 3.94 | positive | ESKD vs eCKD | 0.5172887 | 1.5001 | 0.585059 | 0.019585 | 1.7081 | Up | Purine metabolism |
| Alpha-Ketoglutarate | 145.01 | 2.30 | positive | ESKD vs eCKD | 0.4397596 | 0.669 | -0.57992 | 0.046891 | 1.3289 | Down | TCA cycle and amino acid metabolism |
