# Supplementary material for: Serum metabolomics identifies gut-derived uremic toxins and bile acid dysregulation associated with chronic kidney disease severity
Source: Sci Rep. 2026 Apr 14;16:12375. doi: 10.1038/s41598-026-44271-4 (PMC13083900; doi:10.1038/s41598-026-44271-4)
Supplement: Supplementary file 7 — Supplementary Material 7 [file 41598_2026_44271_MOESM7_ESM.docx]

**Table S7.** Interaction network analysis of significantly differential metabolites between eCKD and ESKD.

| **Id** | **Label** | **Degree** | **Betweenness** |
| --- | --- | --- | --- |
| C00022 | Pyruvic acid | 75 | 1376.65 |
| C00026 | Oxoglutaric acid | 70 | 959.02 |
| C00065 | L-Serine | 54 | 399.99 |
| C00062 | L-Arginine | 53 | 591.62 |
| C00064 | L-Glutamine | 48 | 281.34 |
| C00078 | L-Tryptophan | 47 | 248.63 |
| C00042 | Succinic acid | 45 | 287.46 |
| C00079 | L-Phenylalanine | 44 | 260.35 |
| C00077 | Ornithine | 43 | 502.66 |
| C00183 | L-Valine | 40 | 145.26 |
| C00188 | L-Threonine | 38 | 101.08 |
| C00147 | Adenine | 35 | 295.97 |
| C00082 | L-Tyrosine | 35 | 145.61 |
| C00002 | Adenosine triphosphate | 31 | 224.97 |
| C00712 | Oleic acid | 30 | 254.44 |
| C00719 | Betaine | 29 | 176.24 |
| C00007 | Oxygen | 27 | 176.69 |
| C00006 | NADP | 25 | 146.77 |
| C00008 | ADP | 25 | 130.16 |
| C00086 | Urea | 25 | 98.66 |
| C01330 | Sodium | 24 | 141.71 |
| C00116 | Glycerol | 24 | 114.74 |
| C00025 | L-Glutamic acid | 24 | 83.9 |
| C00152 | L-Asparagine | 24 | 23.14 |
| C00155 | Homocysteine | 23 | 119.18 |
| C00245 | Taurine | 23 | 85.58 |
| C00011 | Carbon dioxide | 23 | 66.79 |
|  | Hexacosanoic acid | 22 | 177.88 |
| C00108 | 2-Aminobenzoic acid | 22 | 41.74 |
| C00305 | Magnesium | 21 | 83.37 |
| C00219 | Arachidonic acid | 21 | 81.51 |
| C00004 | NADH | 21 | 66.8 |
| C00366 | Uric acid | 20 | 80.46 |
| C00294 | Inosine | 20 | 64.86 |
| C00020 | Adenosine monophosphate | 20 | 35.76 |
| C01933 | L-Norleucine | 18 | 78.21 |
| C00380 | Cytosine | 17 | 43.63 |
| C00010 | Coenzyme A | 17 | 35.44 |
| C00041 | L-Alanine | 17 | 25.14 |
| C00186 | L-Lactic acid | 17 | 20.81 |
| C00791 | Creatinine | 16 | 123.38 |
| C00037 | Glycine | 16 | 6.32 |
| C00214 | Thymidine | 15 | 63.9 |
| C00027 | Hydrogen peroxide | 15 | 38.18 |
| C00097 | L-Cysteine | 15 | 14.52 |
| C00073 | L-Methionine | 15 | 12.22 |
| C00044 | Guanosine triphosphate | 14 | 29.13 |
| C00084 | Acetaldehyde | 14 | 24.05 |
| C00021 | S-Adenosylhomocysteine | 14 | 23.39 |
| C00217 | D-Glutamic acid | 14 | 6.13 |
| C05635 | 5-Hydroxyindoleacetic acid | 13 | 51.14 |
| C00158 | Citric acid | 13 | 27.64 |
| C00148 | L-Proline | 13 | 4.8 |
| C01595 | Linoleic acid | 12 | 15.55 |
| C03878 | Beta-N-Acetylglucosamine | 12 | 15.12 |
| C00047 | L-Lysine | 12 | 13.58 |
| C00049 | L-Aspartic acid | 12 | 6.36 |
| C02170 | Methylmalonic acid | 11 | 32.77 |
| C04483 | Deoxycholic acid | 11 | 28.53 |
| C00018 | Pyridoxal 5'-phosphate | 11 | 20.42 |
| C00135 | L-Histidine | 11 | 1.33 |
| C00407 | L-Isoleucine | 11 | 0.89 |
| C00123 | L-Leucine | 11 | 0.89 |
| C00163 | Propionic acid | 10 | 21.57 |
| C00408 | Pipecolic acid | 10 | 20.79 |
| C00212 | Adenosine | 10 | 16.95 |
| C02356 | L-Alpha-aminobutyric acid | 10 | 15.53 |
| C00016 | FAD | 10 | 12.64 |
| C00067 | Formaldehyde | 10 | 6.71 |
| C00101 | Tetrahydrofolic acid | 10 | 6.58 |
| C00263 | L-Homoserine | 10 | 2.69 |
| C00780 | Serotonin | 9 | 20.95 |
| C00547 | Norepinephrine | 9 | 20.19 |
| C00035 | Guanosine diphosphate | 9 | 16.92 |
| C06104 | Adipic acid | 9 | 9.12 |
| C00383 | Malonic acid | 9 | 8.84 |
| C00036 | Oxalacetic acid | 9 | 8.3 |
| C00114 | Choline | 8 | 18.76 |
| C00788 | Epinephrine | 8 | 16.41 |
| C01104 | Trimethylamine N-oxide | 8 | 5.48 |
| C00334 | Gamma-Aminobutyric acid | 8 | 5.19 |
| C00048 | Glyoxylic acid | 8 | 4.78 |
| C00119 | Phosphoribosyl pyrophosphate | 8 | 4.57 |
| C00130 | Inosinic acid | 8 | 4.46 |
| C00099 | Beta-Alanine | 8 | 2.18 |
| C00134 | Putrescine | 8 | 1.45 |
| C02470 | Xanthurenic acid | 7 | 2.35 |
| C00735 | Hydrocortisone | 6 | 4.05 |
| C00632 | 3-Hydroxyanthranilic acid | 6 | 1.01 |
| C00504 | Folic acid | 5 | 7.5 |
| C00209 | Oxalic acid | 5 | 5.26 |
| C00440 | 5-Methyltetrahydrofolic acid | 5 | 2.28 |
| C00272 | Tetrahydrobiopterin | 5 | 2.18 |
| C00250 | Pyridoxal | 5 | 0.87 |
| C01717 | Kynurenic acid | 5 | 0.15 |
| C00451 | D-threo-Isocitric acid | 4 | 1.35 |
| C00601 | Phenylacetaldehyde | 4 | 0.85 |
| C00497 | D-Malic acid | 4 | 0.77 |
| C00170 | 5'-Methylthioadenosine | 4 | 0.71 |
| C02166 | Leukotriene C4 | 4 | 0.63 |
| C06314 | Lipoxin A4 | 4 | 0.63 |
| C00469 | Ethanol | 3 | 2.89 |
| C01607 | Phytanic acid | 3 | 1.81 |
| C00221 | Beta-D-Glucose | 3 | 1.73 |
| C00363 | dTDP | 3 | 1.56 |
| C05332 | Phenylethylamine | 3 | 1.53 |
| C03665 | 2-Aminoisobutyric acid | 3 | 1.53 |
| C06102 | Adipate semialdehyde | 3 | 1.51 |
| C01013 | Hydroxypropionic acid | 3 | 1.49 |
| C00355 | L-Dopa | 3 | 1.48 |
| C00189 | Ethanolamine | 3 | 1.09 |
| C00581 | Guanidoacetic acid | 3 | 1.07 |
| C17349 | Guanidine | 3 | 1.07 |
| C04144 | Tetrahydropteroyltri-L-glutamate | 3 | 1.03 |
| C04489 | 5-Methyltetrahydropteroyltri-L-glutamate | 3 | 1.03 |
| C00365 | dUMP | 3 | 0.64 |
| C00286 | dGTP | 3 | 0.61 |
| C03283 | L-2,4-diaminobutyric acid | 3 | 0.59 |
| C11143 | Dimethyl sulfoxide | 3 | 0.59 |
| C00193 | Benzaldehyde | 3 | 0.47 |
| C00794 | Sorbitol | 3 | 0.27 |
| C00385 | Xanthine | 3 | 0.23 |
| C01561 | Calcidiol | 2 | 0.5 |
| C07130 | Theophylline | 2 | 0.1 |
| C02067 | Pseudouridine | 2 | 0.05 |
| C00092 | Glucose 6-phosphate | 2 | 0 |
| C00146 | Phenol | 2 | 0 |
