# Supplementary material for: Serum metabolomics identifies gut-derived uremic toxins and bile acid dysregulation associated with chronic kidney disease severity
Source: Sci Rep. 2026 Apr 14;16:12375. doi: 10.1038/s41598-026-44271-4 (PMC13083900; doi:10.1038/s41598-026-44271-4)
Supplement: Supplementary file 8 — Supplementary Material 8 [file 41598_2026_44271_MOESM8_ESM.docx]

**Table S8.** The significantly enriched functional pathways among metabolites within the interaction network

| **Pathway** | **Total** | **Expected** | **Hits** | **p. val** | **Topology** | **PVal.Z** | **Topo.Z** |
| --- | --- | --- | --- | --- | --- | --- | --- |
| Lipoic acid metabolism | 48 | 4.04 | 21 | 1.95E-11 | 0.489 | 3.52 | 0.0559 |
| Butanoate metabolism | 33 | 2.78 | 16 | 9.21E-10 | 1.5 | 2.87 | 1.91 |
| Glyoxylate and dicarboxylate metabolism | 39 | 3.28 | 14 | 1.13E-06 | 1.34 | 1.68 | 1.62 |
| Fatty acid biosynthesis | 36 | 3.03 | 13 | 2.61E-06 | 0.971 | 1.54 | 0.939 |
| Propanoate metabolism | 28 | 2.36 | 11 | 6.28E-06 | 1.22 | 1.39 | 1.4 |
| Carbon metabolism | 20 | 1.68 | 9 | 1.24E-05 | 1.16 | 1.27 | 1.28 |
| Inositol phosphate metabolism | 33 | 2.78 | 11 | 3.91E-05 | 0.812 | 1.08 | 0.648 |
| Starch and sucrose metabolism | 14 | 1.18 | 7 | 5.37E-05 | 1.23 | 1.03 | 1.41 |
| Glycosaminoglycan biosynthesis - chondroitin sulfate or dermatan sulfate | 15 | 1.26 | 7 | 9.36E-05 | 0.929 | 0.935 | 0.861 |
| Amino sugar and nucleotide sugar metabolism | 70 | 5.89 | 16 | 0.000127 | 0.913 | 0.884 | 0.832 |
| Glycerolipid metabolism | 21 | 1.77 | 8 | 0.000164 | 0.9 | 0.84 | 0.809 |
| Nitrogen metabolism | 8 | 0.674 | 5 | 0.00018 | 0.714 | 0.825 | 0.468 |
| Other types of O-glycan biosynthesis | 22 | 1.85 | 8 | 0.000239 | 0.81 | 0.777 | 0.643 |
| Linoleic acid metabolism | 28 | 2.36 | 9 | 0.000283 | 1.07 | 0.748 | 1.13 |
| Mucin type O-glycan biosynthesis | 23 | 1.94 | 8 | 0.000341 | 1 | 0.717 | 0.992 |
| Other glycan degradation | 28 | 2.36 | 8 | 0.0015 | 0.481 | 0.468 | 0.0415 |
| Steroid hormone biosynthesis | 4 | 0.337 | 3 | 0.0022 | 2 | 0.404 | 2.82 |
| Steroid biosynthesis | 8 | 0.674 | 4 | 0.00258 | 1.29 | 0.377 | 1.52 |
| Metabolic pathways | 26 | 2.19 | 7 | 0.00433 | 0.48 | 0.29 | 0.0388 |
| Glycosphingolipid biosynthesis - globo and isoglobo series | 20 | 1.68 | 6 | 0.0046 | 0.526 | 0.28 | 0.124 |
| Pantothenate and CoA biosynthesis | 6 | 0.505 | 3 | 0.00967 | 0.6 | 0.155 | 0.259 |
| Oxidative phosphorylation | 40 | 3.37 | 8 | 0.0156 | 0.41 | 0.0745 | -0.0891 |
| Fatty acid degradation | 42 | 3.54 | 8 | 0.0208 | 0.61 | 0.0267 | 0.277 |
| Glycosylphosphatidylinositol (GPI)-anchor biosynthesis | 9 | 0.758 | 3 | 0.0336 | 1.5 | -0.0543 | 1.91 |
| Fatty acid elongation | 16 | 1.35 | 4 | 0.0394 | 0.6 | -0.0809 | 0.259 |

Pathway: the name of KEGG metabolic pathway [20], total: total number of metabolites annotated to this pathway, expected: the expected number of hits under the null hypothesis of random metabolite selection, hits: the actual number of queries metabolites that map to this pathway, p. val: raw enrichment p-value, Topology: the pathway impact score calculated from degree of betweenness of the matched metabolites within the pathway, PVal.Z: Z-score of the enrichment p-value, Topo.Z: Z-score of topology impact scores across tested pathways.
