# Supplementary material for: Serum metabolomics identifies gut-derived uremic toxins and bile acid dysregulation associated with chronic kidney disease severity
Source: Sci Rep. 2026 Apr 14;16:12375. doi: 10.1038/s41598-026-44271-4 (PMC13083900; doi:10.1038/s41598-026-44271-4)
Supplement: Supplementary file 9 — Supplementary Material 9 [file 41598_2026_44271_MOESM9_ESM.docx]

| **Table S9:** Correlation analysis between differential metabolites and eGFR | | | |
| --- | --- | --- | --- |
| **a. Correlation analysis of differential metabolites with eGFR in eCKD and ESKD** | | | |
|  | **Correlation coefficient** | **p-value** | **FDR** |
| p-Hydroxyphenyllactic Acid | -0.8471 | 8.89E-15 | 1.69E-12 |
| Glutamyl-valine | -0.82355 | 2.09E-13 | 1.55E-11 |
| Xanthurenic acid | -0.82225 | 2.45E-13 | 1.55E-11 |
| Homogentisate | -0.81269 | 7.69E-13 | 3.65E-11 |
| Aspartyl-phenylalanine | -0.80985 | 1.07E-12 | 4.05E-11 |
| Acetyl carnitine | 0.79789 | 3.99E-12 | 1.26E-10 |
| 1-aminobutyrate | 0.79188 | 7.49E-12 | 1.94E-10 |
| Biliverdin | 0.79044 | 8.68E-12 | 1.94E-10 |
| Urea | -0.78991 | 9.17E-12 | 1.94E-10 |
| Pipecolate | 0.78717 | 1.21E-11 | 2.30E-10 |
| Pseudouridine | -0.78433 | 1.61E-11 | 2.77E-10 |
| Taurine | -0.78255 | 1.91E-11 | 3.03E-10 |
| Adipate | -0.77991 | 2.48E-11 | 3.62E-10 |
| Cortisone | 0.77909 | 2.68E-11 | 3.63E-10 |
| Prolyl-leucine | 0.77799 | 2.98E-11 | 3.77E-10 |
| 5-hydroxyindoleacetic acid | -0.77592 | 3.63E-11 | 4.26E-10 |
| Methylguanidine | -0.77539 | 3.81E-11 | 4.26E-10 |
| Valine | 0.76914 | 6.82E-11 | 7.04E-10 |
| Deoxycholate | -0.7678 | 7.71E-11 | 7.04E-10 |
| Phenylalanine | 0.76756 | 7.88E-11 | 7.04E-10 |
| Cytosine | -0.76722 | 8.13E-11 | 7.04E-10 |
| Trimethylamine N-Oxide | -0.7665 | 8.68E-11 | 7.04E-10 |
| Glycyl-proline | -0.76587 | 9.18E-11 | 7.04E-10 |
| Cortisol | 0.76578 | 9.26E-11 | 7.04E-10 |
| Taurocholate | -0.76578 | 9.26E-11 | 7.04E-10 |
| paraxanthine | 0.76525 | 9.71E-11 | 7.10E-10 |
| Suberate | -0.76313 | 1.17E-10 | 8.26E-10 |
| Lysyl-proline | 0.76227 | 1.27E-10 | 8.60E-10 |
| Threonine | -0.76136 | 1.37E-10 | 9.00E-10 |
| 3-Phenylpropionate | -0.75991 | 1.56E-10 | 9.87E-10 |
| Lithocholate | -0.75842 | 1.78E-10 | 1.09E-09 |
| L-Glutamine | -0.75592 | 2.20E-10 | 1.31E-09 |
| Thymol sulfate | -0.7553 | 2.32E-10 | 1.33E-09 |
| Cystine | -0.75506 | 2.37E-10 | 1.33E-09 |
| Adenine | -0.75472 | 2.44E-10 | 1.33E-09 |
| Kynurenic acid | -0.75318 | 2.78E-10 | 1.45E-09 |
| Methioninesulfoxide | -0.75299 | 2.83E-10 | 1.45E-09 |
| Dimethylarginine | 0.7527 | 2.90E-10 | 1.45E-09 |
| Indoxyl sulphate | -0.749 | 3.95E-10 | 1.93E-09 |
| Glycochenodeoxycholate | -0.74746 | 4.49E-10 | 2.13E-09 |
| Serine | 0.74473 | 5.62E-10 | 2.61E-09 |
| 3-hydroxyanthranilate | -0.74136 | 7.38E-10 | 3.34E-09 |
| N-formyl methionine | -0.74078 | 7.73E-10 | 3.35E-09 |
| Ornithine | 0.74069 | 7.79E-10 | 3.35E-09 |
| Lactate | 0.74045 | 7.94E-10 | 3.35E-09 |
| Asparagine | -0.73929 | 8.71E-10 | 3.60E-09 |
| Phenol sulfate | -0.73867 | 9.15E-10 | 3.70E-09 |
| 16-hydroxypalmitate | 0.73737 | 1.01E-09 | 3.99E-09 |
| Norleucine | 0.73718 | 1.03E-09 | 3.99E-09 |
| Creatinine | -0.73295 | 1.43E-09 | 5.44E-09 |
| 2-phenylglycine | -0.73107 | 1.65E-09 | 6.16E-09 |
| Succinate | 0.72511 | 2.60E-09 | 9.49E-09 |
| Hydroquinone | -0.72454 | 2.71E-09 | 9.72E-09 |
| N-hydroxy-valine | 0.72348 | 2.93E-09 | 1.03E-08 |
| Calcitriol | 0.72305 | 3.03E-09 | 1.05E-08 |
| octadecanedioate (C18) | 0.71978 | 3.85E-09 | 1.31E-08 |
| Betaine | 0.71401 | 5.84E-09 | 1.95E-08 |
| Myo-inositol | -0.71142 | 7.01E-09 | 2.27E-08 |
| Uric acid | 0.71122 | 7.11E-09 | 2.27E-08 |
| 3-hydroxydecanoate | 0.71089 | 7.28E-09 | 2.27E-08 |
| Indolepropionate | 0.71089 | 7.28E-09 | 2.27E-08 |
| Homoarginine | 0.71012 | 7.68E-09 | 2.35E-08 |
| Glycyl-valine | -0.70661 | 9.81E-09 | 2.93E-08 |
| Methylmalonate | -0.70632 | 1.00E-08 | 2.93E-08 |
| Glycocholate | -0.70627 | 1.00E-08 | 2.93E-08 |
| Glycerophosphoryl choline | -0.70339 | 1.22E-08 | 3.52E-08 |
| Anthranilate | -0.70305 | 1.25E-08 | 3.54E-08 |
| 5-dodecenoate (12:1n7) | 0.70286 | 1.27E-08 | 3.54E-08 |
| Inosine | -0.70103 | 1.43E-08 | 3.95E-08 |
| Prolyl-lysine | 0.69546 | 2.08E-08 | 5.65E-08 |
| Homocysteine | -0.69522 | 2.11E-08 | 5.66E-08 |
| N-Acetylneuraminic Acid | -0.69147 | 2.70E-08 | 7.13E-08 |
| Pyruvate | -0.68017 | 5.54E-08 | 1.44E-07 |
| Lysophosphatidylcholine (18:1) | 0.6796 | 5.74E-08 | 1.47E-07 |
| Stearic acid | -0.67551 | 7.38E-08 | 1.87E-07 |
| Homovanillate | -0.6733 | 8.44E-08 | 2.11E-07 |
| Oleic acid | -0.67046 | 1.00E-07 | 2.47E-07 |
| Tyrosine | 0.65888 | 1.97E-07 | 4.80E-07 |
| N6-Methyllysine | -0.64912 | 3.41E-07 | 8.21E-07 |
| Theophylline | 0.6472 | 3.80E-07 | 9.01E-07 |
| Sarcosine | 0.6423 | 4.96E-07 | 1.16E-06 |
| Phenylacetylglutamine | -0.6398 | 5.67E-07 | 1.31E-06 |
| Thymidine | -0.63562 | 7.07E-07 | 1.62E-06 |
| Prolyl-tyrosine | 0.6347 | 7.42E-07 | 1.68E-06 |
| Proline | 0.62206 | 1.42E-06 | 3.17E-06 |
| Dihydrocapsaicin | 0.6049 | 3.27E-06 | 7.23E-06 |
| Porphobilinogen | -0.56708 | 1.76E-05 | 3.84E-05 |
| Linoleic acid | -0.55516 | 2.86E-05 | 6.18E-05 |
| Arachidonate | -0.55328 | 3.08E-05 | 6.58E-05 |
| γ-glutamyl-methionine | 0.5442 | 4.41E-05 | 9.30E-05 |
| N-acetylglutamine | -0.51387 | 0.00013516 | 0.00028007 |
| heptanoate (7:0) | -0.51377 | 0.00013561 | 0.00028007 |
| Histidine | 0.47806 | 0.00044614 | 0.0009114 |
| Pantothenic Acid | 0.47748 | 0.00045432 | 0.0009114 |
| Arginine | -0.47739 | 0.0004557 | 0.0009114 |
| 2-hydroxyisovalerate | 0.46686 | 0.00063147 | 0.0012496 |
| Hydroxyisocaproate | -0.46652 | 0.00063797 | 0.0012496 |
| phenylacetate | 0.43158 | 0.001752 | 0.0033967 |
| Diethanolamine | 0.41841 | 0.002496 | 0.0047903 |
| Glutathione | 0.41702 | 0.0025892 | 0.0049194 |
| Theobromine | 0.415 | 0.0027295 | 0.0051348 |
| Serotonin | 0.40971 | 0.0031296 | 0.0058296 |
| 4-hydroxyphenyl acetic acid | -0.4051 | 0.0035201 | 0.0064934 |
| 3-methylhistidine | -0.40187 | 0.0038175 | 0.0069744 |
| Orotate | -0.39173 | 0.0049035 | 0.008873 |
| Glucosamine | 0.38529 | 0.0057259 | 0.010263 |
| p-cresyl sulfate | 0.38395 | 0.0059122 | 0.010498 |
| Laurate (12:0) | -0.38236 | 0.0061385 | 0.010799 |
| Butyrylcarnitine | 0.37395 | 0.0074694 | 0.012902 |
| Bilirubin | 0.36664 | 0.008822 | 0.015101 |
| 4-vinylphenol sulfate | -0.35953 | 0.010338 | 0.017537 |
| Glycerol 3-Phosphate | -0.3428 | 0.014809 | 0.0249 |
| N-acetylornithine | 0.33886 | 0.016075 | 0.026792 |
| 2-hydoxystearate | 0.33665 | 0.016825 | 0.027798 |
| Decanoic acid | 0.33569 | 0.01716 | 0.028108 |
| Methionine | 0.32564 | 0.021016 | 0.034128 |
| Niacinamide | 0.32295 | 0.022165 | 0.03569 |
| Tryptophan | -0.31507 | 0.025839 | 0.041256 |
| Xanthosine | -0.31166 | 0.027581 | 0.04367 |
| Lysyl-glutamate | 0.30546 | 0.030997 | 0.048673 |
| γ-glutamyl-tyrosine | 0.30348 | 0.032153 | 0.050075 |
| Acetone | -0.30041 | 0.03403 | 0.052512 |
| Ascorbate | 0.30002 | 0.034271 | 0.052512 |
| γ-glutamyl-phenylalanine | 0.29613 | 0.03679 | 0.055921 |
| Undecanoic acid | -0.29464 | 0.037794 | 0.05699 |
| Feruloylputrescine | 0.28421 | 0.045468 | 0.068022 |
| 3-Hydroxyisovalerate | 0.28147 | 0.047682 | 0.070777 |
| Indoleacetate | -0.27623 | 0.052159 | 0.076732 |
| 17-methyloctadecanoic acid | -0.27585 | 0.052501 | 0.076732 |
| 4-pyridoxate | -0.26859 | 0.059296 | 0.085554 |
| Malonate | -0.26845 | 0.059437 | 0.085554 |
| Docosahexaenoic acid | 0.26676 | 0.061112 | 0.087303 |
| 4-Methyl-2-oxopentanoate | -0.26484 | 0.063073 | 0.089431 |
| 3-methyl-2-oxovalerate | -0.26075 | 0.067405 | 0.094867 |
| Dimethylglycine | -0.25475 | 0.074204 | 0.10367 |
| Argininosuccinic Acid | -0.24956 | 0.080505 | 0.11165 |
| 3-methoxytyrosine | -0.24605 | 0.084998 | 0.11703 |
| Lactose | -0.23922 | 0.094296 | 0.12889 |
| N-acetyl-alanine | -0.23788 | 0.09622 | 0.13058 |
| Spermine | -0.23172 | 0.1054 | 0.14203 |
| Xanthine | -0.23067 | 0.10705 | 0.14323 |
| Glucose | -0.2285 | 0.11047 | 0.14678 |
| Glutamate | -0.22586 | 0.11477 | 0.15143 |
| Cysteinyl-glycine | -0.22495 | 0.11628 | 0.15237 |
| Malic Acid | -0.22163 | 0.12191 | 0.15864 |
| Beta-Hydroxybutyrate | -0.22019 | 0.12441 | 0.16081 |
| Allantoin | -0.21966 | 0.12534 | 0.16091 |
| Palmitic acid | -0.2137 | 0.13619 | 0.17366 |
| Cinnamic acid | -0.21 | 0.14327 | 0.18147 |
| hydrochlorothiazide | -0.20402 | 0.15528 | 0.19538 |
| γ-glutamyl-leucine | -0.20308 | 0.15723 | 0.19654 |
| Citrulline | -0.19923 | 0.16541 | 0.20541 |
| Palmitoylcarnitine | -0.19846 | 0.16708 | 0.20614 |
| Sphingomyelin | -0.19505 | 0.17465 | 0.21409 |
| Oxalate | -0.19101 | 0.18392 | 0.22401 |
| Myristoleate | -0.18621 | 0.19541 | 0.23499 |
| 1-methylxanthine | -0.18621 | 0.19541 | 0.23499 |
| Hypotaurine | -0.18049 | 0.20974 | 0.25054 |
| Citrate | -0.18 | 0.21098 | 0.25054 |
| Gluconic acid | -0.17549 | 0.22285 | 0.26299 |
| Alpha-Ketoglutarate | 0.17246 | 0.23106 | 0.271 |
| Hippurate | -0.16861 | 0.24179 | 0.28184 |
| glycolithocholate sulphate | -0.16486 | 0.25257 | 0.29261 |
| 2-Keto Tridecanoic Acid | 0.15616 | 0.27882 | 0.32106 |
| Hexanoylcarnitine | -0.15371 | 0.28653 | 0.32795 |
| Eicosenoate | -0.15165 | 0.29313 | 0.3335 |
| Oleic acid-2-6-diisopropylanilide | -0.14261 | 0.32317 | 0.36549 |
| Hydroxyproline | -0.13636 | 0.34504 | 0.38791 |
| Catechol sulfate | 0.13429 | 0.35247 | 0.39394 |
| Palmitoleate (16:1n17) | 0.1317 | 0.36194 | 0.40215 |
| Ursodeoxycholate | -0.12997 | 0.36833 | 0.40688 |
| Carnosine | 0.099688 | 0.49096 | 0.5392 |
| Leucine | 0.096515 | 0.50492 | 0.55135 |
| Vitamin B2 | 0.093728 | 0.51736 | 0.56171 |
| Adrenate | -0.084066 | 0.56162 | 0.6063 |
| Nonadecanal | -0.081134 | 0.5754 | 0.61766 |
| Glycyl-tyrosine | -0.078923 | 0.58589 | 0.62538 |
| Cholesteryl sulphate | -0.076664 | 0.59669 | 0.63336 |
| Dihydrobiopterin | -0.069358 | 0.63222 | 0.66734 |
| 10-heptadecenoate (17:1n7) | -0.062293 | 0.66738 | 0.69671 |
| Aspartate | -0.061428 | 0.67173 | 0.69743 |
| Isoleucine | 0.049411 | 0.73328 | 0.75719 |
| Ceramide | -0.046143 | 0.75034 | 0.77062 |
| Phosphocholine | -0.041817 | 0.77309 | 0.78971 |
| Nonadecanoiate | 0.029993 | 0.83619 | 0.84961 |
| 5-oxoproline | -0.021389 | 0.88279 | 0.89218 |
| Myristate | -0.019947 | 0.89064 | 0.89535 |
| Choline | -0.00081711 | 0.99551 | 0.99551 |
| **b. Correlation analysis of differential metabolites with eGFR in ESKD** | | | |
|  | **Correlation coefficient** | **p-value** | **FDR** |
| p-Hydroxyphenyllactic Acid | -0.70028 | 9.72E-07 | 0.00018472 |
| Glycyl-tyrosine | 0.54732 | 4.6307E-06 | 0.00032793 |
| N-acetylornithine | -0.54153 | 5.1779E-06 | 0.00032793 |
| Cytosine | -0.47432 | 0.00016596 | 0.0078833 |
| Indoxyl sulfate | -0.44883 | 0.00024417 | 0.0080887 |
| Succinate | 0.42642 | 0.00033532 | 0.0080887 |
| Phenol sulfate | -0.41561 | 0.0018812 | 0.010887 |
| Choline | -0.41097 | 0.005266 | 0.020887 |
| Stearic acid | 0.39398 | 0.01333 | 0.030887 |
| Theophylline | -0.3766 | 0.063515 | 0.80887 |
| Serotonin | 0.36617 | 0.071826 | 0.80887 |
| Myo-inositol | -0.36501 | 0.072798 | 0.80887 |
| Glucosamine | 0.36115 | 0.076112 | 0.80887 |
| Hydroxyproline | -0.35728 | 0.07954 | 0.80887 |
| 4-Methyl-2-oxopentanoate | 0.35188 | 0.084533 | 0.80887 |
| Gluconic acid | 0.3484 | 0.087866 | 0.80887 |
| Aspartate | 0.34686 | 0.089378 | 0.80887 |
| Dimethylarginine | 0.34299 | 0.093245 | 0.80887 |
| Asparagine | -0.34029 | 0.096025 | 0.80887 |
| Phenylacetylglutamine | -0.33836 | 0.098049 | 0.80887 |
| γ-glutamyl-leucine | -0.33681 | 0.099691 | 0.80887 |
| Methylmalonate | 0.33527 | 0.10135 | 0.80887 |
| 3-methoxytyrosine | 0.33295 | 0.10388 | 0.80887 |
| Hexanoylcarnitine | 0.33063 | 0.10646 | 0.80887 |
| 3-methylhistidine | -0.32909 | 0.10821 | 0.80887 |
| Nonadecanoiate | 0.32214 | 0.11632 | 0.80887 |
| 5-oxoproline | -0.32059 | 0.11818 | 0.80887 |
| N-Acetylneuraminic Acid | 0.31055 | 0.13082 | 0.80887 |
| Isoleucine | 0.30862 | 0.13336 | 0.80887 |
| Xanthine | -0.29973 | 0.14549 | 0.80887 |
| Norleucine | 0.2978 | 0.14823 | 0.80887 |
| 2-phenylglycine | -0.2951 | 0.15212 | 0.80887 |
| Adrenate | -0.29432 | 0.15325 | 0.80887 |
| Butyrylcarnitine | 0.28776 | 0.16306 | 0.80887 |
| Glutamyl-valine | -0.28737 | 0.16365 | 0.80887 |
| 2-hydroxyisovalerate | 0.28583 | 0.16603 | 0.80887 |
| Myristate | 0.28428 | 0.16843 | 0.80887 |
| phenylacetate | 0.28196 | 0.17208 | 0.80887 |
| Hypotaurine | 0.28119 | 0.17331 | 0.80887 |
| Indolepropionate | -0.28081 | 0.17393 | 0.80887 |
| Arachidonate | -0.28042 | 0.17455 | 0.80887 |
| 2-hydoxystearate | -0.26536 | 0.19985 | 0.83501 |
| Arginine | 0.26497 | 0.20053 | 0.83501 |
| Cortisol | 0.26265 | 0.20464 | 0.83501 |
| Ursodeoxycholate | 0.25647 | 0.21589 | 0.83501 |
| 1-methylxanthine | -0.25609 | 0.2166 | 0.83501 |
| Bilirubin | 0.25454 | 0.21949 | 0.83501 |
| Suberate | -0.25261 | 0.22312 | 0.83501 |
| Palmitic acid | 0.25184 | 0.22459 | 0.83501 |
| 4-pyridoxate | -0.24913 | 0.22977 | 0.83501 |
| Glycerophosphoryl choline | 0.24373 | 0.24038 | 0.83501 |
| 4-hydroxyphenyl acetic acid | -0.24334 | 0.24115 | 0.83501 |
| 3-Phenylpropionate | -0.24295 | 0.24192 | 0.83501 |
| Adipate | -0.24179 | 0.24424 | 0.83501 |
| Proline | 0.24064 | 0.24658 | 0.83501 |
| Argininosuccinic Acid | -0.2387 | 0.2505 | 0.83501 |
| Histidine | -0.2387 | 0.2505 | 0.83501 |
| Allantoin | 0.22634 | 0.2766 | 0.89892 |
| 3-methyl-2-oxovalerate | -0.22519 | 0.27914 | 0.89892 |
| Hippurate | 0.21398 | 0.30437 | 0.91366 |
| Citrate | -0.21205 | 0.30886 | 0.91366 |
| Phenylalanine | 0.21167 | 0.30976 | 0.91366 |
| Dimethylglycine | 0.21128 | 0.31067 | 0.91366 |
| Cinnamic acid | -0.20974 | 0.3143 | 0.91366 |
| Pyruvate | 0.20896 | 0.31613 | 0.91366 |
| Dihydrobiopterin | -0.20665 | 0.32165 | 0.91366 |
| Carnosine | -0.2024 | 0.33191 | 0.91366 |
| Sphingomyelin | -0.20047 | 0.33665 | 0.91366 |
| Theobromine | -0.20008 | 0.3376 | 0.91366 |
| Methylguanidine | -0.19853 | 0.34142 | 0.91366 |
| Malonate | 0.19274 | 0.35598 | 0.92907 |
| 10-heptadecenoate (17:1n7) | 0.19235 | 0.35696 | 0.92907 |
| γ-glutamyl-phenylalanine | -0.18733 | 0.36989 | 0.93864 |
| Glycerol 3-Phosphate | -0.18424 | 0.37798 | 0.93864 |
| Anthranilate | 0.18154 | 0.38514 | 0.93864 |
| 1-aminobutyrate | 0.18115 | 0.38616 | 0.93864 |
| Valine | 0.17575 | 0.40074 | 0.93864 |
| Acetone | 0.17497 | 0.40284 | 0.93864 |
| 17-methyloctadecanoic acid | 0.17459 | 0.4039 | 0.93864 |
| Glycyl-proline | -0.17266 | 0.4092 | 0.93864 |
| Ornithine | -0.16995 | 0.41668 | 0.93864 |
| Beta-Hydroxybutyrate | 0.16995 | 0.41668 | 0.93864 |
| Citrulline | 0.16957 | 0.41776 | 0.93864 |
| Feruloylputrescine | 0.16879 | 0.41992 | 0.93864 |
| Oxalate | 0.16532 | 0.42969 | 0.94932 |
| Hydroquinone | 0.15875 | 0.44849 | 0.95209 |
| Prolyl-lysine | -0.1572 | 0.45297 | 0.95209 |
| Malic Acid | -0.15682 | 0.4541 | 0.95209 |
| Kynurenic acid | -0.15411 | 0.46201 | 0.95209 |
| Homogentisate | -0.15411 | 0.46201 | 0.95209 |
| 3-hydroxydecanoate | 0.1518 | 0.46885 | 0.95209 |
| Pantothenic Acid | -0.15102 | 0.47115 | 0.95209 |
| heptanoate (7:0) | 0.14639 | 0.48502 | 0.95209 |
| Indoleacetate | 0.146 | 0.48618 | 0.95209 |
| Spermine | 0.14407 | 0.49203 | 0.95209 |
| Leucine | 0.14407 | 0.49203 | 0.95209 |
| p-cresyl sulfate | -0.14175 | 0.4991 | 0.95209 |
| paraxanthine | 0.14175 | 0.4991 | 0.95209 |
| Pseudouridine | -0.13944 | 0.50621 | 0.95209 |
| Homovanillate | 0.13789 | 0.51098 | 0.95209 |
| Sarcosine | -0.13673 | 0.51457 | 0.95209 |
| Thymol sulfate | -0.1321 | 0.52906 | 0.95209 |
| 3-Hydroxyisovalerate | 0.12824 | 0.54128 | 0.95209 |
| Methionine | -0.12437 | 0.55362 | 0.95209 |
| Betaine | -0.12051 | 0.5661 | 0.95209 |
| 16-hydroxypalmitate | 0.11819 | 0.57365 | 0.95209 |
| Tryptophan | -0.11588 | 0.58124 | 0.95209 |
| N-formyl methionine | 0.1151 | 0.58377 | 0.95209 |
| Aspartyl-phenylalanine | -0.1151 | 0.58377 | 0.95209 |
| Hydroxyisocaproate | -0.1124 | 0.5927 | 0.95209 |
| 3-hydroxyanthranilate | 0.11008 | 0.6004 | 0.95209 |
| Glucose | 0.1097 | 0.60169 | 0.95209 |
| Prolyl-tyrosine | -0.1097 | 0.60169 | 0.95209 |
| Glutathione | 0.10776 | 0.60814 | 0.95209 |
| octadecanedioate (C18) | 0.10699 | 0.61073 | 0.95209 |
| Cholesteryl sulphate | -0.10583 | 0.61462 | 0.95209 |
| N-hydroxy-valine | 0.10236 | 0.62635 | 0.95209 |
| γ-glutamyl-tyrosine | -0.10197 | 0.62766 | 0.95209 |
| Decanoic acid | 0.10158 | 0.62897 | 0.95209 |
| N6-Methyllysine | 0.10158 | 0.62897 | 0.95209 |
| Undecanoic acid | -0.1012 | 0.63029 | 0.95209 |
| N-acetyl-alanine | -0.10043 | 0.63291 | 0.95209 |
| Linoleic acid | -0.098108 | 0.64082 | 0.95209 |
| Biliverdin | 0.096177 | 0.64744 | 0.95209 |
| Calcitriol | 0.095018 | 0.65142 | 0.95209 |
| Glutamate | -0.095018 | 0.65142 | 0.95209 |
| Cortisone | 0.094246 | 0.65408 | 0.95209 |
| Porphobilinogen | 0.093473 | 0.65674 | 0.95209 |
| Oleic acid | 0.090383 | 0.66744 | 0.95209 |
| 4-vinylphenol sulfate | -0.089611 | 0.67013 | 0.95209 |
| Xanthurenic acid | -0.089611 | 0.67013 | 0.95209 |
| Phosphocholine | 0.089224 | 0.67147 | 0.95209 |
| Alpha-Ketoglutarate | 0.086134 | 0.68226 | 0.95301 |
| Inosine | 0.085748 | 0.68361 | 0.95301 |
| Xanthosine | -0.084589 | 0.68767 | 0.95301 |
| Taurine | -0.082272 | 0.69582 | 0.95301 |
| Nonadecanal | -0.079954 | 0.70401 | 0.95301 |
| Pipecolate | 0.078409 | 0.70948 | 0.95301 |
| N-acetylglutamine | -0.076864 | 0.71497 | 0.95301 |
| Urea | -0.076864 | 0.71497 | 0.95301 |
| Palmitoleate (16:1n17) | 0.076092 | 0.71772 | 0.95301 |
| Threonine | 0.072229 | 0.73152 | 0.95301 |
| Lithocholate | 0.071843 | 0.7329 | 0.95301 |
| hydrochlorothiazide | -0.070891 | 0.73632 | 0.95301 |
| Dihydrocapsaicin | 0.068753 | 0.74401 | 0.95301 |
| 5-dodecenoate (12:1n7) | 0.067981 | 0.74679 | 0.95301 |
| Catechol sulfate | -0.067208 | 0.74958 | 0.95301 |
| L-Glutamine | -0.063732 | 0.76215 | 0.95301 |
| 2-Keto Tridecanoic Acid | -0.0618 | 0.76917 | 0.95301 |
| Orotate | 0.060642 | 0.77338 | 0.95301 |
| Uric acid | -0.059869 | 0.7762 | 0.95301 |
| Glycocholate | 0.059483 | 0.77761 | 0.95301 |
| γ-glutamyl-methionine | -0.057938 | 0.78325 | 0.95301 |
| Methioninesulfoxide | -0.057552 | 0.78466 | 0.95301 |
| Ceramide | -0.056779 | 0.78748 | 0.95301 |
| Palmitoylcarnitine | -0.053303 | 0.80023 | 0.95924 |
| Lactose | 0.047895 | 0.82015 | 0.95924 |
| Serine | 0.047123 | 0.82301 | 0.95924 |
| Taurocholate | 0.046737 | 0.82444 | 0.95924 |
| Thymidine | -0.045964 | 0.8273 | 0.95924 |
| 5-hydroxyindoleacetic acid | -0.045578 | 0.82873 | 0.95924 |
| Myristoleate | 0.045192 | 0.83016 | 0.95924 |
| Lysyl-glutamate | -0.044419 | 0.83302 | 0.95924 |
| Creatinine | -0.042488 | 0.84019 | 0.96166 |
| Glycochenodeoxycholate | -0.039784 | 0.85024 | 0.96734 |
| Niacinamide | 0.036308 | 0.8632 | 0.97208 |
| Lysophosphatidylcholine (18:1) | -0.035922 | 0.86464 | 0.97208 |
| Cystine | 0.034377 | 0.87042 | 0.97282 |
| Tyrosine | -0.030128 | 0.88632 | 0.97404 |
| Laurate (12:0) | -0.029355 | 0.88922 | 0.97404 |
| Prolyl-leucine | 0.028969 | 0.89067 | 0.97404 |
| Trimethylamine N-Oxide | 0.026651 | 0.89937 | 0.97404 |
| Eicosenoate | -0.026265 | 0.90082 | 0.97404 |
| Acetyl carnitine | -0.025879 | 0.90227 | 0.97404 |
| Glycyl-valine | 0.022016 | 0.91681 | 0.97861 |
| Oleic acid-2-6-diisopropylanilide | -0.020471 | 0.92263 | 0.97932 |
| glycolithocholate sulphate | 0.016223 | 0.93865 | 0.9908 |
| Homocysteine | -0.010043 | 0.962 | 0.99708 |
| Adenine | -0.0092701 | 0.96492 | 0.99708 |
| Lysyl-proline | 0.0081113 | 0.9693 | 0.99708 |
| Cysteinyl-glycine | 0.0073388 | 0.97223 | 0.99708 |
| Docosahexaenoic acid | 0.0065663 | 0.97515 | 0.99708 |
| Ascorbate | 0.0050213 | 0.981 | 0.99708 |
| Deoxycholate | -0.0038625 | 0.98538 | 0.99708 |
| Diethanolamine | -0.0023175 | 0.99123 | 0.99708 |
| Homoarginine | 0.001545 | 0.99415 | 0.99708 |
| Lactate | -0.00077251 | 0.99708 | 0.99708 |
| **c. Correlation analysis of differential metabolites with eGFR in eCKD** | | | |
|  | **Correlation coefficient** | **p-value** | **FDR** |
| Carnosine | -0.56465 | 0.00003276 | 0.0035311 |
| Leucine | 0.5523 | 0.000042003 | 0.0035311 |
| Lysophosphatidylcholine (18:1) | -0.53763 | 0.000055754 | 0.0035311 |
| p-cresyl sulfate | 0.58023 | 0.000091643 | 0.0043531 |
| Xanthurenic acid | -0.5802 | 0.00014588 | 0.0053221 |
| Cholesteryl sulphate | 0.57241 | 0.00017101 | 0.0053221 |
| 2-phenylglycine | 0.55504 | 0.00022281 | 0.0053221 |
| 5-dodecenoate (12:1n7) | -0.55465 | 0.00022409 | 0.003221 |
| Asparagine | 0.5342 | 0.0030104 | 0.02508 |
| 3-hydroxydecanoate | -0.53381 | 0.0030267 | 0.027508 |
| 3-methoxytyrosine | -0.51258 | 0.00404 | 0.028293 |
| Norleucine | -0.50872 | 0.0042501 | 0.029293 |
| Acetyl carnitine | 0.50216 | 0.0046269 | 0.037624 |
| Cysteinyl-glycine | 0.48325 | 0.0048612 | 0.038215 |
| Theophylline | -0.37515 | 0.064624 | 0.7215 |
| Isoleucine | 0.37438 | 0.06522 | 0.7215 |
| Alpha-Ketoglutarate | -0.36974 | 0.068888 | 0.7215 |
| Myo-inositol | -0.36859 | 0.069829 | 0.7215 |
| Aspartyl-phenylalanine | -0.35739 | 0.079441 | 0.7215 |
| Diethanolamine | 0.34967 | 0.086633 | 0.7215 |
| 3-methyl-2-oxovalerate | 0.3435 | 0.092731 | 0.7215 |
| Homogentisate | -0.34118 | 0.095099 | 0.7215 |
| Dihydrocapsaicin | 0.3381 | 0.098326 | 0.7215 |
| Glycylvaline | 0.33462 | 0.10205 | 0.7215 |
| Homoarginine | -0.32999 | 0.10718 | 0.7215 |
| N-hydroxy-valine | -0.32343 | 0.11478 | 0.7215 |
| Calcitriol | -0.31957 | 0.11942 | 0.7215 |
| Inosine | 0.31571 | 0.12421 | 0.7215 |
| Butyrylcarnitine | -0.31417 | 0.12616 | 0.7215 |
| Hydroxyproline | 0.31224 | 0.12863 | 0.7215 |
| Histidine | 0.30259 | 0.14151 | 0.7215 |
| Glycocholate | 0.29989 | 0.14527 | 0.7215 |
| Glutathione | 0.29989 | 0.14527 | 0.7215 |
| Malonate | -0.2995 | 0.14582 | 0.7215 |
| Hexanoylcarnitine | 0.2995 | 0.14582 | 0.7215 |
| Homovanillate | -0.29873 | 0.14691 | 0.7215 |
| γ-glutamyl-leucine | 0.29796 | 0.14801 | 0.7215 |
| Glutamate | -0.2968 | 0.14966 | 0.7215 |
| Glutamyl-valine | -0.29525 | 0.1519 | 0.7215 |
| Homocysteine | 0.29101 | 0.15815 | 0.7329 |
| Citrulline | -0.28406 | 0.16878 | 0.75112 |
| 1-aminobutyrate | 0.28329 | 0.16999 | 0.75112 |
| Prolyl-tyrosine | -0.28059 | 0.17428 | 0.75255 |
| Hydroxyisocaproate | 0.27557 | 0.18243 | 0.75569 |
| Sphingomyelin | 0.27441 | 0.18435 | 0.75569 |
| 2-hydoxystearate | 0.27287 | 0.18693 | 0.75569 |
| Uric acid | -0.25975 | 0.20988 | 0.78549 |
| Pantothenic Acid | 0.25936 | 0.21058 | 0.78549 |
| Laurate (12:0) | -0.25589 | 0.21697 | 0.78549 |
| Nonadecanal | 0.25473 | 0.21913 | 0.78549 |
| Decanoic acid | 0.25473 | 0.21913 | 0.78549 |
| γ-glutamyl-methionine | -0.2528 | 0.22276 | 0.78549 |
| Phenol sulfate | 0.24431 | 0.23922 | 0.78549 |
| Hippurate | -0.24392 | 0.23998 | 0.78549 |
| Niacinamide | 0.24392 | 0.23998 | 0.78549 |
| Phosphocholine | 0.24084 | 0.24617 | 0.78549 |
| Feruloylputrescine | 0.23813 | 0.25167 | 0.78549 |
| 17-methyloctadecanoic acid | -0.2362 | 0.25565 | 0.78549 |
| Urea | -0.23543 | 0.25725 | 0.78549 |
| Aspartate | -0.23427 | 0.25967 | 0.78549 |
| Xanthine | 0.23312 | 0.2621 | 0.78549 |
| Catechol sulfate | -0.23157 | 0.26536 | 0.78549 |
| Oleic acid-2-6-diisopropylanilide | -0.22964 | 0.26948 | 0.78549 |
| Ceramide | 0.22771 | 0.27363 | 0.78549 |
| Thymidine | 0.22655 | 0.27615 | 0.78549 |
| Prolyl-lysine | -0.22617 | 0.27699 | 0.78549 |
| Indoxyl sulphate | 0.22115 | 0.28807 | 0.79799 |
| Biliverdin | 0.22038 | 0.2898 | 0.79799 |
| Pipecolate | 0.21189 | 0.30924 | 0.83938 |
| Palmitoleate (16:1n17) | -0.20378 | 0.32854 | 0.8569 |
| Anthranilate | 0.20378 | 0.32854 | 0.8569 |
| Dihydrobiopterin | 0.20301 | 0.33042 | 0.8569 |
| octadecanedioate (C18) | -0.20031 | 0.33703 | 0.8569 |
| Indoleacetate | -0.19838 | 0.3418 | 0.8569 |
| Undecanoic acid | 0.19799 | 0.34276 | 0.8569 |
| Stearic acid | -0.18989 | 0.36327 | 0.86852 |
| Sarcosine | 0.18835 | 0.36726 | 0.86852 |
| Creatinine | 0.18757 | 0.36926 | 0.86852 |
| Prolyl-leucine | 0.18757 | 0.36926 | 0.86852 |
| Acetone | -0.18256 | 0.38243 | 0.88612 |
| N-acetylornithine | 0.17792 | 0.39483 | 0.90382 |
| 3-Phenylpropionate | 0.17136 | 0.41277 | 0.90789 |
| Taurine | -0.17098 | 0.41383 | 0.90789 |
| N-Acetylneuraminic Acid | 0.16789 | 0.42244 | 0.90789 |
| Lactose | 0.16712 | 0.42461 | 0.90789 |
| 10-heptadecenoate (17:1n7) | 0.16673 | 0.4257 | 0.90789 |
| 3-methylhistidine | -0.16557 | 0.42896 | 0.90789 |
| Taurocholate | -0.16519 | 0.43005 | 0.90789 |
| 4-hydroxyphenyl acetic acid | -0.15824 | 0.44996 | 0.92037 |
| Dimethylarginine | -0.15631 | 0.45558 | 0.92037 |
| Threonine | -0.15515 | 0.45896 | 0.92037 |
| 5-hydroxyindoleacetic acid | -0.15438 | 0.46123 | 0.92037 |
| Allantoin | 0.15322 | 0.46464 | 0.92037 |
| Cystine | -0.15129 | 0.47035 | 0.92037 |
| Trimethylamine N-Oxide | -0.15091 | 0.47149 | 0.92037 |
| Methioninesulfoxide | -0.14936 | 0.47609 | 0.92037 |
| Serotonin | -0.14821 | 0.47956 | 0.92037 |
| Glucosamine | 0.14126 | 0.50061 | 0.95116 |
| Palmitic acid | -0.13547 | 0.5185 | 0.96453 |
| hydrochlorothiazide | -0.13508 | 0.5197 | 0.96453 |
| Cortisone | 0.13122 | 0.53181 | 0.96453 |
| Lithocholate | -0.13122 | 0.53181 | 0.96453 |
| Deoxycholate | -0.13084 | 0.53303 | 0.96453 |
| Pseudouridine | -0.12814 | 0.54159 | 0.9655 |
| Ursodeoxycholate | 0.12698 | 0.54528 | 0.9655 |
| Cinnamic acid | -0.12003 | 0.56766 | 0.9655 |
| 3-Hydroxyisovalerate | 0.11579 | 0.58153 | 0.9655 |
| Lysyl-glutamate | 0.1073 | 0.60971 | 0.9655 |
| phenylacetate | -0.10537 | 0.61619 | 0.9655 |
| Ascorbate | 0.10498 | 0.61749 | 0.9655 |
| Myristate | 0.10459 | 0.61879 | 0.9655 |
| Suberate | -0.10073 | 0.63186 | 0.9655 |
| Hypotaurine | -0.097646 | 0.6424 | 0.9655 |
| 4-Methyl-2-oxopentanoate | 0.096874 | 0.64504 | 0.9655 |
| Gluconic acid | 0.094559 | 0.653 | 0.9655 |
| Argininosuccinic Acid | -0.092629 | 0.65966 | 0.9655 |
| glycolithocholate sulphate | 0.091471 | 0.66367 | 0.9655 |
| γ-glutamyl-tyrosine | 0.090699 | 0.66635 | 0.9655 |
| Kynurenic acid | 0.090313 | 0.66769 | 0.9655 |
| Succinate | 0.088383 | 0.6744 | 0.9655 |
| Betaine | 0.088383 | 0.6744 | 0.9655 |
| Thymol sulfate | 0.087226 | 0.67844 | 0.9655 |
| Glycyl-tyrosine | -0.087226 | 0.67844 | 0.9655 |
| Ornithine | 0.08684 | 0.67979 | 0.9655 |
| 1-methylxanthine | -0.085296 | 0.68519 | 0.9655 |
| Glycerophosphoryl choline | 0.084524 | 0.6879 | 0.9655 |
| Lysyl-proline | 0.082208 | 0.69605 | 0.9655 |
| Choline | 0.08105 | 0.70013 | 0.9655 |
| Myristoleate | 0.079121 | 0.70696 | 0.9655 |
| Adrenate | 0.077191 | 0.71381 | 0.9655 |
| Glucose | -0.076805 | 0.71518 | 0.9655 |
| Tryptophan | 0.074875 | 0.72206 | 0.9655 |
| Arginine | 0.074103 | 0.72481 | 0.9655 |
| heptanoate (7:0) | -0.072559 | 0.73034 | 0.9655 |
| Spermine | -0.072559 | 0.73034 | 0.9655 |
| p-Hydroxyphenyllactic Acid | -0.071787 | 0.7331 | 0.9655 |
| Malic Acid | 0.07063 | 0.73726 | 0.9655 |
| Phenylacetylglutamine | -0.069858 | 0.74003 | 0.9655 |
| Nonadecanoiate | 0.069858 | 0.74003 | 0.9655 |
| Proline | 0.069858 | 0.74003 | 0.9655 |
| Pyruvate | 0.0687 | 0.7442 | 0.9655 |
| Arachidonate | 0.068314 | 0.74559 | 0.9655 |
| Glycochenodeoxycholate | 0.068314 | 0.74559 | 0.9655 |
| Methionine | 0.067928 | 0.74698 | 0.9655 |
| Cytosine | -0.06677 | 0.75116 | 0.9655 |
| γ-glutamyl-phenylalanine | -0.065226 | 0.75674 | 0.9655 |
| Oleic acid | 0.06484 | 0.75814 | 0.9655 |
| Theobromine | 0.060981 | 0.77215 | 0.9655 |
| Lactate | -0.060209 | 0.77496 | 0.9655 |
| Serine | -0.060209 | 0.77496 | 0.9655 |
| Bilirubin | 0.057893 | 0.78341 | 0.9655 |
| Xanthosine | 0.055191 | 0.7933 | 0.9655 |
| Glycerol 3-Phosphate | -0.054034 | 0.79755 | 0.9655 |
| Glycyl-proline | 0.053262 | 0.80038 | 0.9655 |
| 4-vinylphenol sulfate | 0.052876 | 0.8018 | 0.9655 |
| N6-Methyllysine | -0.05249 | 0.80322 | 0.9655 |
| Beta-Hydroxybutyrate | 0.049016 | 0.81602 | 0.9655 |
| Citrate | -0.046314 | 0.826 | 0.9655 |
| Dimethylglycine | -0.041297 | 0.84461 | 0.9655 |
| Adipate | -0.040525 | 0.84748 | 0.9655 |
| 2-Keto Tridecanoic Acid | -0.039753 | 0.85036 | 0.9655 |
| Docosahexaenoic acid | 0.039753 | 0.85036 | 0.9655 |
| 5-oxoproline | 0.037438 | 0.85899 | 0.9655 |
| Eicosenoate | -0.037438 | 0.85899 | 0.9655 |
| Linoleic acid | -0.036666 | 0.86187 | 0.9655 |
| 16-hydroxypalmitate | 0.035508 | 0.86619 | 0.9655 |
| N-formyl methionine | -0.032806 | 0.87629 | 0.9655 |
| 3-hydroxyanthranilate | -0.03242 | 0.87773 | 0.9655 |
| Phenylalanine | 0.031648 | 0.88063 | 0.9655 |
| Porphobilinogen | 0.03049 | 0.88496 | 0.9655 |
| Valine | -0.030104 | 0.88641 | 0.9655 |
| N-acetylglutamine | 0.030104 | 0.88641 | 0.9655 |
| paraxanthine | -0.027403 | 0.89655 | 0.9655 |
| 2-hydroxyisovalerate | -0.025473 | 0.9038 | 0.9655 |
| Palmitoylcarnitine | -0.025087 | 0.90525 | 0.9655 |
| L-Glutamine | 0.024315 | 0.90815 | 0.9655 |
| Methylmalonate | 0.023929 | 0.90961 | 0.9655 |
| Tyrosine | 0.022385 | 0.91542 | 0.96627 |
| Adenine | -0.020456 | 0.92269 | 0.96857 |
| Hydroquinone | 0.014666 | 0.94453 | 0.98605 |
| Indolepropionate | -0.011579 | 0.95619 | 0.98702 |
| Orotate | 0.011193 | 0.95765 | 0.98702 |
| N-acetyl-alanine | -0.008491 | 0.96787 | 0.98702 |
| Oxalate | -0.008105 | 0.96933 | 0.98702 |
| Vitamin B2 | 0.0061753 | 0.97663 | 0.98702 |
| 4-pyridoxate | -0.0061753 | 0.97663 | 0.98702 |
| Cortisol | 0.0027017 | 0.98977 | 0.98977 |
| Methylguanidine | 0.0027017 | 0.98977 | 0.98977 |

Spearman’s rank correlation was used to assess the strength and direction of associations, with statistical significance considered at *p*< 0.05. eGFR: estimated glomerular filtration rate
