# Supplementary material for: Serum metabolomics identifies gut-derived uremic toxins and bile acid dysregulation associated with chronic kidney disease severity
Source: Sci Rep. 2026 Apr 14;16:12375. doi: 10.1038/s41598-026-44271-4 (PMC13083900; doi:10.1038/s41598-026-44271-4)
Supplement: Supplementary file 10 — Supplementary Material 10 [file 41598_2026_44271_MOESM10_ESM.docx]

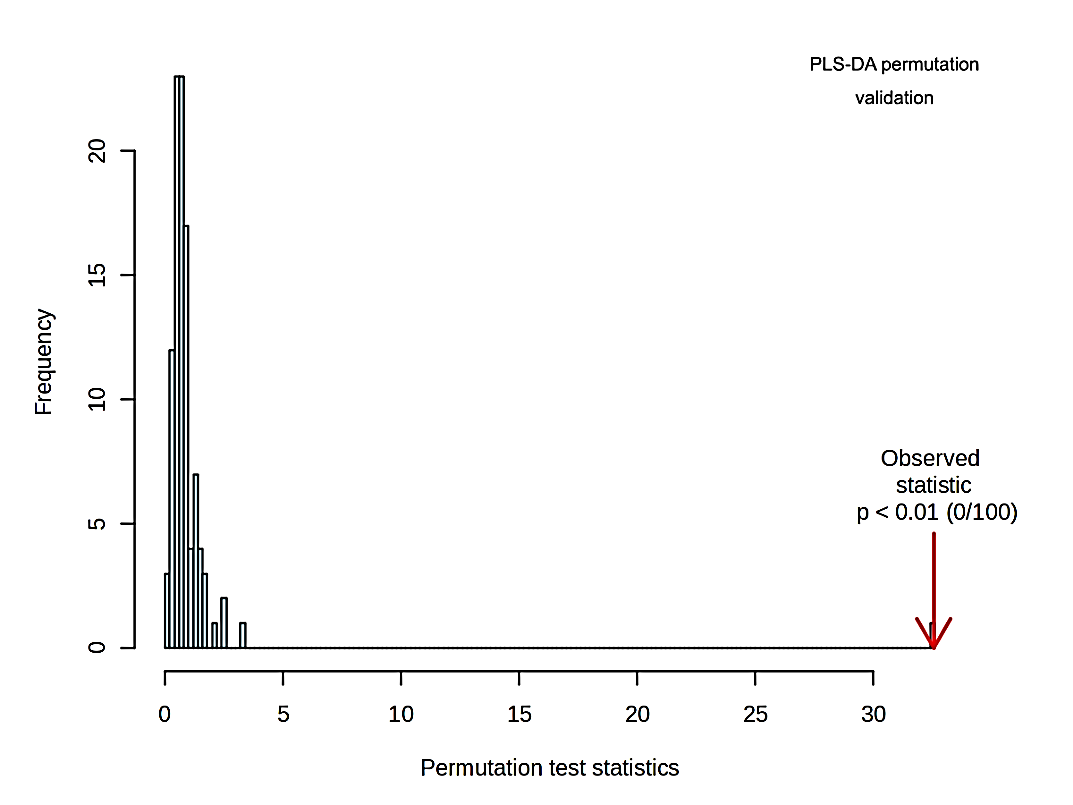


**Supplementary Fig.1a**. Permutation validation of the PLS-DA model (100 permutations) discriminating between ESKD, eCKD and NC using metabolites detected in negative ionization mode. Histogram shows the distribution of model performance after 100 random permutations of class labels. The observed model statistic (red arrow) lies far to the right of the permutation distribution (p < 0.01), indicating that the model is not overfitted.


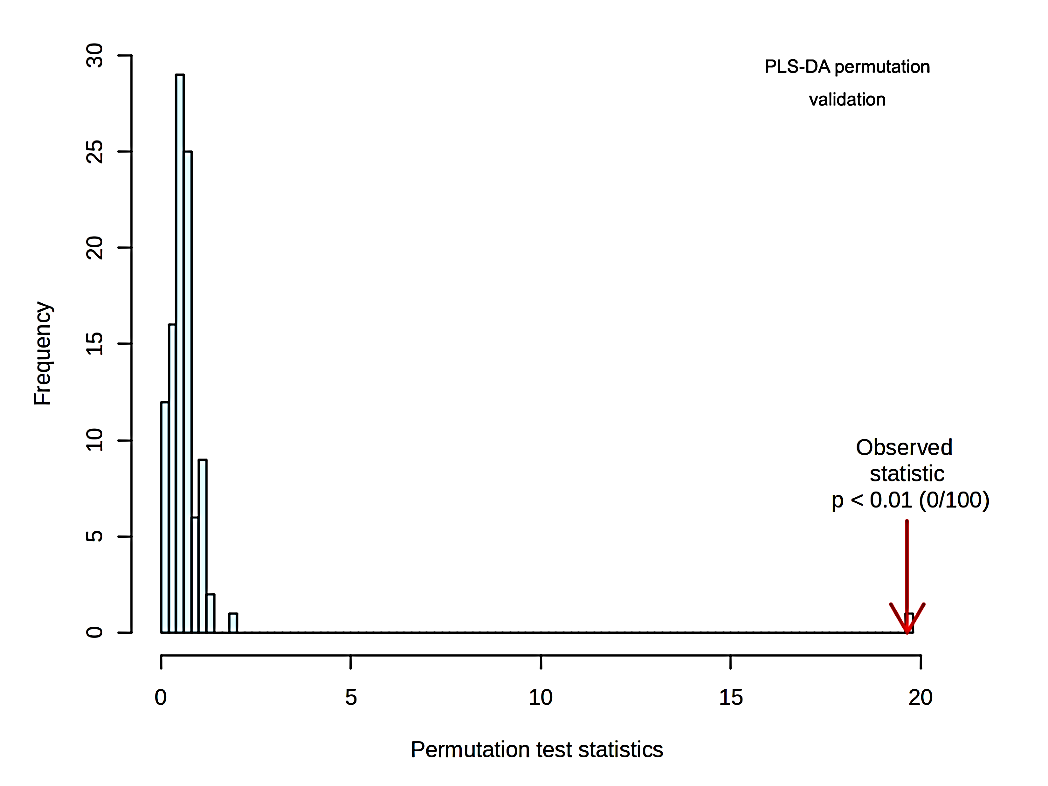


**Supplementary Fig.1b**. Permutation validation of the PLS-DA model (100 permutations) discriminating between ESKD, eCKD and NC using metabolites detected in positive ionization mode. Histogram shows the distribution of model performance after 100 random permutations of class labels. The observed model statistic (red arrow) lies far to the right of the permutation distribution (p < 0.01), indicating that the model is not overfitted.


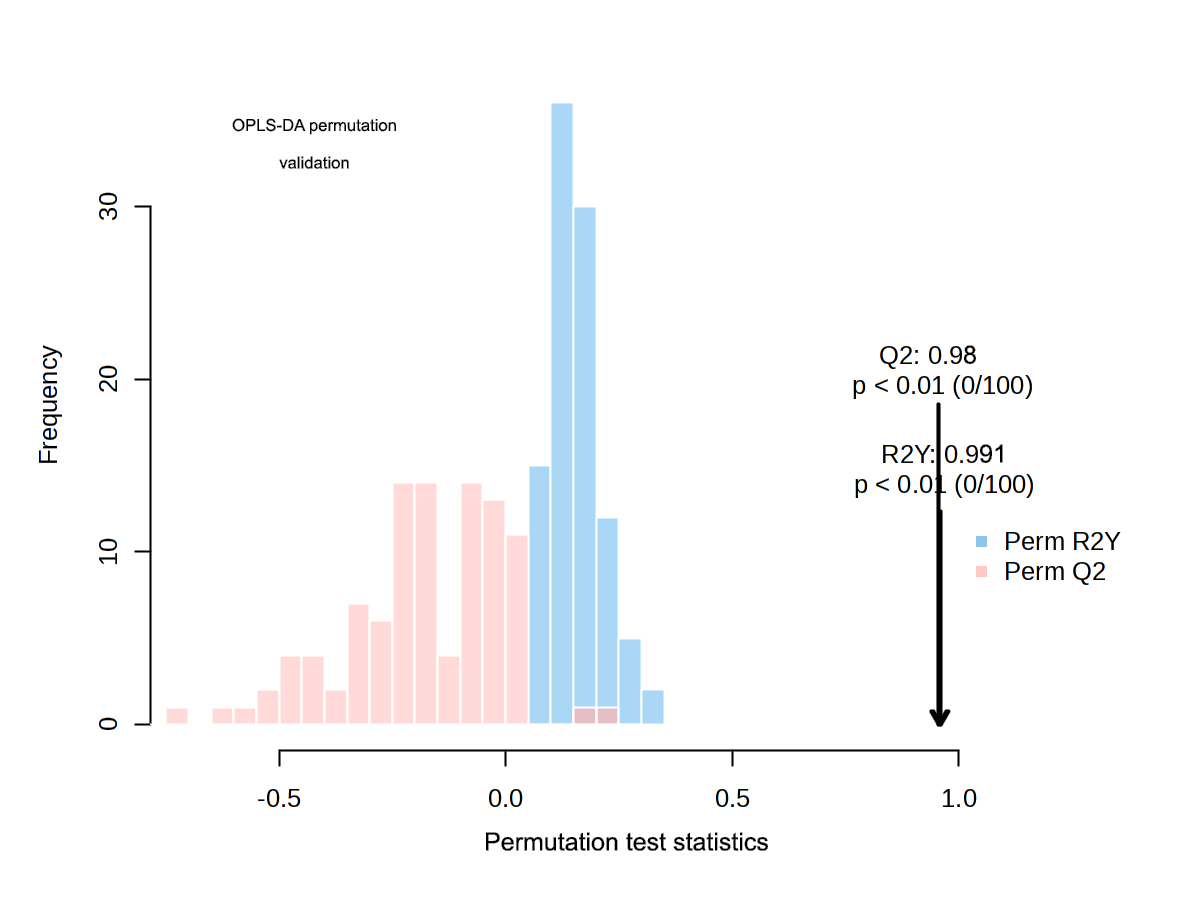


**Supplementary Fig.1c**. Permutation validation of the OPLS-DA model (100 permutations) discriminating between eCKD from NC using metabolites detected in negative ionization mode. The true R²Y and Q² values (indicated by vertical arrows) were greater than all permuted values, with p < 0.01, confirming that the models were not overfitted.


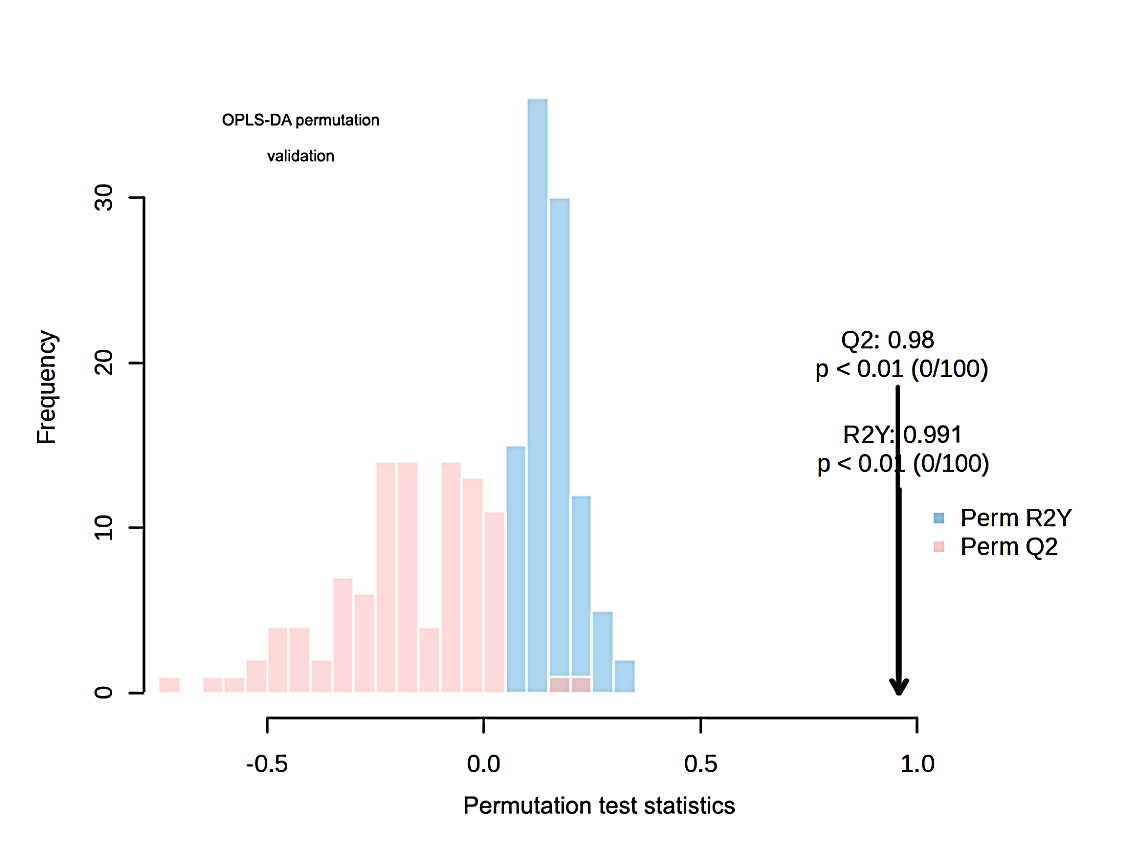


**Supplementary Fig.1d**. Permutation validation of the OPLS-DA model (100 permutations) discriminating between ESKD from NC using metabolites detected in negative ionization mode. The true R²Y and Q² values (indicated by vertical arrows) were greater than all permuted values, with p < 0.01, confirming that the models were not overfitted.


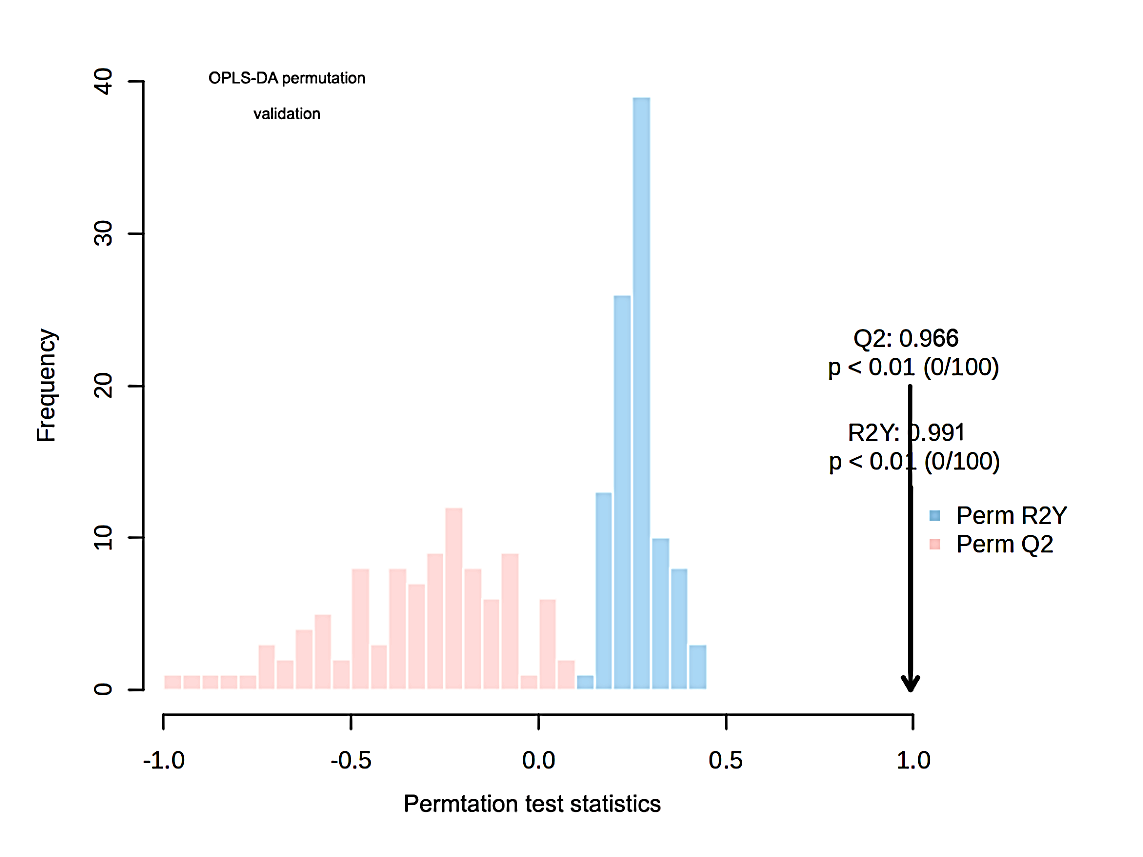


**Supplementary Fig.1e**. Permutation validation of the OPLS-DA model (100 permutations) discriminating between eCKD from NC using metabolites detected in positive ionization mode.The true R²Y and Q² values (indicated by vertical arrows) were greater than all permuted values, with p < 0.01, confirming that the models were not overfitted.


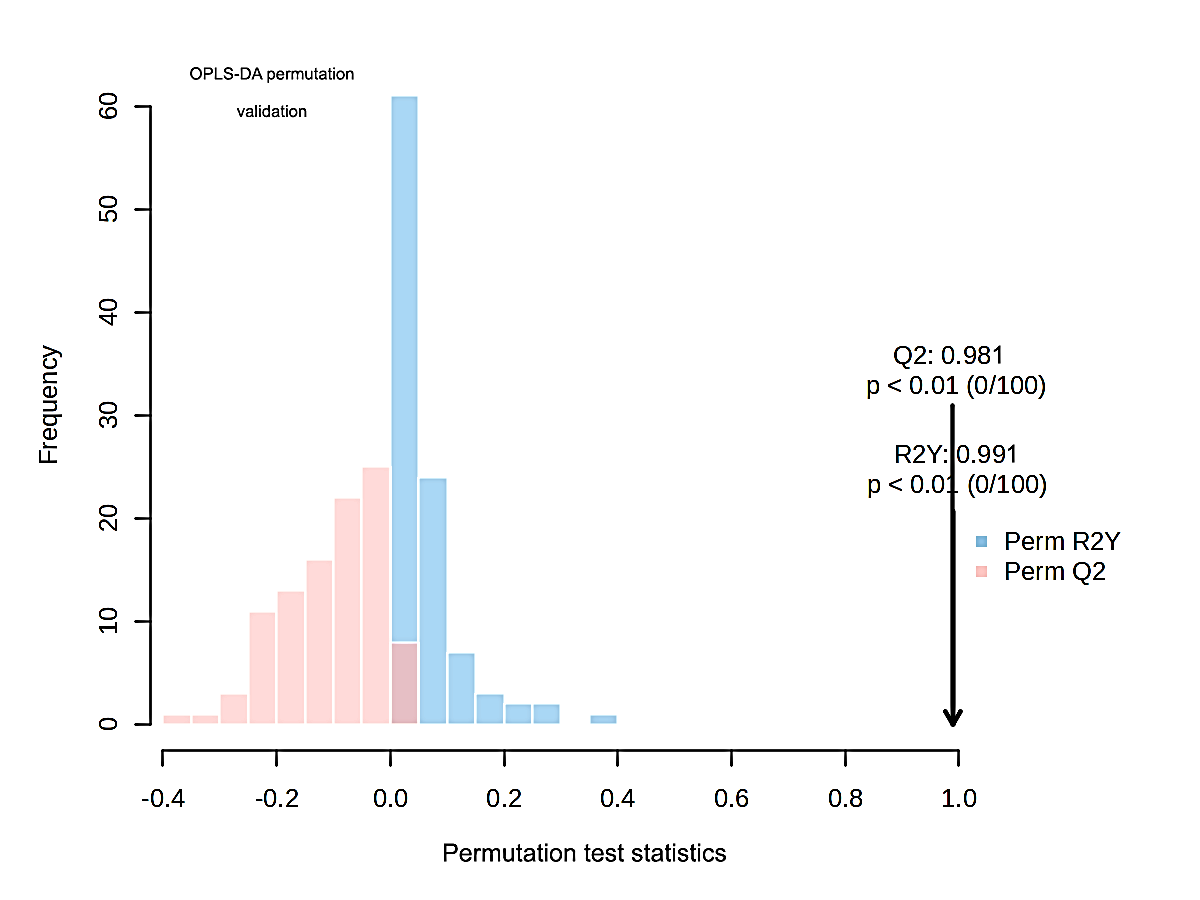


**Supplementary Fig.1f**. Permutation validation of the OPLS-DA model (100 permutations) discriminating between ESKD from NC using metabolites detected in positive ionization mode.The true R²Y and Q² values (indicated by vertical arrows) were greater than all permuted values, with p < 0.01, confirming that the models were not overfitted.


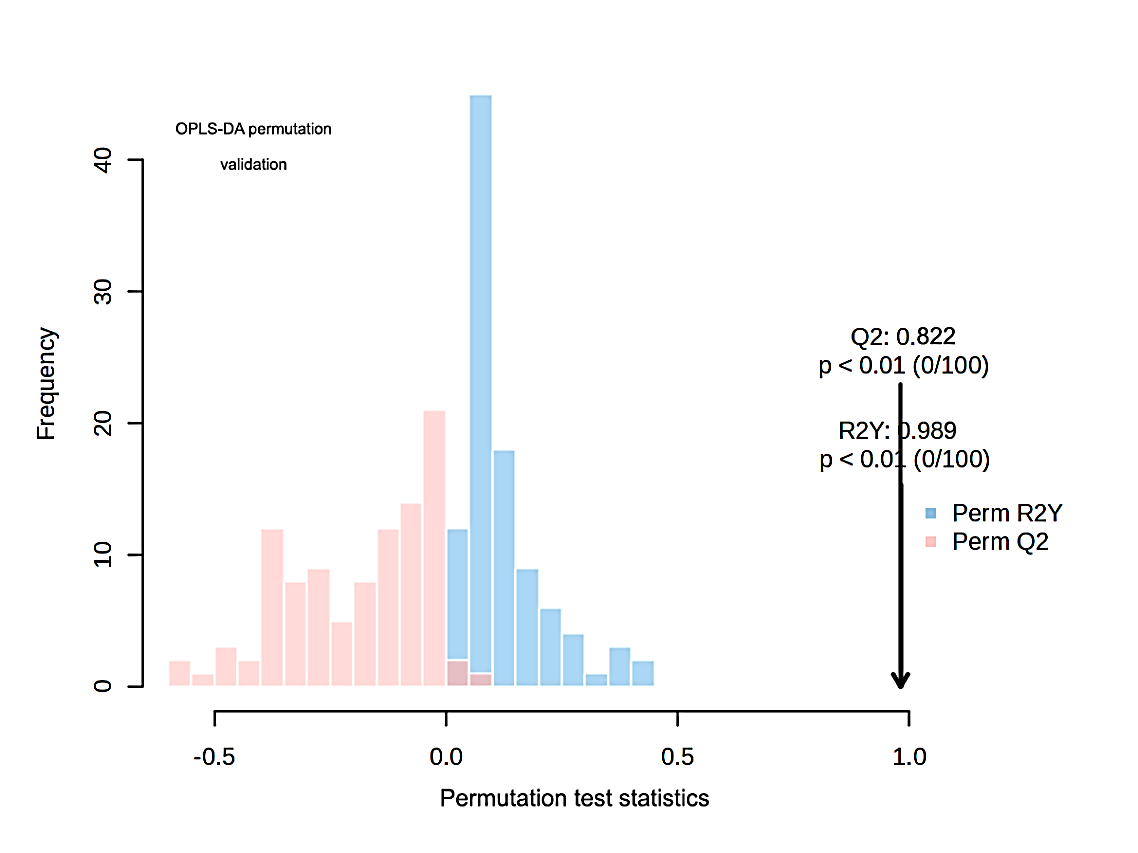


**Supplementary Fig.1g**. Permutation validation of the OPLS-DA model (100 permutations) discriminating between ESKD from eCKD using metabolites detected in negative ionization mode.The true R²Y and Q² values (indicated by vertical arrows) were greater than all permuted values, with p < 0.01, confirming that the models were not overfitted.


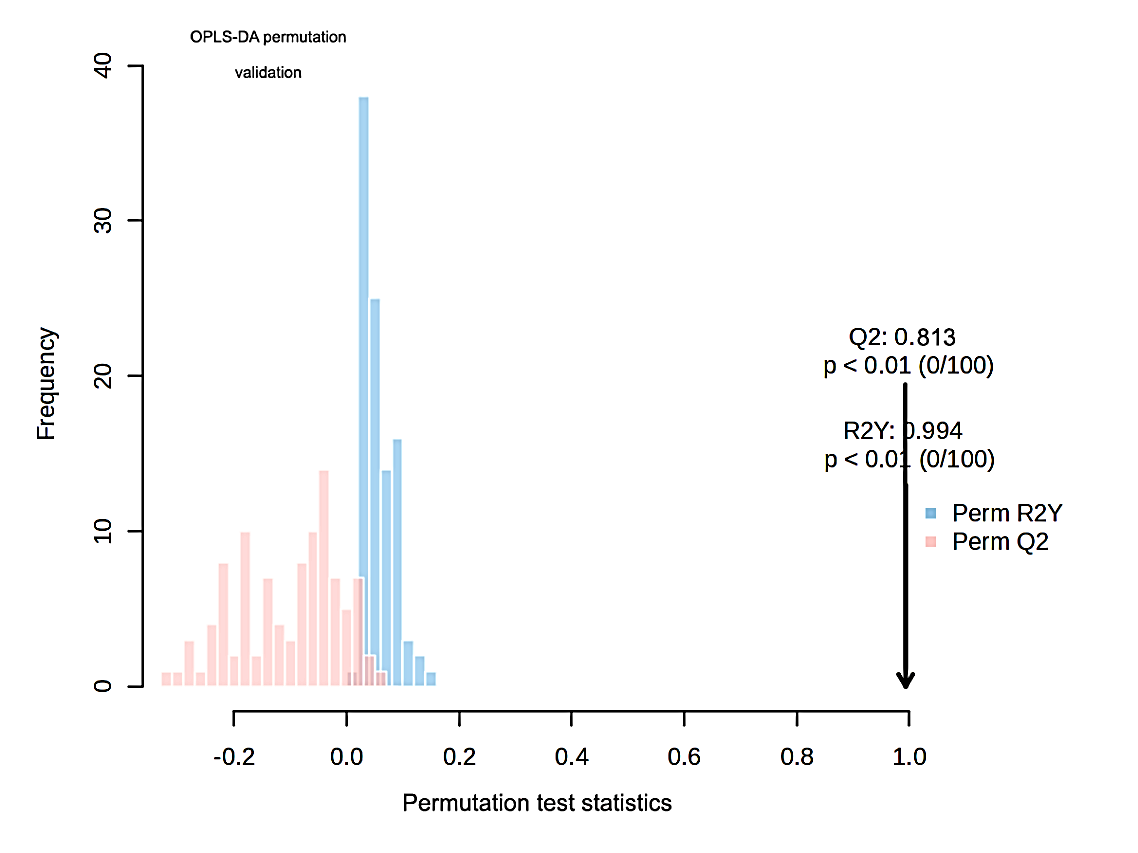


**Supplementary Fig.1h**. Permutation validation of the OPLS-DA model (100 permutations) discriminating between ESKD from eCKD using metabolites detected in positive ionization mode. The true R²Y and Q² values (indicated by vertical arrows) were greater than all permuted values, with p < 0.01, confirming that the models were not overfitted.
